# A mechano-integrated gradient electrolyte for long-cycling solid-state lithium metal batteries

**DOI:** 10.1038/s41467-026-74573-0

**Published:** 2026-06-24

**Authors:** Xiaoping Yi, Guoqing Qi, Wending Pan, Kaishan Xiao, Yixin Yang, Yang Yang, Bitong Wang, Xiaolong Zhao, Xunliang Liu, Hong Li

**Affiliations:** 1 School of Energy and Environmental Engineering, University of Science and Technology Beijing, Beijing, China; 2 Taihang Institute of Advanced Energy Storage Technology, Baoding, Hebei China; 3 School of Energy and Power Engineering, Dalian University of Technology, Dalian City, Liaoning Province China; 4 Department of Mechanical Engineering, The University of Hong Kong, Hong Kong, China; 5 Beijing Key Laboratory for New Energy Materials and Devices, Institute of Physics, Chinese Academy of Sciences, Beijing, China; 6 College of Materials Science and Optoelectronic Technology, University of Chinese Academy of Sciences, Beijing, China; 7 Zhejiang Key Laboratory of All-Solid-State Battery, Ningbo Key Laboratory of All-Solid-State Battery, Ningbo, Zhejiang China

**Keywords:** Batteries, Batteries, Energy, Batteries

## Abstract

Overcoming interfacial mechano-electrochemical failure remains a fundamental challenge in solid-state lithium metal batteries, where polymers offer conformal interfacial contact but suffer from low ionic conductivity, while oxides/sulfides provide high ionic conductivity but face severe interfacial issues. Here we show a mechano-integrated gradient electrolyte based on a hydrogen-bonded polyurethane matrix with dual chain extenders. The polyurethane matrix exhibits high viscoelasticity (>5000% fracture strain) and self-healing, allowing high filler loading and continuous triphasic lithium-ion percolation networks. A spatially graded Li_1.3_Al_0.3_Ti_1.7_(PO_4_)_3_ architecture (10–100 wt%) decouples interfacial requirements: conformal contact with lithium metal negative electrode, high ionic conductivity (~10^−4^ S cm^−1^), and an electrochemical stability window up to 4.9 V. The homologous polymer framework eliminates chemo-mechanical degradation while providing mechanical strength (>80 MPa) and solution processability. This integrated design suppresses interfacial delamination and dendrite growth (>7500 h of stable lithium plating/stripping), and mitigates positive electrode degradation (74% capacity retention after 1000 cycles in Li | |LiFePO_4_ cells at 0.5 C and stable operation in stack-pressure-free NCM811 pouch cells). This work provides a scalable platform for high-energy-density, long-lifespan solid-state lithium metal batteries.

## Abstract (web summary)

Solid-state lithium batteries suffer from interfacial instability. Here, authors develop a mechano-integrated gradient electrolyte with a viscoelastic polymer and graded filler architecture, enabling stable lithium plating/stripping and Li | |LiFePO_4_ cell cycling, while supporting high speed roll-to-roll manufacturing.

## Introduction

The ongoing transition toward solid-state lithium metal batteries (SSLMBs), represents a paradigm shift in energy storage technology, aiming for high safety and high-energy density in applications ranging from electric vehicles to grid-scale storage systems^1–3^. While inorganic superionic conductors such as Li_3_Ta_3_O_4_Cl_10_ (13.7 mS cm^−1^ at 25 °C), Li_9.54_Si_1.74_P_1.44_S_11.7_Cl_0.3_ (25 mS cm^−1^ at 25 °C), and Li_9.54_[Si_0.6_Ge_0.4_]_1.74_P_1.44_S_11.1_Br_0.3_O_0.6_ (32 mS cm^−1^ at 25 °C) have achieved ionic conductivities rivaling those of liquid electrolytes^4–6^, their practical deployment is hindered by interfacial mechano-electrochemical instability, often necessitating high stack pressures (>10 MPa) that complicate cell integration and raise manufacturing costs^7–9^. Solid polymer electrolytes (SPEs), notably poly(ethylene oxide) (PEO)-based systems, offer flexibility and processability but face a well-known trade-off between mechanical strength (<1.0 Mpa) and ionic conductivity (<10^−5^ S cm^−1^ at 25 °C), along with limited electrochemical stability (<4.0 V)^10–12^. Although composite solid electrolytes (CSEs) incorporating inorganic fillers such as Li_1+*x*_Al_*x*_Ti_2-*x*_(PO_4_)_3_ (LATP) and Li_7_La_3_Zr_2_O_12_ (LLZO) have been explored to enhance conductivity and stability, conventional designs fail to simultaneously achieve high filler loading, robust adhesion, and graded mechanical properties, resulting in poor processability, inadequate electrode contact, and incompatibility with scalable roll-to-roll manufacturing^13–15^. Critically, the lithium metal negative electrode requires an electrolyte with high deformability and adaptive viscoelasticity to maintain conformal contact throughout repeated plating/stripping cycles, whereas the high-voltage positive electrode demands a dense, pinhole-free, and ceramic-rich barrier against oxidation and transition-metal dissolution, while still preserving sufficient interfacial contact through the continuous polymer phase.

The core challenge lies in the intrinsic property mismatch between inorganic fillers and polymer matrices. Ceramics such as LLZO and LATP provide high modulus and ionic conductivity but are brittle, fracturing under strains greater than 300% during lithium plating/stripping^16,17^. In contrast, soft polymers (e.g., PEO, poly(vinylidene fluoride) (PVDF)) exhibit viscoplastic creep and low storage modulus, leading to interfacial delamination and dendritic failure^1,18,19^. Multilayer heterostructures have been proposed to decouple functionality, yet they introduce high interfacial impedance and localized stress concentration, limiting their cyclability under practical conditions^15,20^. More fundamentally, state-of-the-art CSEs face three material-level constraints: (1) limited fracture strain (<500%) causes progressive loss of interfacial contact, leading to rapid capacity fade^21,22^; (2) ionic conductivity typically saturates below 10^−4^ S cm^−1^, restricting rate performance and necessitating elevated operating temperatures^23,24^; and (3) beyond ~60 wt% filler loading, percolation networks collapse due to agglomeration, causing severe embrittlement and loss of processability^25–28^. Together, these issues prevent the integration of high conductivity, lithium-conformal interfaces, and high-voltage stability within a single electrolyte structure, a gap that has persistently limited the performance and scalability of SSLMBs.

In this work, we report a Mechano-Integrated Gradient Electrolyte (MIGE) that coalesces molecular design, microstructural engineering, and scalable manufacturing into a unified platform. We engineer a viscoelastic polyurethane matrix (PUDA) with dynamic hydrogen-bonding motifs, achieving >5000% fracture strain and high filler-loading capacity. This enables the construction of a monolithic electrolyte film with a spatially graded LATP architecture (10–100 wt%), which decouples interfacial demands: a compliant negative electrode layer ensures conformal lithium contact, an intermediate percolation layer facilitates rapid ion transport (~10^−4^ S cm^−1^), and a ceramic-rich positive electrode interface delivers oxidation stability beyond 4.9 V. The single-phase polymer framework circumvents interfacial degradation while providing high tensile strength (>80 MPa). This work establishes a design framework for solid electrolytes that directly addresses the core mechano-electrochemical coupling issue, offering a scalable, solution-processable route to high-performance, pressure-free SSLMBs.

## Results

### Molecular design and mechano-integrated gradient architecture

**Fig. 1:**
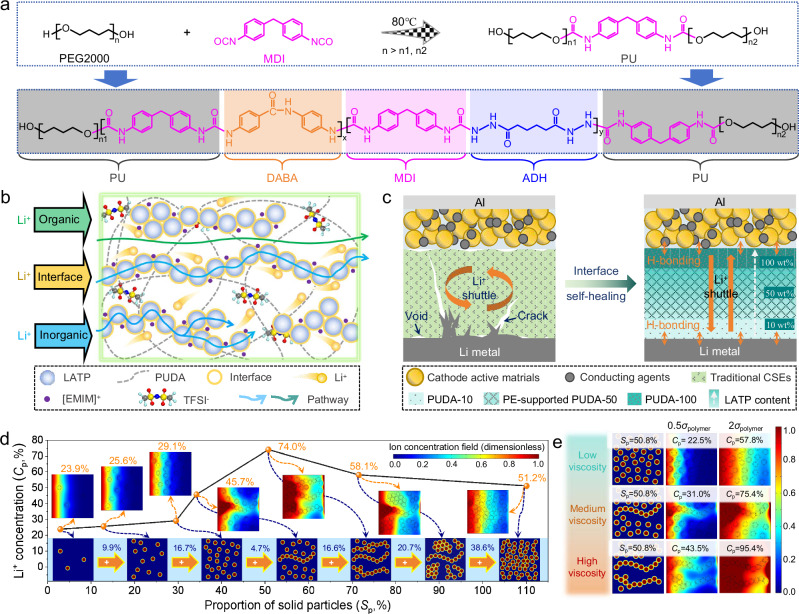
Molecular design and ion-transport mechanism of the mechano-integrated gradient electrolyte (MIGE). **a** Synthesis scheme of the PUDA polymer. PUDA: polyurethane-based electrolyte with dual hydrogen-bonding extenders. **b** Schematic of triphasic ion-transport pathways within the PUDA-based composite electrolyte. The polymer matrix is indicated by a dashed gray line, the Li_1.3_Al_0.3_Ti_1.7_(PO_4_)_3_ (LATP) filler by blue circles, and the localized ionic liquid molecules (purple dots) at interfaces aid particle dispersion. **c** Structural comparison between a conventional laminated configuration with composite solid electrolytes (CSEs) and the proposed MIGE-based solid-state lithium metal battery (SSLMB) configuration. **d** COMSOL-simulated Li^+^ flux profiles as a function of inorganic filler content, expressed by the area fraction of solid particles relative to the polymer matrix (dimensionless parameter *S*_p_). **e** Ion-transport efficiency of homogeneous, single-percolation, and multichannel-percolation architectures at equivalent filler content under different polymer-phase ionic conductivities.

Overcoming the trade-off between interfacial conformability and electrochemical stability in solid electrolytes remains a pivotal challenge. To address this, we designed a dynamically cross-linked polyurethane (PU) matrix through molecular-level topological engineering. The PU backbone was synthesized via step-growth polymerization of polyol and diisocyanate precursors (Fig. 1a), incorporating urea linkages (–NH–COO–) as key structural motifs. The soft segments, composed of poly(ethylene glycol) (PEG, M_w_ = 2000), feature flexible ether-oxygen (–C–O–C–) chains that promote high chain mobility and remain in an amorphous, elastomeric state, enabling large deformation and facilitating Li^+^ solvation. In parallel, rigid segments derived from methylene diphenyl diisocyanate (MDI) provide essential mechanical reinforcement. The strategic introduction of hydrogen-bond-rich extenders, 4,4′-diaminobenzanilide (DABA) and adipic dihydrazide (ADH), enables the formation of a hierarchical supramolecular architecture (labeled as PUDA). DABA, featuring a biphenyl group with high rigidity and steric hindrance, significantly enhances the regularity of hard segments, serving as a robust physical cross-linking point that reinforces the polymer skeleton. ADH serves as a hydrogen-bonding hub, containing two amide groups (–NH–CO–) that act as both donors and acceptors, enabling the formation of a dense, dynamically reversible hydrogen-bond network with carbonyl and imino groups across neighboring chains. Together, DABA and ADH establish a recurrent –NH–CO–NH– motif that imparts high viscoelasticity (fracture strain >5000%), autonomous self-healing, and high processability, accommodating high inorganic filler loadings without compromising film integrity. As illustrated in Fig. 1b, the resulting PUDA-based CSE facilitates continuous triphasic percolation pathways across organic, inorganic, and organic–inorganic interphase regions, enabling efficient Li^+^ transport throughout the multilayer gradient structure. A controlled amount of 1-ethyl-3-methylimidazolium bis(trifluoromethylsulfonyl)imide ([EMIM][TFSI]) was added to the precursor solution to enhance interfacial stability and ionic conductivity. The strong adhesive nature of the PUDA matrix, combined with interfacial plasticization by the ionic liquid, is designed to minimize the Li^+^ migration barrier at the organic–inorganic boundary. This engineered interphase, coupled with the formation of a percolated LATP network, enables efficient Li^+^ transport throughout the multilayer gradient structure.

Building on this molecularly tailored matrix, we constructed a Mechano-Integrated Gradient Electrolyte (MIGE, Tri-PUDA-*x*@LATP) in which the LATP filler content is programmed to vary continuously across the membrane thickness, as schematically contrasted in Fig. 1c. The MIGE architecture embodies the mechano-integrated principle through coherent design across three scales: (i) Molecular integration via dynamic hydrogen bonds, enabling both high elongation and high tensile strength; (ii) Microstructural integration via a continuous LATP gradient, ensuring smooth spatial transitions in mechanics and Li^+^ flux; and (iii) Functional integration, where compliant negative electrode contact, rigid dendrite suppression, and dense positive electrode protection are unified within a single, ionically continuous membrane. Compatible with roll-to-roll manufacturing, the MIGE is formed by sequential coating the functional layers onto a thin (~12 μm), porous polyethylene (PE) substrate (see Supporting Information for details). The final freestanding MIGE membrane has a total thickness of approximately 40 μm. The filler loading (*x* in PUDA-*x*) is defined as the weight percentage of LATP relative to the PUDA polymer matrix. The architecture comprises three functionally distinct layers: an negative electrode-facing layer (PUDA-10, 10 wt% LATP) that is polymer-dominated, designed to achieve atomically conformal contact with Li metal while isolating the majority of the oxide filler from direct reduction at the negative electrode interface; an ion-transport interlayer (PUDA-50, 50 wt% LATP) infused into a porous PE scaffold, which forms a continuous 3D percolating network to ensure high ionic conductivity and a positive electrode-facing layer (PUDA-100, 100 wt% LATP) engineered as a ceramic-dense barrier, where LATP particles forms a continuous protective layer. This structure enabled by PUDA’s high viscoelasticity establishs the electrochemically stable LATP as the primary interface. Unlike conventional composites with homogenized fillers or simply stacked heterostructures, MIGE is a continuously graded, monolithic architecture. In this design, mechanical properties, such as modulus, adhesion, and elongation, are spatially tailored to meet the distinct demands of each electrode interface, without introducing additional internal boundaries that create stress concentrations. This is realized by synergistically coupling a single-phase, elastic polymer matrix with a continuously graded filler distribution, thereby embedding mechanical adaptation directly into the ion-conduction network.

To elucidate the ion-transport mechanisms underlying the MIGE performance, we conducted multiscale theoretical modeling of Li^+^ diffusion in PUDA-based CSEs across varying filler contents. COMSOL simulations based on a triphasic structural model (organic/inorganic/interphase) reveal a non-monotonic relationship between filler content and Li^+^ flux (Fig. 1d), which is consistent with previous reported trends^25–30^. In the phase-field simulations, the filler content is represented by the dimensionless parameter *S*_p_, defined as the area fraction of the inorganic filler and interphase relative to the polymer matrix. *S*_p_ is qualitatively proportional to, but distinct from, the experimental LATP weight percentage (wt%). At low filler contents (*S*_p_ < 30%), ion transport occurs predominantly through the organic phase, with conductivity increasing gradually as filler concentration rises. A maximum Li^+^ concentration (*C*_p_ = 74.0%) is achieved at the triphasic percolation threshold (*S*_p_ = 50.8%), where continuous Li^+^ transport pathways are established across all three phases. Beyond this threshold, particle agglomeration disrupts percolation networks and compresses organic-phase pathways, thereby degrading ionic transport. Figure 1e further compares ion-transport efficiency under different filler distributions at identical loading (*S*_p_ = 50.8%). In low-viscosity polymers/solvents, inorganic particles tend to disperse uniformly, enabling isotropic diffusion but limiting directional transport. Single-percolation architectures enhance conduction along inorganic–inorganic interfaces yet remain kinetically constrained. In contrast, the high viscosity of PUDA promotes the interconnection of inorganic particles into continuous ion-transport channels. Our multichannel percolation design establishes interconnected transport highways, achieving *C*_p_ values more than 1.6 times and 1.2 times higher than those of zero- and single-channel configurations, respectively (Supplementary Fig. 1 and Supplementary Fig. 2). This result underscores that topological connectivity of ion channels, more than homogeneous dispersion, governs conduction behavior in composite electrolytes. Further analysis quantifies the synergy between percolation channel density and polymer-phase conductivity (*σ*_polymer_). Enhancing *σ*_polymer_ can yield greater conductivity gains in percolated systems than in uniformly dispersed composites. At *σ*_polymer_, single- and double-percolated systems exhibit ionic conductivities 14.8% and 23.2% higher than the zero-percolated system, respectively. Together, the intrinsic viscoelasticity of PUDA, enabled by synergistic dual-extender design, combined with a rationally engineered gradient filler distribution, ensures adaptive interfacial contact and sustained electrode cohesion during cycling. This synergistic multi-scale strategy, integrating tailored molecular architecture with spatially graded functionality, provides a viable pathway toward high-performance SSLMBs.

### Structural and mechano-chemical characterization of gradient PUDA-*x* electrolytes

**Fig. 2:**
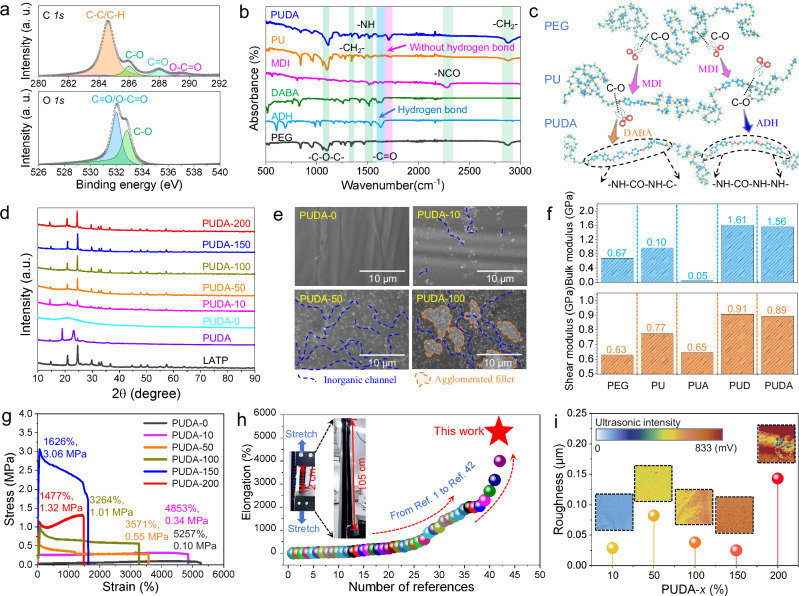
Structural and mechanical characterization of PUDA-based electrolytes. **a** X-ray photoelectron spectroscopy (XPS) spectra of PUDA. **b** Fourier-transform infrared (FT-IR) spectra of PEG, ADH, DABA, MDI, PU, and PUDA. PEG: polyethylene glycol; ADH: adipic dihydrazide; DABA: 4,4’-diaminobenzanilide; MDI: methylene diphenyl diisocyanate; PU: polyurethane without extenders. **c** Binding structure diagram of key molecular segments based on density functional theory (DFT) calculations. Different colored spheres represent atoms: blue, C; red, N; yellow, O; gray, H. **d** X-ray diffraction (XRD) patterns of LATP, PUDA, and PUDA-*x* composites. **e** Surface scanning electron microscopy (SEM) images of PUDA-*x* electrolytes. **f** Bulk and shear moduli of the polymer series: PEG, PU, PUA, PUD, and PUDA. **g** Stress-strain curves of PUDA-*x* composites. The filler-loading *x* is defined as the weight percentage of LATP relative to the PUDA polymer matrix. **h** Comparison of elongation at break (fracture strain) between PUDA-0 and previously reported polymer electrolytes. The source of the literature data shown in this figure can be found in Supplementary Information, Table 1. **i**, Surface roughness profiles and ultrasonic mapping images of PUDA-*x* electrolytes.

The composition-dependent structural evolution and mechanical properties of the Tri-PUDA-*x*@LATP electrolyte were systematically characterized to validate its gradient architecture and multifunctional performance. X-ray photoelectron spectroscopy (XPS) confirmed the chemical structure of the PUDA matrix (Fig. 2a). The C 1 s spectrum exhibited characteristic peaks corresponding to C–C/C–H (284.6 eV) and C–O (286.0 eV) from PEG segments, C=O (288.0 eV) from DABA, ADH, and MDI units, and O–C=O (289.5 eV) from the PU backbone. The O 1 s spectrum further validated the synthesis, showing contributions from C−O bonds (533.6 eV) and carbonyl groups (531.9 eV). Fourier-transform infrared (FT-IR) spectroscopy revealed the formation of dynamic hydrogen-bonding networks (Fig. 2b), evidenced by the disappearance of the isocyanate -NCO peak (2264 cm^−1^) in both PU and PUDA, confirming complete reaction of PEG and MDI. Key vibrational modes included C–O–C (1102 cm^−1^), –C=O (1719 cm^−1^), –NH (1540 cm^−1^), and –CH_2_–(1370 cm^−1^). DABA and ADH are rich in -NH. The split –C=O peak, with a component blue-shifted to ~1680 cm^−1^, indicated strong hydrogen bonding within –NH–COO– groups. Density functional theory (DFT) suggested the presence of intermolecular interactions, with computed binding energies of −2.0 eV for MDI-PEG, −3.0 eV for DABA-MDI, and −3.5 eV for ADH-MDI (Fig. 2c and Supplementary Fig. 3). These values indicate potentially favorable bonding interactions, although DFT calculations rely on idealized models and do not fully account for temperature or dynamic effects present in the actual material. The conjugated biphenyl groups in DABA are expected to promote π–π stacking, which likely enhances the cohesion and mechanical strength of the hard segments. Its rigid structure may serve as a physical cross-linking anchor, while ADH likey acts as a reversible binder, forming a dense and dynamic hydrogen-bonding network that enables efficient energy dissipation and structural reorganization. Together, they appear to create a hard-segment architecture characterized by a rigid framework and dynamic network, where DABA helps prevent irreversible chain slippage under high deformation, and ADH may redistribute localized stress through reversible bond breaking and reformation.

The viscoelastic PUDA matrix demonstrated good compatibility with LATP, forming uniform PUDA-*x* composite films with suppressed over-crosslinking and enhanced film integrity compared to pure PUDA (Supplementary Fig. 4 and Supplementary Fig. 5). X-ray diffraction (XRD) analysis (Fig. 2d) revealed complete dissociation of LiTFSI in PUDA-0, with no crystalline salt peaks. The transition from sharp diffraction peaks in pure PUDA to a broad amorphous halo in PUDA-0 signifies a salt-induced amorphization of the polymer matrix. This common effect in polymer electrolytes arises from the strong coordination of Li^+^ to polar groups (e.g., –C–O–C– in PEG, –NH–CO– in ADH/DABA) and the steric presence of TFSI^-^, which collectively disrupt polymer chain packing. The resulting amorphous structure enhances segmental mobility and facilitates Li^+^ transport. Increasing LATP content further disrupted polymer ordering, with dominant inorganic signatures emerging above 50 wt%, indicating a surface transition toward ceramic-rich characteristics. Image analysis of the scanning electron microscopy (SEM) micrographs (Fig. 2e) reveals a spatially heterogeneous microstructure with two distinct regions: interconnected LATP networks that provide continuous pathways for Li^+^ transport, and isolated filler agglomerates where particle clustering and polymer-phase compression increase ionic-transport tortuosity. This agglomeration becomes more pronounced at higher LATP loadings of 150 wt% and 200 wt% (Supplementary Fig. 6). Notably, even at 200 wt%, the PUDA-200 film remains crack-free, whereas PEO-based counterparts fail to form coherent films beyond 100 wt% (Supplementary Fig. 7). Mechanical analysis using a classical Hill model revealed that bulk and shear moduli, which quantify resistance to uniform compression and shape deformation, respectively, exhibit an inverse relationship with molecular chain mobility. While PEG-derived PU showed enhanced rigidity after MDI crosslinking, ADH contributed to flexibility, and DABA promoted stiffness (Fig. 2f). The chain segment atomic structure of PEG, PU, PUA, PUD, and PUDA is shown in Supplementary Fig. 8. The optimized mechanical profile of PUDA, characterized by balanced bulk (1.56 GPa) and shear (0.89 GPa) moduli coupled with high viscoelasticity, may contribute to its multifunctional role. The enhanced modulus, imparted by the rigid segments (MDI and DABA) and the supramolecular hydrogen-bonding network, is suggested to help the composite resist lithium dendrite penetration. Crucially, the modulus likely ensures dimensional stability and prevents excessive creep, while the viscoelasticity guarantees conformal contact with lithium metal during cycling. Furthermore, this combination of cohesion and deformability appears to underpin PUDA’s high efficacy as a binder, enabling robust adhesion to the porous PE scaffold and homogeneous integration of LATP particles. This facilitates the construction of continuous ion-transport pathways within a freestanding, flexible film, addressing a core limitation in conventional composite electrolytes where high filler content typically compromises mechanical integrity and processability. The pristine PUDA-0 exhibits a fracture strain of >5000% (Fig. 2g), capable of stretching to over fifty times its original length. For comparison, values for previously reported polymer electrolytes are listed in Fig. 2h and Supplementary Table 1. This high viscoelasticity, coupled with autonomous self-healing, provides the fundamental property required for processing high-filler-load composites. Such adhesion not only prevents interfacial delamination but also enables effective stress dissipation during cycling, crucial for sustaining continuous ion-transport pathways. Tensile strength increased systematically with LATP loading, reaching 3.06 MPa for PUDA−150 while maintaining high extensibility (~1626%). The positive electrode-facing layer (PUDA-100, 100 wt% LATP) forms a ceramic-rich yet polymer-bonded composite that serves as a dense oxygen barrier while retaining substantial deformability (~3264% strain). Even at 200 wt% LATP, where incipient agglomeration reduces tensile strength to 1.32 MPa, elongation remains substantial (~1477%), whereas conventional PEO-based electrolytes exhibit much lower elongation^19,31^. Surface topology analysis (Fig. 2i) confirmed good interfacial compatibility, with PUDA-*x* maintaining a low average roughness (<0.1 μm) for *x* ≤ 150 wt%, essential for seamless electrode contact. A non-monotonic roughness profile reflected optimal LATP dispersion prior to agglomeration at 200 wt%, beyond which film uniformity and interfacial integrity were compromised.

### Electrochemical stability and mechanical robustness of PUDA-*x* electrolytes

**Fig. 3:**
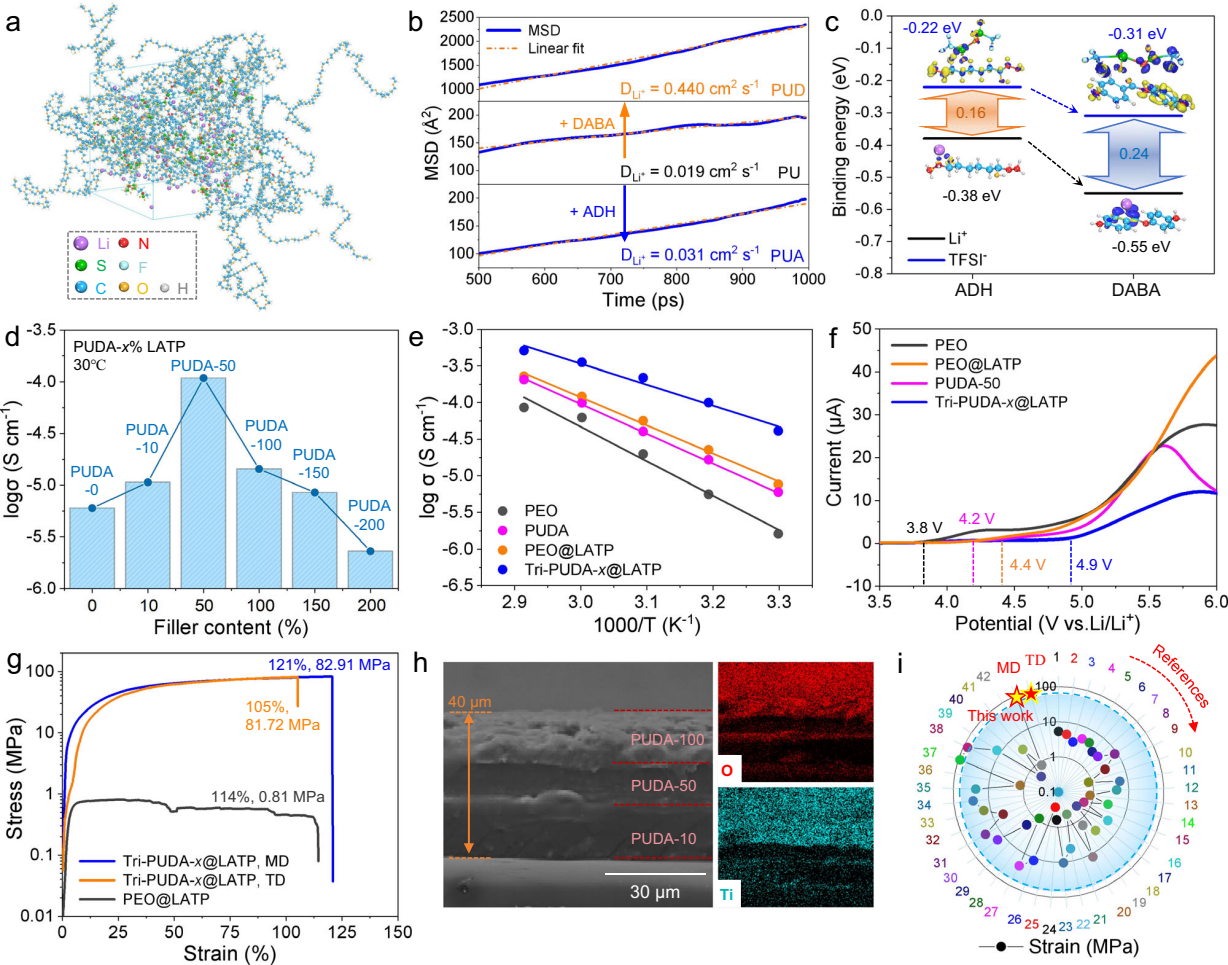
Electrochemical and mechanical performance of PUDA-based electrolytes. **a** Molecular structure of the PUDA-based electrolyte. **b** Mean squared displacement (MSD) from molecular dynamics (MD) simulations illustrating ion diffusion kinetics influenced by DABA and ADH. **c** Intermolecular interaction energies among DABA, ADH, Li^+^, and TFSI^-^ species. Different colored spheres represent atoms: purple, Li; green, S; blue, C; red, N; sky blue, F; yellow, O; gray, H. Blue and yellow regions denote electron accumulation and depletion, respectively. **d** Ionic conductivity of PUDA-*x* electrolytes as a function of LATP content. **e** Arrhenius plots of PEO, PUDA, PEO@LATP, and Tri-PUDA-*x*@LATP. **f** Linear sweep voltammetry curves comparing the electrochemical stability of PEO, PEO@LATP, PUDA-50, and Tri-PUDA-*x*@LATP. **g** Stress-strain curves of PEO@LATP, and Tri-PUDA-*x*@LATP. **h** Cross-sectional SEM image and energy-dispersive X-ray spectroscopy (EDS) elemental mapping of Tri-PUDA-*x*@LATP. **i** Tensile strength comparison of Tri-PUDA-*x*@LATP with literature data. The source of the literature data shown in this figure can be found in Supplementary Information, Table 1.

Building on the confirmed structural gradation, we next evaluate how this spatially designed architecture translates into graded electrochemical and mechanical functionality. The performance of the Tri-PUDA-*x*@LATP composite was systematically compared with conventional PEO-based CSEs. Molecular dynamics (MD) simulations indicated that lithium-ion self-diffusion coefficients increase from 0.019 (PU) to 0.031 (PUA) and 0.440 cm^2^ s^−1^ (PUD) under the simulated conditions (Fig. 3a, b). These trends suggest that incorporating DABA and ADH may enhance ion transport within the PU system. Snapshots of the molecular structures of PEG, PU, PUA, PUD, and PUDA-based solid electrolytes are shown in Supplementary Figs. 9–11. Binding energy calculations from DFT provided values of −0.38 eV for ADH–Li^+^, −0.55 eV for DABA–Li^+^, −0.22 eV for ADH–TFSI^-^, and −0.31 eV for DABA–TFSI^-^ which are suggestive of moderate yet selective ion coordination (Fig. 3c). The computed differential binding affinity between Li^+^ and TFSI^-^ (0.16 eV for ADH; 0.24 eV for DABA) appears compatible with efficient salt dissociation, which is consistent with PUDA’s high ionic dissociation capability and offers qualitative support for an ion-hopping conduction mechanism through alternating soft and rigid chain domains. The rigid and ordered hard-segment microdomains formed by DABA may provide a stable platform for ADH, potentially ensuring the uniform distribution and effectiveness of its coordination sites. ADH, with its hydrazide groups (–CONHNH_2_), is expected to act as a stronger Lewis base than the ether oxygen in PEG. This proposed synergy could enhance Li^+^ transport pathways along hard-segment interfaces and promote salt dissociation, thereby increasing free Li^+^ concentration.

The ionic conductivity of PUDA-*x* at 30 °C varies non-monotonically with LATP content, peaking at 1.09 × 10^−4^ S cm^−1^ for PUDA-50, as shown in Fig. 3d. This is attributed to the PUDA matrix’s strong adhesion and the interface-active role of the ionic liquid, which collectively facilitate Li^+^ exchange between phases. To directly probe the ion transfer kinetics at the organic–inorganic interface, the interfacial resistance between PUDA and a dense LATP plate was measured using a symmetric SS|PUDA|LATP|PUDA|SS cell (Supplementary Fig. 12 and Supplementary Table 3). The total resistance of the three-layer stack was deconvoluted by subtracting the separately measured resistances of the two PUDA layers and the LATP plate, yielding the interfacial resistance (*R*_int_)^32^. The Arrhenius analysis of *R*_int_^−1^ reveals an activation energy of only 0.28 eV. Such favorable interfacial kinetics, combined with the continuous percolation network, explains the enhanced ionic conductivity observed at the optimal filler loading of 50 wt%. At low filler loadings, transport is dominated by the organic phase, whereas at high loadings (e.g., PUDA-200), conductivity is limited by the agglomeration of the inorganic filler and the limited activity space of the polymer to 2.31 × 10^−6^ S cm^−1^. The ionic conductivity of pristine PUDA-0 (5.69 × 10^−6^ S cm^−1^ at 30 °C) confirms effective Li^+^ transport (Fig. 3e). This performance originates from a dual transport mechanism enabled by the microphase-separated structure: (i) segmental motion within the continuous, amorphous PEG soft-segment phase solvates and mobilizes Li^+^, while (ii) the engineered extenders (ADH and DABA), located at the interfaces between soft and hard microdomains, provide a network of dynamic, low-energy-barrier hopping sites. This synergistic design ensures efficient Li^+^ percolation while maintaining the mechanical robustness essential for dendrite suppression and interfacial stability. While the PUDA-10 and PUDA-100 layers exhibit a lower bulk ionic conductivity compared to the percolated PUDA-50 layer, their primary function within the MIGE is to ensure optimal interfacial stability at the negative electrode and positive electrode, respectively. The integrated Tri-PUDA-*x*@LATP electrolyte exhibits an effective ionic conductivity of 4.12 × 10^−5^ S cm^−1^. Crucially, the homologous polymer matrix appears to form a monolithic structure with few internal ion-migration barriers, which may help maintain sufficient Li^+^ flux across the cell. The Li^+^ transference number (*t*_Li_+) of the Tri-PUDA-*x*@LATP electrolyte reached 0.605 (Supplementary Fig. 13), significantly higher than that of conventional PEO-based systems (typically <0.3). Linear sweep voltammetry (Fig. 3f) reveals that the Tri-PUDA-*x*@LATP electrolyte achieves an expanded electrochemical stability window up to 4.9 V. This value significantly exceeds those of PEO-based polymer electrolytes (3.8 V) and PEO-LATP composites (4.2 V). The enhanced oxidative stability originates from the synergistic combination of a more robust polyurethane backbone^12^ and, dominantly, the formation of a dense, ceramic-rich protection layer at the positive electrode interface. The cross-sectional multilayer SEM image and EDS elemental mapping of Tri-PUDA-*x*@PE electrolyte (Fig. 3h and Supplementary Fig. 14) reveal a well-defined multilayer structure with a total thickness of approximately 40 μm. Mechanically, Tri-PUDA-*x*@LATP exhibits high anisotropy-resistant tensile strength, measuring 81.72 MPa in the transverse direction (TD) and 82.91 MPa in the machine direction (MD), which is over 100 times greater than that of PEO@LATP (Fig. 3g). These values are listed alongside literature data in Fig. 3i and Supplementary Table 1 for reference.

### Lithium deposition and stripping performance in Li||Li symmetric cells

**Fig. 4:**
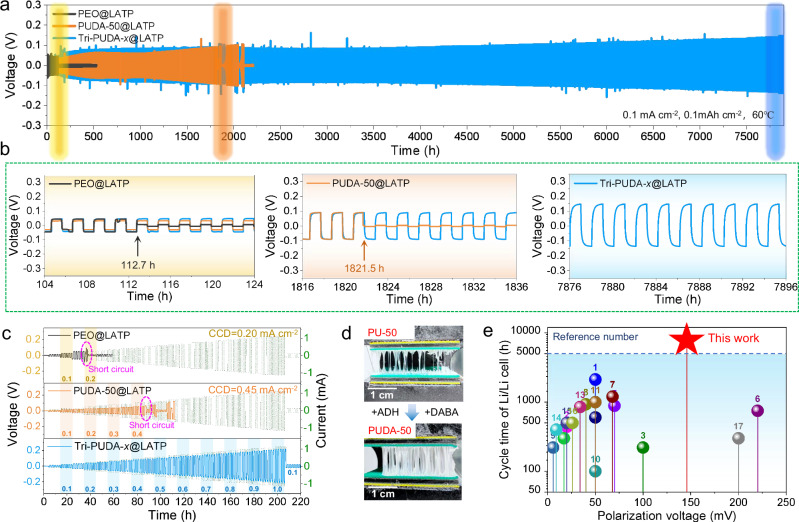
Lithium metal stability in symmetric Li | |Li cells. **a**, **b** Long-term Li plating/stripping performance of cells with PEO@LATP, PUDA-50@LATP, and Tri-PUDA-*x*@LATP at 0.1 mA cm^−2^ and 0.1 mAh cm^−2^, 60 °C. **c** Voltage profiles under stepped current densities ranging from 0.1 to 1.0 mA cm^−2^ at 60 °C. **d** Optical images comparing the adhesion and cohesion behavior of PU-50 and PUDA-50. **e** Endurance and polarization voltage of Tri-PUDA-*x*@LATP during Li plating/stripping, compared with representative polymer electrolytes from the literature. The source of the literature data shown in this figure can be found in Supplementary Information, Table 2.

The interfacial stability against lithium metal was systematically evaluated in Li||Li symmetric cells using PEO@LATP, PUDA-50@LATP, and Tri-PUDA-*x*@LATP. As shown in Fig 4a, b, the cell employing Tri-PUDA-*x*@LATP demonstrated good stability for Li plating/stripping, maintaining over 7500 h of reversible operation at 0.1 mA cm^−2^ and 0.1 mAh cm^−2^. This long-term stability stems from the gradient rigidity-flexibility coupling inherent to the MIGE architecture, where the viscoelastic PUDA matrix ensures conformal interfacial contact and effectively regulates lithium deposition behavior. In contrast, control cells with PEO@LATP and PUDA-50@LATP failed after 112.7 h and 1821.5 h, respectively, due to dendrite-induced short circuits. Further evaluation under stepped current densities (Fig. 4c) confirmed the effective dendrite-suppression capability of the gradient electrolyte. Tri-PUDA-*x*@LATP maintained a low polarization voltage of 0.1 mV at 0.5 mA cm^−2^ and sustained Li plating/stripping up to 1.0 mA cm^−2^ without failure. These values represent a 400% and 122% improvement over the critical current densities of PEO@LATP (0.2 mA cm^−2^) and PUDA-50@LATP (0.45 mA cm^−2^), respectively. Moreover, stable operation was promptly restored upon returning to 0.1 mA cm^−2^, demonstrating interfacial reversibility unattainable in conventional SPE systems. The critical role of polymer adhesion and dynamic bonding is visually illustrated in Fig. 4d, where PUDA-50 exhibits significantly improved cohesion and particle-binding capability over PU-50. This enhanced viscoelasticity not only reinforces solid–solid interfacial contact but also effectively suppresses lithium dendrite penetration by maintaining mechanical and topological integrity under high filler loading. As summarized in Fig. 4e, the Tri-PUDA-*x*@LATP electrolyte achieves a polarization voltage of 140 mV after 7500 h of continuous Li plating/stripping. Supplementary Table 2 lists corresponding values for previously reported polymer-based systems; the present result falls within the typical range, indicating potential for long-term, high-safety operation in SSLMBs.

### Electrochemical performance of solid-state lithium metal batteries

**Fig. 5:**
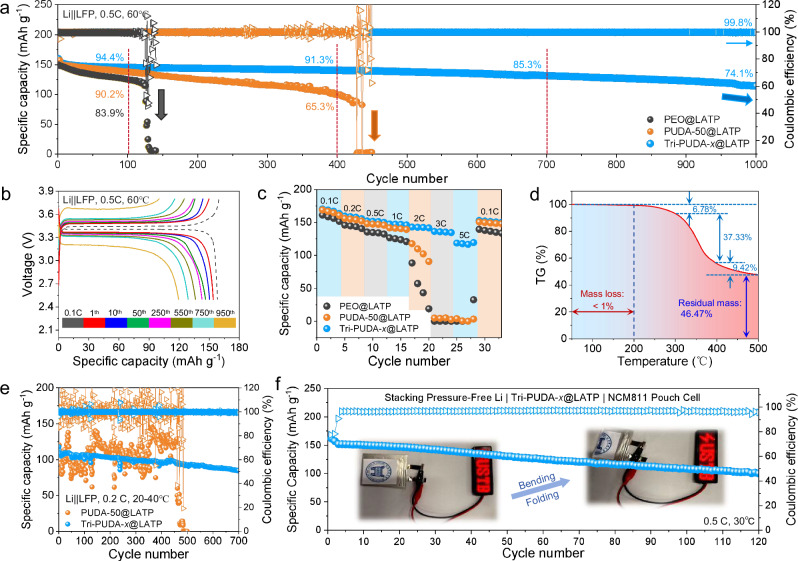
Electrochemical performance of solid-state lithium batteries. **a** Long-term cycling performance of solid-state Li||LiFePO_4_ (LFP) cells with PEO@LATP, PUDA-50@LATP, and Tri-PUDA-*x*@LATP at 0.5C, 60 °C (1C = 170 mA g^−1^). **b** Galvanostatic charge–discharge profiles of solid-state Li||LFP cells with Tri-PUDA-*x*@LATP at 0.5C, 60 °C. **c** Rate capability of solid-state Li||LFP cells with different electrolytes at 60 °C. **d** Thermogravimetric analysis (TGA) profiles from 50 to 500 °C at a heating rate of 10 K min^−1^. **e** Temperature-dependent cycling behavior of solid-state Li||LFP cells with PUDA-50@LATP and Tri-PUDA-*x*@LATP at 0.2C, 20–40 °C. **f** Cycling stability of a solid-state Li||NCM811 pouch cell with Tri-PUDA-*x*@LATP at 0.5C, 30 °C without applied stacking pressure.

The practical performance of the Tri-PUDA-*x*@LATP electrolyte was evaluated in SSLMBs, where its gradient architecture and interfacial compatibility enable good cycling stability, rate capability, and environmental tolerance. LiFePO_4_ (LFP) was selected as the primary positive electrode material for systematic evaluation due to its stable electrochemistry and minimal interfacial side reactions, allowing the mechanical and interfacial stability contributions of the MIGE to be clearly elucidated. In Li||LFP configurations at 0.5 C (1C = 170 mA g^−1^), the Tri-PUDA-*x*@LATP system achieved 94.4% capacity retention after 100 cycles, while PEO-LATP and PUDA-50@LATP showed 90.2% and 83.9%, respectively (Fig. 5a). More notably, the gradient electrolyte retained 91.3% of its initial capacity after 400 cycles, representing a 40% improvement over PUDA-50@LATP (65.3%), and continued to deliver 131.0 mAh g^−1^ (85.3% retention) after 700 cycles and 113.8 mAh g^−1^ (74.1% retention) after 1000 cycles, demonstrating long-term cycling stability. The corresponding voltage–capacity profiles (Fig. 5b) exhibited low polarization (0.117 V) and minimal capacity fade (0.026% per cycle) during 0.5 C operation. Rate capability tests further underscored the advantages of the multiscale gradient design (Fig. 5c). The Tri-PUDA-*x*@LATP cell delivered capacities of 170.3 (0.1C), 159.5 (0.2C), 152.1 (0.5C), 147.5 (1C), 142.8 (2C), 136.2 (3C), and 118.5 mAh g^−1^ (5C), with full recovery to 152.8 mAh g^−1^ upon returning to 0.1C. By contrast, cells employing PEO-LATP or PUDA-50@LATP exhibited rapid capacity decay above 2C, highlighting the essential role of continuous triphasic percolation established by the graded filler distribution in sustaining high-rate performance. Furthermore, thermogravimetric analysis (Fig. 5d) indicated minimal mass loss (<1%) below 200 °C, and dimensional stability tests (Supplementary Fig. 15) revealed minor thermal shrinkage at 120 °C, which is lower than that of commercial PE separators widely used in liquid batteries. Under milder conditions (0.1 C, 30°C), the system maintained 124.9 mAh g^−1^ after 1700 cycles (89.3% retention), whereas PEO-LATP cells failed under identical testing (Supplementary Fig. 16). The gradual capacity increase during the initial ~200 cycles at 30 °C reflects progressive interfacial optimization enabled by the viscoelastic PUDA matrix. The occasional exceedance of 100 % Coulombic efficiency is attributed to the reversible recovery of previously isolated lithium, facilitated by the continuous interfacial improvement and dynamic self-healing of the PUDA framework, a phenomenon consistent with prior reports on adaptive solid-state interfaces^33,34^.

The MIGE design also demonstrated notable compatibility with high-volume-change silicon-carbon (SiC) composite electrodes. In Li||SiC half-cells, Tri-PUDA-*x*@LATP enabled an initial discharge capacity of 519.6 mAh g^−1^ at 0.1C with a capacity retention of 75.4% after 140 cycles, while PUDA-50@LATP showed poor reversibility (Supplementary Fig. 17). This performance highlights the material’s ability to accommodate large volume variations through its viscoelastic self-healing characteristics. Furthermore, the gradient electrolyte exhibited good environmental adaptability, maintaining stable operation across a wide temperature range (20 °C to 40 °C) with minimal capacity fluctuation and high Coulombic efficiency (Fig. 5e). When paired with a high-voltage LiNi_0.8_Co_0.1_Mn_0.1_O_2_ (NCM811) positive electrode (1C = 220 mA g^−1^), the solid-state Li|Tri-PUDA-*x*@LATP | NCM811 cell achieved an initial discharge capacity of 179.4 mAh g^−1^ and maintains an average Coulombic efficiency of 96.5% over 200 cycles at 0.5 C, while the cell with the PUDA-50@LATP electrolyte showed reduced electrochemical performance (Supplementary Fig. 18). These results demonstrate the robust cycling stability of the MIGE-based cell even under near-ambient operating conditions. More importantly, the corresponding pouch cell configuration without external stacking pressure delivered a high initial discharge capacity of 152.0 mAh g^−1^ at 0.5 C with an average Coulombic efficiency of 96.7%, and maintained safe operation even after bending and folding (Fig. 5f), confirming the high mechanical robustness and interfacial integrity of the MIGE system. This work demonstrates that high-voltage stability in polymer-based composites can be achieved through a mechano-integrated design that controls interfacial architecture.

### Industrial-scale manufacturing and practical viability of MIGE

**Fig. 6:**
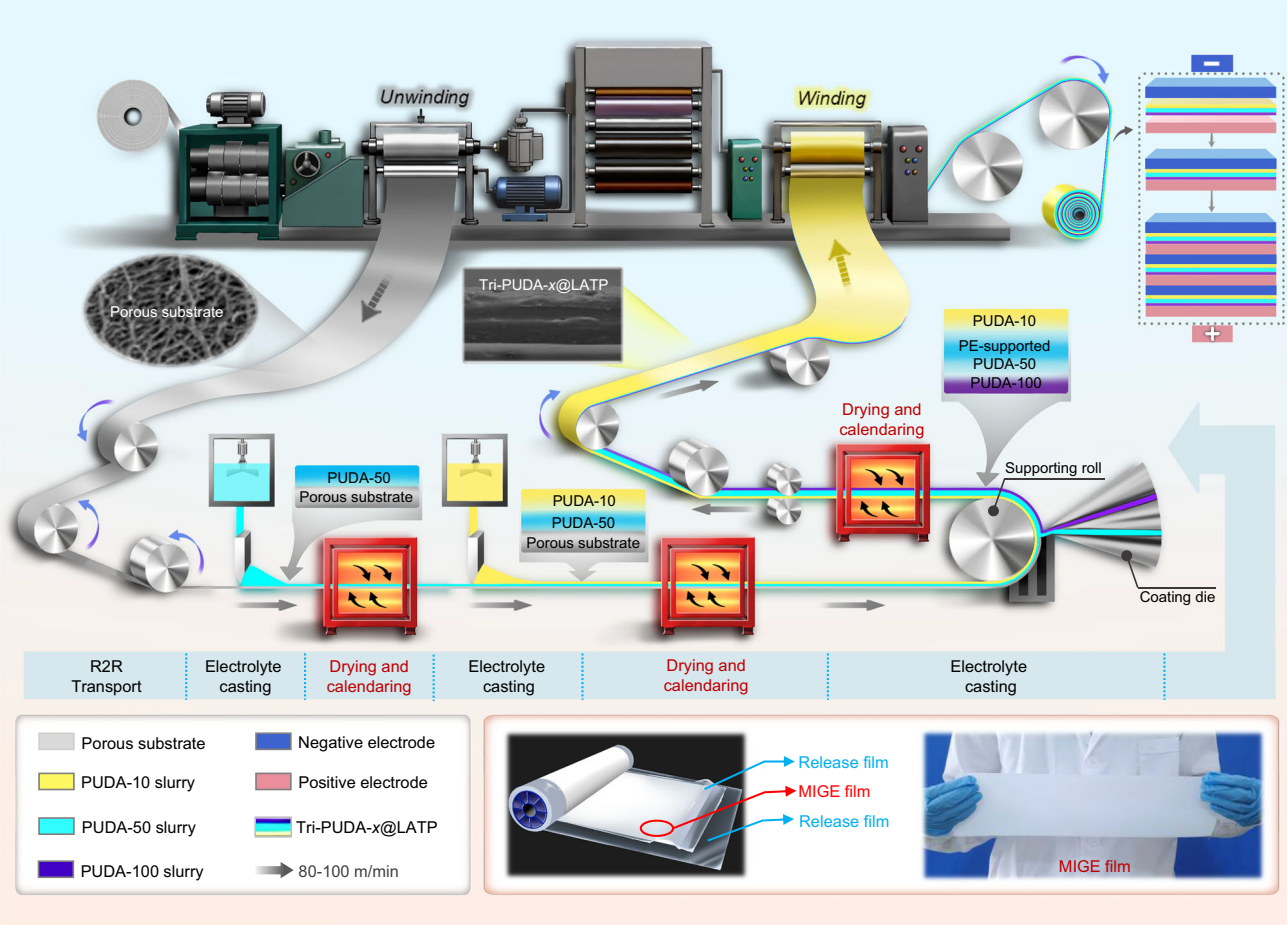
Roll-to-roll manufacturing process for multilayer coating and transfer printing of the MIGE. The process includes unwinding of a porous polyethylene (PE) separator; front-side coating of PUDA-50 followed by drying and hot-pressing; additional coating of PUDA-10 on the same side; simultaneous extrusion coating of PUDA-50 and PUDA-100 on the reverse side; calendering for thickness control and rewinding; and transfer printing of the MIGE onto positive and negative electrodes.

The transition from laboratory-scale fabrication to industrially viable production represents a critical challenge for solid-state batteries. The proposed MIGE design demonstrates compelling potential for high-throughput roll-to-roll (R2R) manufacturing, a fundamental advantage over conventional solid electrolytes that typically rely on batch processing or lamination techniques. As schematically illustrated in Fig. 6, our multilayer coating process enables continuous production of functionally graded electrolyte membranes with precise control over layer composition and interfacial integrity. The process begins with unwinding a porous PE substrate (12 μm), which serves as both a mechanical scaffold and a processing aid, followed by sequential slot-die coating of the functional layers: first the ion-transport layer (PUDA-50) and the negative electrode interface (PUDA-10) on the front side, each followed by low-temperature drying (60–80 °C) and mild hot-pressing. The positive electrode-facing layer (PUDA-100) is then simultaneously coated on the reverse side alongside a second PUDA-50 layer, forming a dense oxygen barrier and a symmetric ion-conduction network. Final calendering controls the precise membrane thickness to ≤50 μm while enhancing interlayer adhesion and surface uniformity. Cross-sectional SEM imaging confirms seamless integration between MIGE layers. To facilitate handling, the tacky MIGE surface is protected with release liners, which are removed prior to cell assembly via a solvent-free transfer printing technique that directly laminates the electrolyte with positive electrode and lithium negative electrode layers under minimal pressure. This eliminates the common challenges of solid-solid contact loss and interfacial degradation during cell assembly.

This integrated manufacturing route offers several decisive advantages over conventional solid electrolyte processing. First, the R2R line achieves production speeds of 80–100 m min^−1^. In comparison, batch-based casting or multilayer lamination processes operate at lower throughput. The theoretical production capacity can reach more than 1 million m² annually per standard coating line. Second, the low drying temperature (60–80 °C) minimizes energy consumption and enables compatibility with heat-sensitive components. Third, the high mechanical strength (>80 MPa) and viscoelasticity (>5000% strain) of the PUDA matrix allow the MIGE to maintain structural integrity throughout the high-speed coating, drying, and winding stages and ensure dimensional stability throughout electrode volume changes, addressing a common failure mode in brittle ceramic or low-strength polymer electrolytes. Fourth, the entire process operates under ambient conditions, avoiding the energy-intensive dry rooms required for sulfide or halide electrolytes. Most importantly, the MIGE’s graded architecture and dynamic self-healing capability enable stable battery operation without external stack pressure, eliminating the need for heavy cell fixtures and simplifying module integration.

From a cost perspective, the solution processability of PUDA-based electrolytes significantly reduces material waste and energy consumption compared to vapor deposition or hot-pressing methods. The ability to utilize conventional coating equipment without major modification lowers capital investment barriers for existing battery manufacturers. Furthermore, the high filler-loading capacity (>200 wt%) reduces the relative cost of the polymer matrix while maximizing the utilization of high-performance ceramic ion conductors. This combination of high-speed manufacturing, good electrochemical performance, and mechanical robustness positions MIGE as a scalable platform for next-generation SSLMBs. By bridging the gap between molecular design and industrial processing, the MIGE platform represents a scalable, high-performance, and commercially promising pathway toward solid-state lithium metal batteries.

## Discussion

In this work, we have demonstrated a multi-scale design approach for solid electrolytes through the development of a Mechano-Integrated Gradient Electrolyte (MIGE). This approach systematically integrates molecular engineering, mesoscale architecture, and macroscopic functionality to overcome long-standing limitations in conventional solid electrolyte design. The incorporation of dual hydrogen-bonding extenders, DABA and ADH, enables a dynamic yet robust supramolecular network that delivers high viscoelasticity (>5000% elongation), autonomous self-healing, and high tensile strength (>80 MPa), while enabling inorganic filler loadings of more than 200 wt%. The MIGE architecture represents a fundamental advance beyond homogeneous composite. By spatially grading the LATP content, we decouple the interfacial priorities: conformal, strain‑tolerant contact at the negative electrode and a dense, oxidation‑resistant yet still conformal interface at the positive electrode. Multi-scale theoretical modeling reveals a critical percolation threshold and clarifies the roles of polymer segmental interaction and ion coordination in facilitating efficient Li^+^ transport. The resulting MIGE system achieves improved electrochemical performance: extended cycling stability in Li | |LFP cells (74.1% capacity retention after 1000 cycles at 0.5 C, 89.3% capacity retention after 1700 cycles at 0.1 C), high-rate capability (up to 5 C), stable environmental resilience (20–40 °C), and prolonged dendrite-free Li plating/stripping (>7500 h). Notably, the corresponding stack-pressure-free NCM811 pouch cell delivers a high initial capacity of 152.0 mAh g^−1^ at 0.5 C with an average Coulombic efficiency of 96.7%, and maintained safe operation even under bending and folding.

Crucially, the MIGE represents a mechanically integrated system, as opposed to a mechanically compromised composite or an assembled laminate. Mechanical functionality is not merely appended but is inherently encoded from the molecular level upward into the electrolyte’s morphology and chemistry. This integration enables a single membrane to meet spatially conflicting interfacial demands. Furthermore, the MIGE platform presents a generalizable design framework. While LATP serves as an effective model filler herein, the viscoelastic PUDA matrix can host a variety of inorganic fillers. Their composition can be spatially tailored within the gradient, for instance, employing negative electrode-stable garnets (e.g., LLZO) at the Li interface while retaining high-conductivity, oxidation-resistant fillers like LATP at the positive electrode to precisely address interfacial chemical requirements, offering a highly adaptable materials platform for diverse SSLMB chemistries. While this work demonstrates a high-performance electrolyte platform that operates without external stack pressure, we note that the realization of practical high-energy-density SSLMBs will also require advances in composite-electrode design, particularly for thick electrodes. Achieving uniform electrolyte distribution and maintaining durable ionic/electronic contact throughout such electrodes remains an important challenges. Future work will focus on integrating the MIGE with advanced electrode architectures (e.g., using in-situ polymerization or gradient electrode composites) to simultaneously address electrolyte and electrode microstructural requirements.

## Methods

### Materials

Poly(ethylene glycol) (PEG, 2000 g mol^−1^, 99%), methylene diphenyl diisocyanate (MDI, 98%), 4,4’-diaminobenzanilide (DABA, 99%), adipic dihydrazide (ADH, 98%), dibutyltin dilaurate (DBTDL, 95%), lithium bis(trifluoromethanesulfonyl)imide (LiTFSI, 99.9%), 1-ethyl-3-methylimidazolium bis(trifluoromethylsulfonyl)imide ([EMIM][TFSI], 99%), and anhydrous N,N-dimethylformamide (DMF, 99.8%) were purchased from Innochem. Li_1.3_Al_0.3_Ti_1.7_(PO_4_)_3_ (LATP, average particle size 100 nm, Tianmu Pioneer Battery Material Technology Co., Ltd) powder was used as received. Porous polyethylene (PE) separator (porosity 70%, thickness 12 μm, Yunnan Energy New Material Co.,Ltd.) was used as the scaffold. Super P carbon black (MTI Corporation), poly(vinylidene fluoride) (PVDF, Solef 5130, Solvay), N-methyl-2-pyrrolidone (NMP, 99.5%, Sigma-Aldrich), and aluminum foil (99.9%, 15 μm thickness, MTI Corporation) and copper foil (99.8%, 10 μm thickness, MTI Corporation) were used for electrode fabrication. Lithium metal foil (99.9%, 500 μm or 40 μm thickness, China Energy Lithium Co.) was used as the negative electrode.

### Synthesis of PUDA-*x* and Tri-PUDA-*x*@LATP electrolytes

The polyurethane-based matrix (PUDA) was prepared by a polyaddition reaction. In a typical procedure, 2.00 g of PEG was dehydrated in an open vial at 100 °C for 2 h, then cooled to 80 °C. Anhydrous DMF was added under magnetic stirring (400 rpm) until complete dissolution. MDI (molar ratio PEG:MDI = 1:2) and 30 μL of DBTDL were introduced, and the mixture was stirred at 80 °C for 2 h under N_2_ to form the PU prepolymer. The solution was cooled to 40 °C, and a DMF solution containing DABA and ADH (molar ratio PEG:DABA:ADH = 2:1:1) was added dropwise. The reaction was continued for 4 h under vigorous stirring, yielding a highly viscous PUDA solution. For composite electrolytes PUDA-*x*, LATP powder was pre-dispersed in anhydrous DMF by ultrasonication for 30 min and then mixed into the PUDA solution. LiTFSI and [EMIM][TFSI] were added at a mass ratio of PUDA:[EMIM][TFSI]:LiTFSI = 10:1:1, and the mixture was stirred until homogeneous. The resulting slurry was cast onto release paper using the doctor blade method and dried under vacuum at 80 °C for 24 h to obtain freestanding films. The films were stored in an argon-filled glovebox (O_2_ < 0.1 ppm, H_2_O < 0.1 ppm). The MIGE was fabricated by a sequential slot-die coating process (custom-built). A porous PE substrate (width 200 mm) was unwound. The first pass coated PUDA-50 (50 wt% LATP) on one side, followed by drying at 70 ± 5 °C in a forced-air oven and mild calendaring (0.5 MPa, 60 °C). The second pass coated PUDA-10 (10 wt% LATP) on top of the PUDA-50 layer. The web was then flipped, followed by double-side coating of the PUDA-50 and PUDA-100 (100 wt% LATP) on the reverse side. After final drying at 80 °C under vacuum and calendaring, the total electrolyte thickness was controlled to approximately 40 μm. Densification and uniform thickness control were achieved by hot rolling at 60 °C under a pressure of 5 MPa. The MIGE film was wound with protective release liners and stored in a dry room (dew point −40 °C).

### Characterization methods

XRD was performed on a Bruker D8 Advance diffractometer with Cu Kα radiation (*λ* = 1.5405 Å) generated at 40 kV, 40 mA. Data were collected in the 2θ range of 5°−90° with a step size of 0.01° and a counting time of 0.5 s per step. XPS was conducted on a Thermo Scientific ESCALAB 250 Xi spectrometer using a monochromatic Al Kα X-ray source (150 W). The samples were prepared in a glovebox filled with argon and transferred to the XPS chamber in a sealed container filled with argon to avoid air exposure. Raman spectroscopy was performed on a HORIBA LabRAM HR Evolution system with a 532 nm laser source. Spectra were acquired in the range of 400–4000 cm^−1^ with a resolution of 2 cm^−1^. SEM and energy-dispersive X-ray spectroscopy (EDS) were performed on a Hitachi S-8400 field-emission SEM at an accelerating voltage of 10 kV. Cross-sectional samples were prepared by cryo-fracturing in liquid nitrogen and sputter-coated with gold prior to imaging. Ulasonic imaging in pulse-echo mode was performed using a Feli 1000 system equipped with a 4 MHz center-frequency transducer and a 100 V excitation voltage. The time-of-flight and signal amplitude of reflected waves were analyzed to evaluate the density and structural homogeneity of the electrolyte pellets. Thermal shrinkage tests were performed by placing electrolyte films in a temperature-controlled oven (accuracy ±1 °C) and maintaining isothermal conditions at set temperatures (e.g., 90 °C, 120 °C) for 1 h. Tensile properties were measured using a universal testing machine (MT4103D, Shenzhen SANS) with a 100 N load cell. The rectangular specimens (gauge length 20 mm, width 10 mm) were stretched at a crosshead speed of 20 mm min^−1^ under ambient conditions. Thermogravimetric analysis (TGA) was conducted on a NETZSCH STA 449F5 instrument under a nitrogen atmosphere. Samples were heated from 40 °C to 550 °C at a ramp rate of 10 K·min^−1^.

### Electrochemical measurements

Ionic conductivity was measured using stainless steel (SS) blocking electrodes (diameter 16 mm) in CR2032 coin cells. Electrochemical impedance spectroscopy (EIS) was performed on an Autolab electrochemical workstation (Metrohm) in potentiostatic mode with an AC amplitude of 10 mV and a frequency range of 1000 kHz to 1 Hz (10 points per decade). Prior to each measurement, the open-circuit voltage was monitored until stable (variation <1 mV over 5 min), and the cell was then equilibrated at the target temperature for 20 min in a thermostatic chamber (accuracy ±0.5 °C). Temperature-dependent tests were conducted from 30 °C to 70 °C in steps of 10 °C. The ionic conductivity σ was calculated by *σ* = *L*/(*R*·*A*), where *L* is the film thickness (measured by a digital micrometer), *R* is the bulk resistance (obtained from the high-frequency intercept of the Nyquist plot), and *A* is the electrode area. Interfacial resistance between PUDA and LATP was evaluated using a three-layer laminate SS|PUDA|LATP|PUDA|SS. Dense LATP plates (diameter 16 mm, thickness ≈0.95 mm) were prepared by pressing LATP powder into a pellet, sintering at 900 °C for 4 h, polishing both surfaces, and sputter-coating with Au (80 °C, 1 h). PUDA films (diameter 16.2 mm, thickness ≈ 150 μm) were cast separately. EIS was performed under the same conditions as above. The total resistance *R*_total_ was obtained from the Nyquist plot. The resistances of a single PUDA film (*R*_PUDA_) and the LATP plate (*R*_LATP_) were measured separately. The total interfacial resistance *R*_int,total_ =  *R*_total_ − 2*R*_PUDA_ − *R*_LATP_, and the single-interface resistance *R*_int_ = *R*_int,total_/2. The Li⁺ transference number (*t*_Li⁺_) was determined using the Bruce–Vincent method. A symmetric Li|Tri-PUDA-*x*@LATP|Li cell was polarized at a constant potential of 10 mV for 7000 s, and the current evolution was recorded. Initial and steady-state currents (*I*_0_, *I*_s_) and interfacial resistances (*R*_0_, *R*_s_) were used to calculate *t*_Li⁺_ = *I*_s_ (ΔV - *I*_0_*R*_0_)/[*I*_0_(ΔV - *I*_s_*R*_s_)]. LSV was performed on Li|electrolyte|SS asymmetric cells under ambient conditions with a scan rate of 1 mV s^−1^ from open-circuit voltage to 7 V (vs. Li^+^/Li). Prior to measurement, cells were heat-treated at 80 °C for 1 h to ensure interfacial contact.

### Cell assembly and electrochemical cycling

All cell assemblies were performed in an argon-filled glovebox (O_2_ < 0.1 ppm, H_2_O < 0.1 ppm). Lithium metal electrodes were prepared from Li foil (500 μm thickness for coin cells, 40 μm thickness for pouch cells) by punching discs of 12 mm or 16 mm diameter. The native oxide layer was removed by gently scraping both sides with a clean polypropylene scraper; no chemical cleaning was used. The Li discs were used immediately after preparation without storage. Composite positive electrodes were prepared by mixing LFP or NCM811, Super P, PVDF, and LATP powder at a mass ratio of 7:1:1:1 in NMP. The slurry was stirred for 12 h, coated onto aluminum foil (15 μm thickness, 99.9% purity) using a doctor blade, dried at 120 °C for 8 h under vacuum, and punched into discs of 12 mm diameter. The typical active mass loading was 3–4 mg cm^−2^. SiC (*D*_50_ = 13 μm, specific surface area 1.51 m^2^ g^−1^, initial Coulombic efficiency 92%, reversible capacity ≈600 mAh g^−1^) was mixed with Super P, PVDF, and PUDA at a weight ratio of 7:1:1:1 in NMP. The slurry was coated onto copper foil (10 μm thickness, 99.8% purity) using a doctor blade, dried at 80 °C for 8 h under vacuum, and punched into discs of 14 mm diameter. The active material mass loading was 11–12 mg cm^−2^. CR2032 coin cells were assembled by stacking the MIGE electrolyte disc (16.2 mm diameter) between the positive electrode (or SS blocking electrode) and the Li negative electrode (or Li counter electrode). No liquid electrolyte was added, Pouch cells (5 cm × 7 cm) were assembled using Li foil (40 μm thickness) and NCM811 positive electrodes, sealed under vacuum, and cycled without external stack pressure. Galvanostatic charge–discharge tests were performed using NEWARE battery test system (Shenzhen, China) and LAND CT2001A battery test system (Wuhan, China). All cycling tests were performed at 30 ± 1 °C in a thermostatic chamber, unless otherwise specified. For elevated temperature tests (60 °C), the chamber temperature was maintained at 60 ± 0.5 °C.

### Theoretical calculations

COMSOL simulations were performed using the PDE module of COMSOL Multiphysics 6.0. A two‑dimensional rectangular domain of 1000 × 1000 μm^2^ with periodic boundary conditions was constructed. The left boundary was assigned a normalized Li⁺ concentration of 1 and an electric potential of 0 V; the right boundary was set to concentration of 0 and a potential of −0.1 V. A phase‑field model was employed to distinguish the polymer phase (*ζ*) and the ceramic filler phase (*η*). The dimensionless diffusion coefficients for the polymer electrolyte (*D*_*p*_) and the ceramic electrolyte (*D*_*c*_) were set to 0.05 and 0.5, respectively. The effective diffusion coefficient and electrical conductivity were interpolated using the two phase-field variables. Details of the equations are provided in the Supplementary Information.

*MD simulations* were conducted using the Forcite module in Materials Studio 2020. with the Polymer Consistent Force Field. A cubic simulation cell of 50 Å × 50 Å × 50 Å with periodic boundaries were constructed. The molecular structures of PEG, PU, PUA, PUD, and PUDA are shown in Supplementary Data 4–8. The molar ratio of [‑C‑C‑O‑] repeating units to Li⁺ was set to 18:1. All initial configurations underwent full geometry optimization to remove unrealistic atomic overlaps. Subsequently, an equilibration run was carried out in the isothermal‑isobaric (NPT) ensemble for 500 ps at 298 K and 1 atm, followed by a production run in the canonical (NVT) ensemble for 2 ns with a time step of 1 fs. Temperature was controlled by the Nosé–Hoover thermostat, pressure was controlled by the Berendsen barostat. The equations of motion were integrated using the steepest descent algorithm with convergence criteria: energy tolerance <1.0 × 10^−4^ kcal mol^−1^, maximum atomic displacement <5.0 × 10^−5^ Å, and force tolerance <5.0 × 10^−3 ^kcal mol^−1^ Å^−1^. It should be noted that MD simulations rely on classical force fields that cannot fully capture quantum mechanical effects, and the simulation time scale (nanoseconds) is much shorter than the actual dynamic processes in polymer electrolytes. Therefore, the calculated binding energies and diffusion coefficients should be interpreted as qualitative trends rather than quantitative predictions.

*DFT calculations* were performed with the DMol3 module. The Perdew–Burke–Ernzerhof (PBE) generalized gradient approximation (GGA) was employed. A double numerical basis set with polarization functions (DNP) was used. The atomic structures of ADH, DABA, and MDI are shown in Supplementary Data 1–3. All structures were fully relaxed without symmetry constraints. Convergence thresholds were set as follows: energy change <1.0 × 10^−5^ Ha, maximum force <2.0 × 10^−3^ Ha Å^−1^, and maximum displacement <5.0 × 10^−3^ Å. Van der Waals interactions were corrected using Grimme’s DFT-D3 method. Binding energies were calculated as Δ*E* = *E*_total_ – *E*_Li⁺_ – *E*_polymer_, where *E*_total_ is the total energy of the system with adsorbed Li^+^, *E*_Li⁺_ is the energy of Li^+^, and *E*_polymer_ is the energy of the corresponding polymer. We note that DFT calculations are based on idealized molecular models in the gas phase (0 K) and do not account for the dynamic, time‑dependent behavior, thermal fluctuations, or the complex solvation environment present under real operating conditions. Consequently, the computed binding energies should be regarded as approximate qualitative indicators rather than absolute values.

## Supplementary information

Supplementary Information

Description of Additional Supplementary Files

Supplementary Data 1-8

Transparent Peer Review file

## Source data

Source Data

## Data Availability

All data generated in this study are provided in the Source Data File. Source data are provided with this paper.
