# Supplementary material for: A mechano-integrated gradient electrolyte for long-cycling solid-state lithium metal batteries
Source: Nat Commun. 2026 Jun 24;17:8277. doi: 10.1038/s41467-026-74573-0 (PMC13470014; doi:10.1038/s41467-026-74573-0)
Supplement: Supplementary file 1 [file 41467_2026_74573_MOESM1_ESM.pdf]

Supplementary Information

A mechano-integrated gradient electrolyte for long-cycling solid-state lithium metal batteries

Xiaoping Yi,[1,2] Guoqing Qi,[1] Wending Pan,[3,4]* Kaishan Xiao,[5] Yixin Yang,[6] Yang Yang,[7] Bitong Wang,[5] Xiaolong Zhao,[4] Xunliang Liu,[1,]* and Hong Li[5,]*

[1] School of Energy and Environmental Engineering, University of Science and Technology Beijing, Beijing 100083, China

[2] Taihang Institute of Advanced Energy Storage Technology, Baoding, Hebei, 071000, China

[3] School of Energy and Power Engineering, Dalian University of Technology, No. 2 Linggong Road, Ganjingzi District, Dalian City, Liaoning Province, 116024, China

[4] Department of Mechanical Engineering, The University of Hong Kong, Hong Kong, 999077, China

[5] Beijing Key Laboratory for New Energy Materials and Devices, Institute of Physics, Chinese Academy of Sciences, Beijing, 100190, China

[6] College of Materials Science and Optoelectronic Technology, University of Chinese Academy of Sciences, Beijing, 100049, China

[7] Zhejiang Key Laboratory of All-Solid-State Battery, Ningbo Key Laboratory of All-Solid-State Battery, Ningbo, Zhejiang, 315200, China

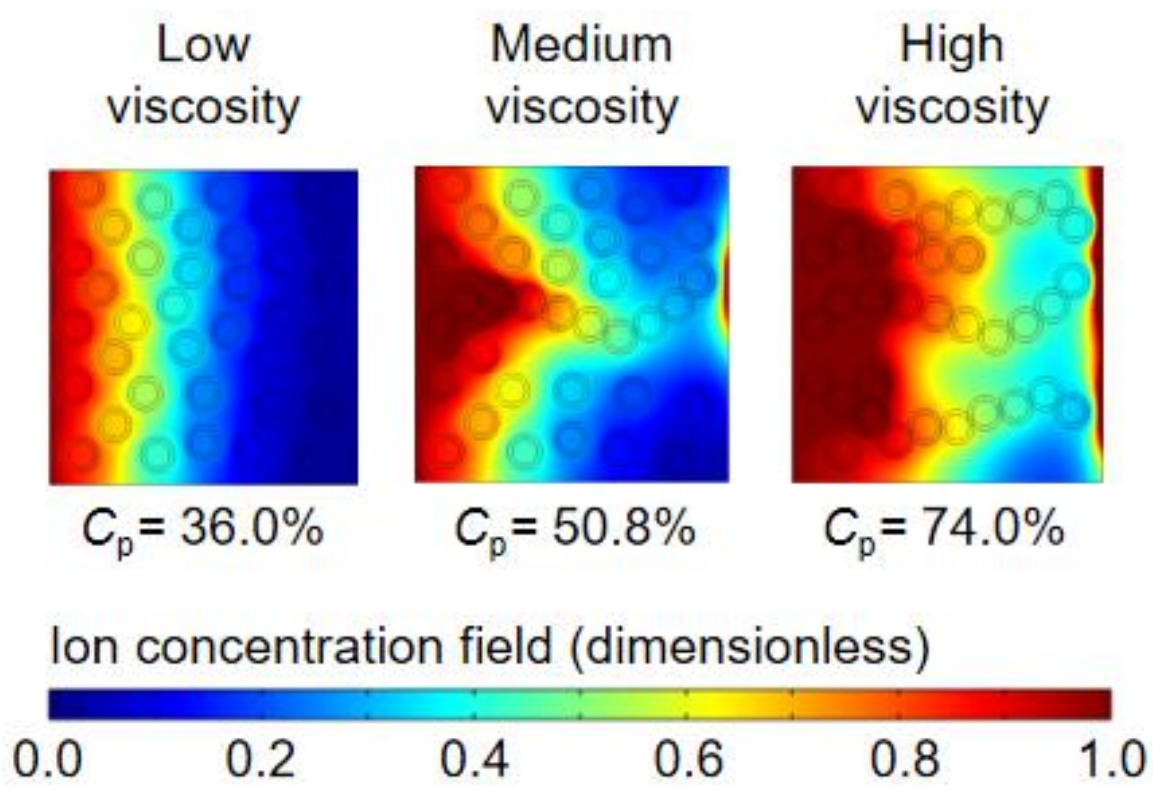

Supplementary Fig. 1 Ion-transport efficiency among homogeneous, single-percolation, and multichannel-percolation architectures at equivalent filler content under $\sigma_{polymer}$.

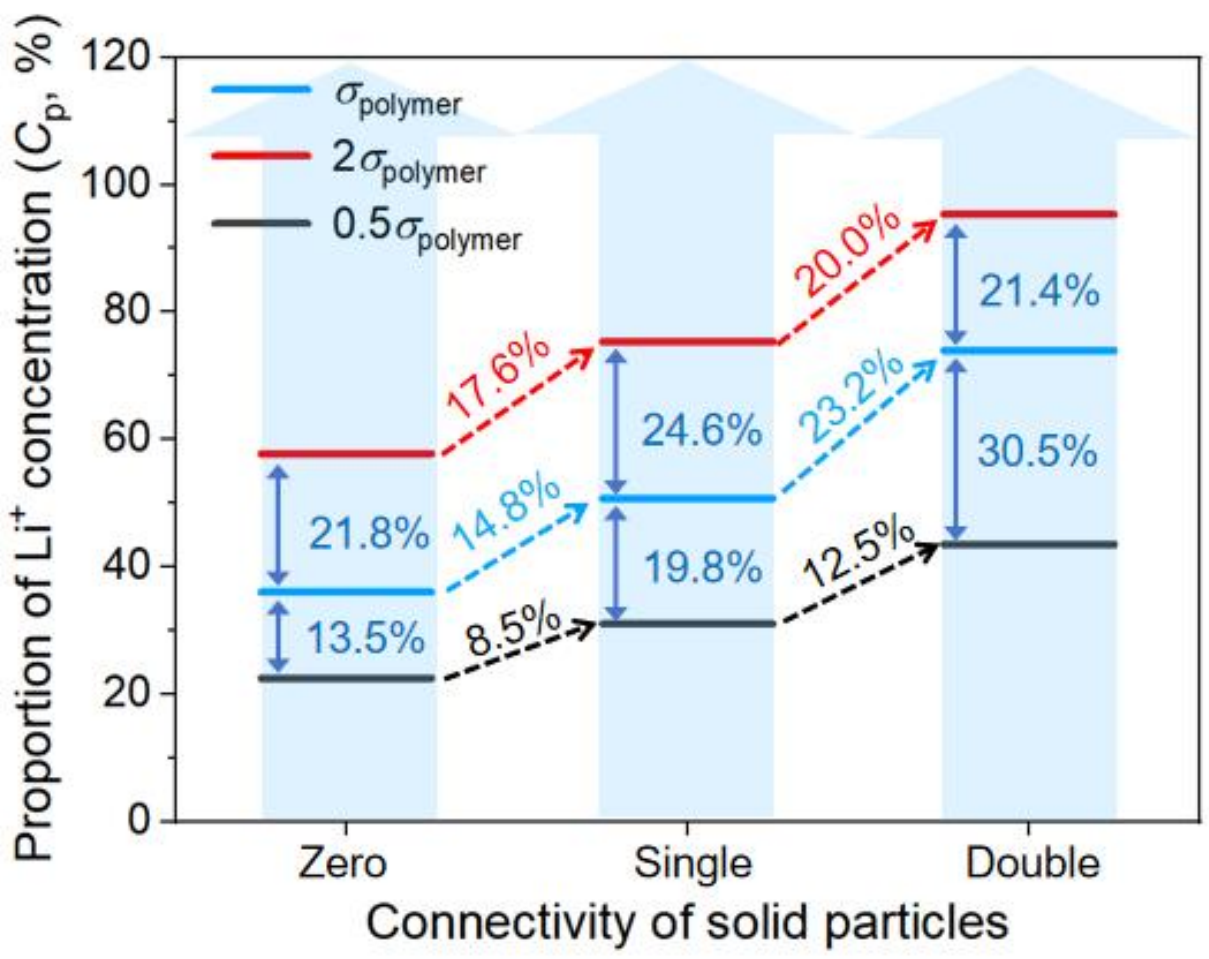

Supplementary Fig. 2 Correlation between polymer ionic conductivity and topological connectivity of ion percolation channels on Li^+ flux enhancement (S_p=50.8%).

For COMSOL calculations, the model was constructed based on the following assumptions: (1) The solid electrolyte materials remain structurally intact without deformation or cracking; (2) The simulation focuses exclusively on ion transport within the composite solid electrolyte, neglecting the effects of lithium deposition and stripping at the electrode interfaces; (3) Lithium ion supply is considered sufficient; (4) Only the transport of lithium ions is considered, while the influence of anions is neglected.

In the electrodeposition system, the driving forces for lithium ion migration are the concentration gradient and the electric potential gradient. The ion flux can be expressed as:

$$J_i = -D_i\left(\nabla c_i + c_i \nabla \frac{F\varphi}{RT}\right) \quad (1)$$

Where $c_i = \{c_a, c_+, c_-\}$ denote the concentrations of lithium atoms, lithium ions, and

anions, respectively; φ is the electric potential; F is the Faraday constant (96485 C mol^{-1}); R is the universal gas constant (8.314 J $(mol{\cdot}K)^{-1}$); and T is the temperature. By neglecting the diffusion of lithium atoms and anions, and introducing the normalized concentration $\left\{C_{\mathrm{a}}=\frac{c_{\mathrm{a}}}{c_{\mathrm{s}}}, C_{+}=\frac{c_{+}}{c_{0}}, C_{-}=\frac{c_{-}}{c_{0}}\right\}$, the expression simplifies to:

$$\frac{\partial C_{+}}{\partial t}=\nabla\cdot\left[D_{\mathrm{eff}}\nabla C_{+}+\frac{D_{\mathrm{eff}}C_{+}}{RT}nF\nabla\varphi\right] \tag{2}$$

Here, D_{eff} is the effective diffusion coefficient; c_{s} is the molar density of lithium; and c_0 is the volume concentration of the electrolyte.

The effective diffusion coefficient is defined via an interpolation function involving $h(\zeta)=\zeta^3\left(10-15\zeta+\zeta^2\right)$ and $h(\eta)=\eta^3\left(10-15\eta+\eta^2\right)$ as:

$$D_{\mathrm{eff}}=D_{\mathrm{p}}h(\zeta)+\left(D_{\mathrm{c}}h(\eta)+D_{\mathrm{f}}\left(1-h(\eta)\right)\right)(1-h(\zeta)) \tag{3}$$

Where D_{p} and D_{c} represent the diffusion coefficients of lithium ions in the polymer and ceramic solid electrolytes, respectively, and D_{f} denotes the diffusion coefficient at the two-phase interface.

Assuming the system is electrically neutral, charge conservation is governed by the Poisson equation:

$$\nabla\cdot\sigma_{\mathrm{eff}}\nabla\varphi(r,t)=0 \tag{4}$$

Analogous to the effective diffusion coefficient, the effective electrical conductivity is expressed as:

$$\sigma_{\mathrm{eff}}=\sigma_{\mathrm{p}}h(\zeta)+\left(\sigma_{\mathrm{c}}h(\eta)+\sigma_{\mathrm{f}}\left(1-h(\eta)\right)\right)(1-h(\zeta)) \tag{5}$$

where σ_{p} and σ_{c} are the conductivities of the polymer and ceramic solid electrolytes, respectively, and σ_{f} represents the interfacial conductivity between the two phases.

The normalized Li^+ concentration distribution, C_p:

$$C_{\mathrm{p}} = \frac{\iint c d\Omega}{\Omega} \times 100\% \tag{6}$$

The area fraction occupied by filler particles and organic-inorganic interphases, M_p:

$$M_{\mathrm{p}} = \frac{\iint m d\Omega}{\Omega} \times 100\% \tag{7}$$

The filler-to-polymer area ratio, S_p:

$$S_{\mathrm{p}} = \frac{M_{\mathrm{p}}}{1 - M_{\mathrm{p}}} \times 100\% \tag{8}$$

where c is the dimensionless Li^+ concentration field, m is the dimensionless field representing the filler and interphase domains, and Ω is the total area of the computational domain. The parameter S_p is qualitatively correlated with the experimental mass loading (wt%) of LATP filler, as both reflect the relative abundance of the inorganic phase within the composite.

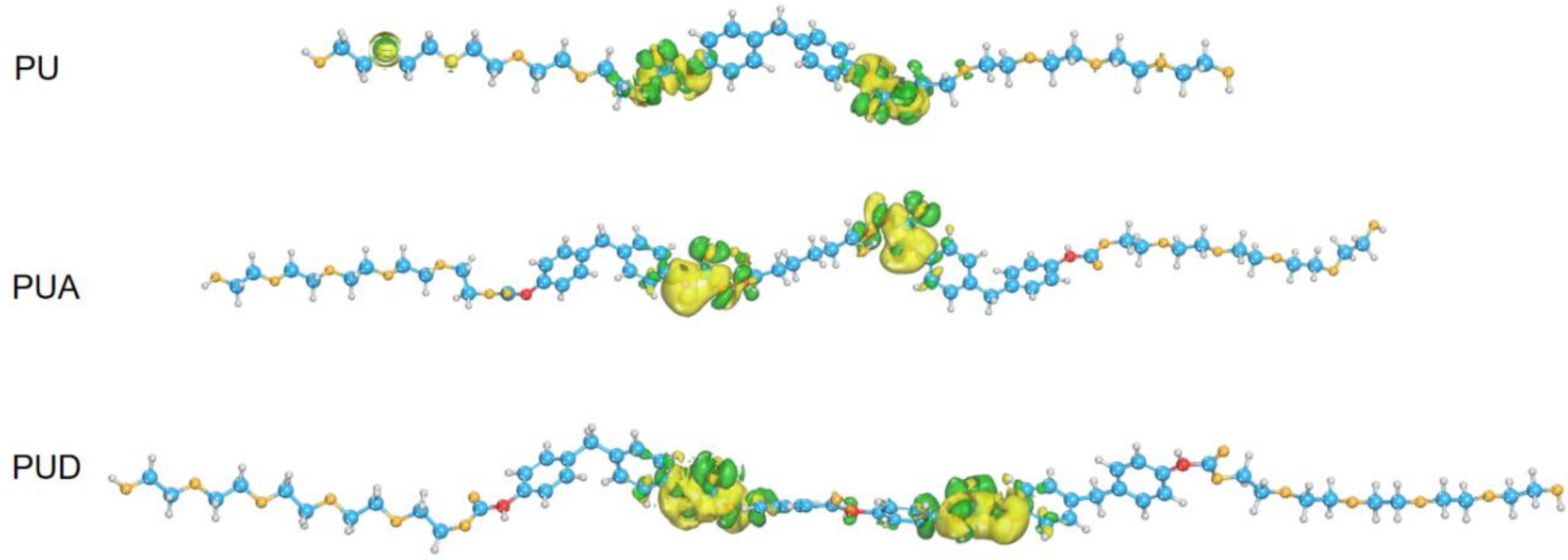

Supplementary Fig. 3 Charge density difference of PU, PUA, and PUD. Different colored spheres represent atoms: blue, C; red, N; yellow, O; gray, H. Green and yellow regions denote electron accumulation and depletion, respectively.

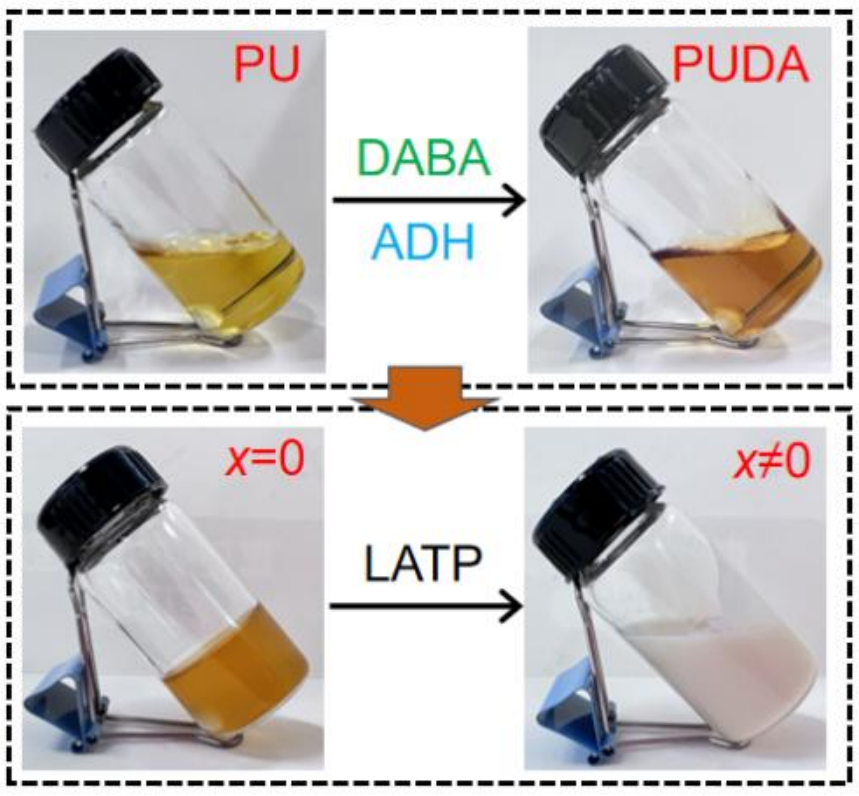

Supplementary Fig. 4 Optical images of synthesized PUDA and PUDA-*x* precursors.

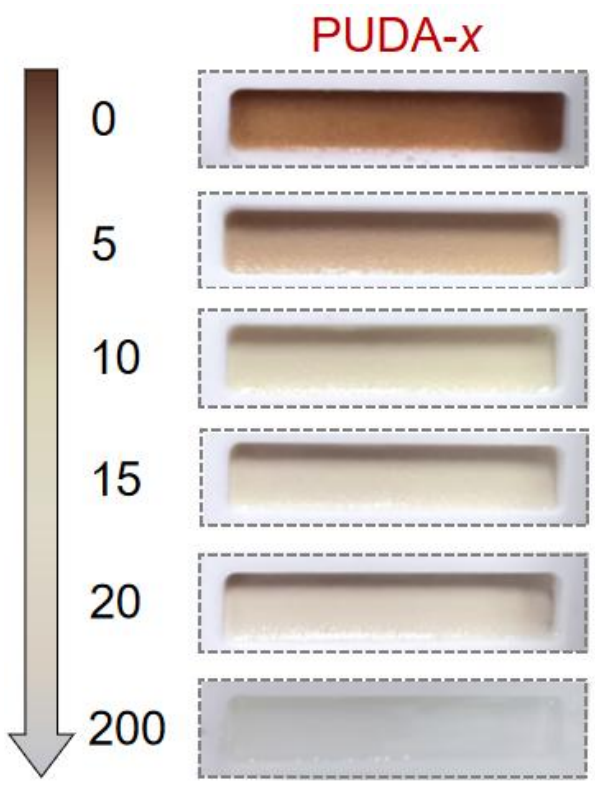

Supplementary Fig. 5 Photographs of PUDA-*x* solid electrolytes (*x* = LATP wt%).

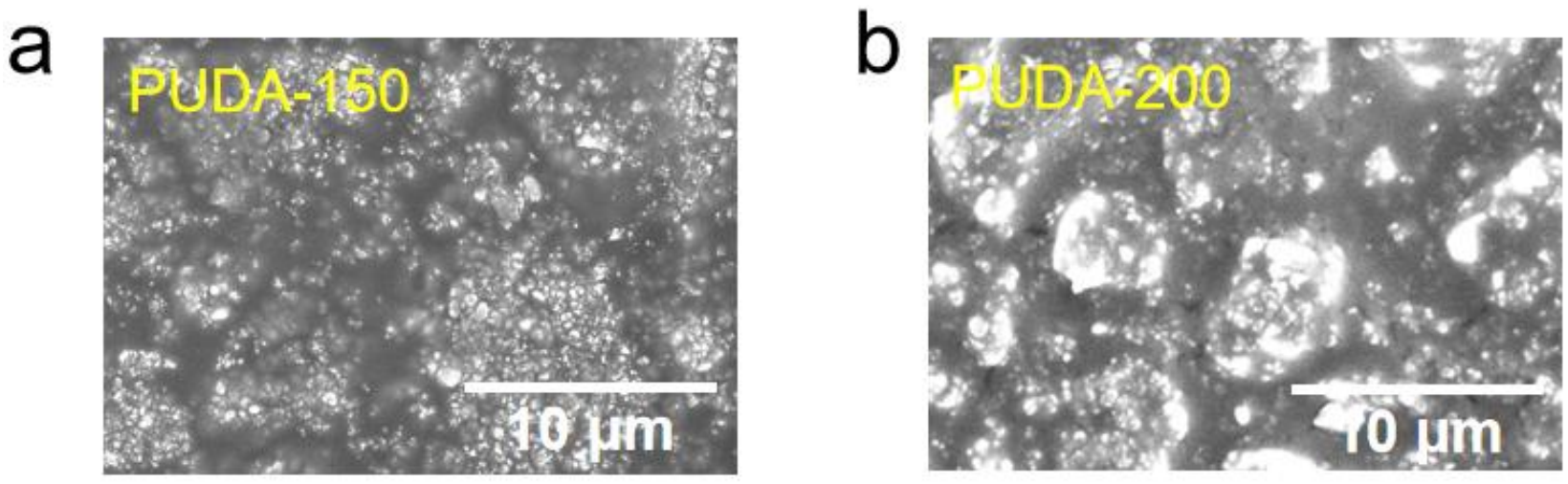

Supplementary Fig. 6 Surface SEM images of (a) PUDA-150 and (b) PUDA-200.

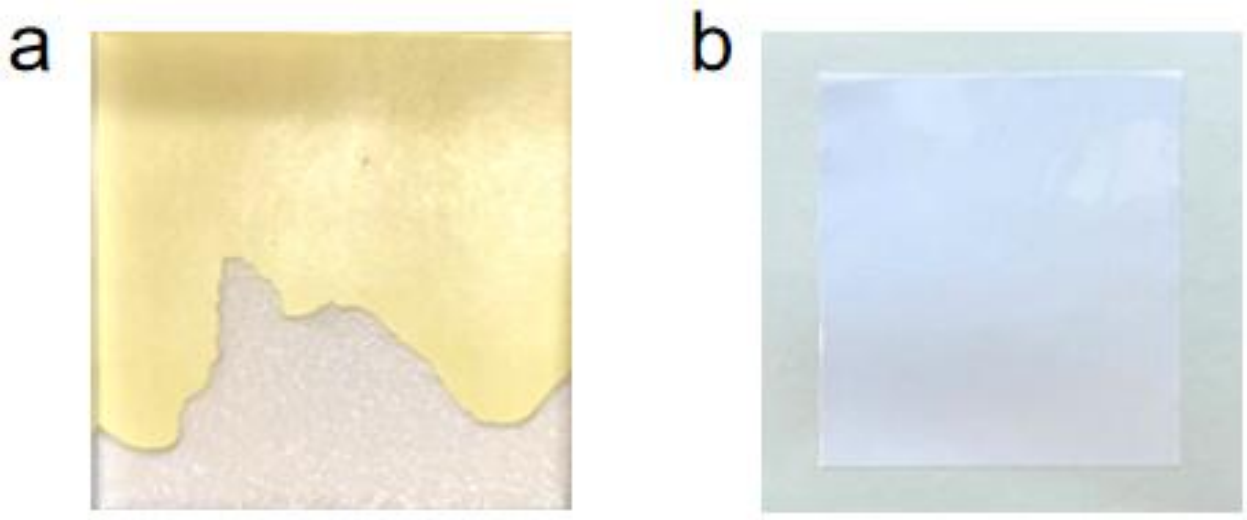

Supplementary Fig. 7 Comparison of the film-forming ability of (a) PEO-100 and (b) PUDA-200.

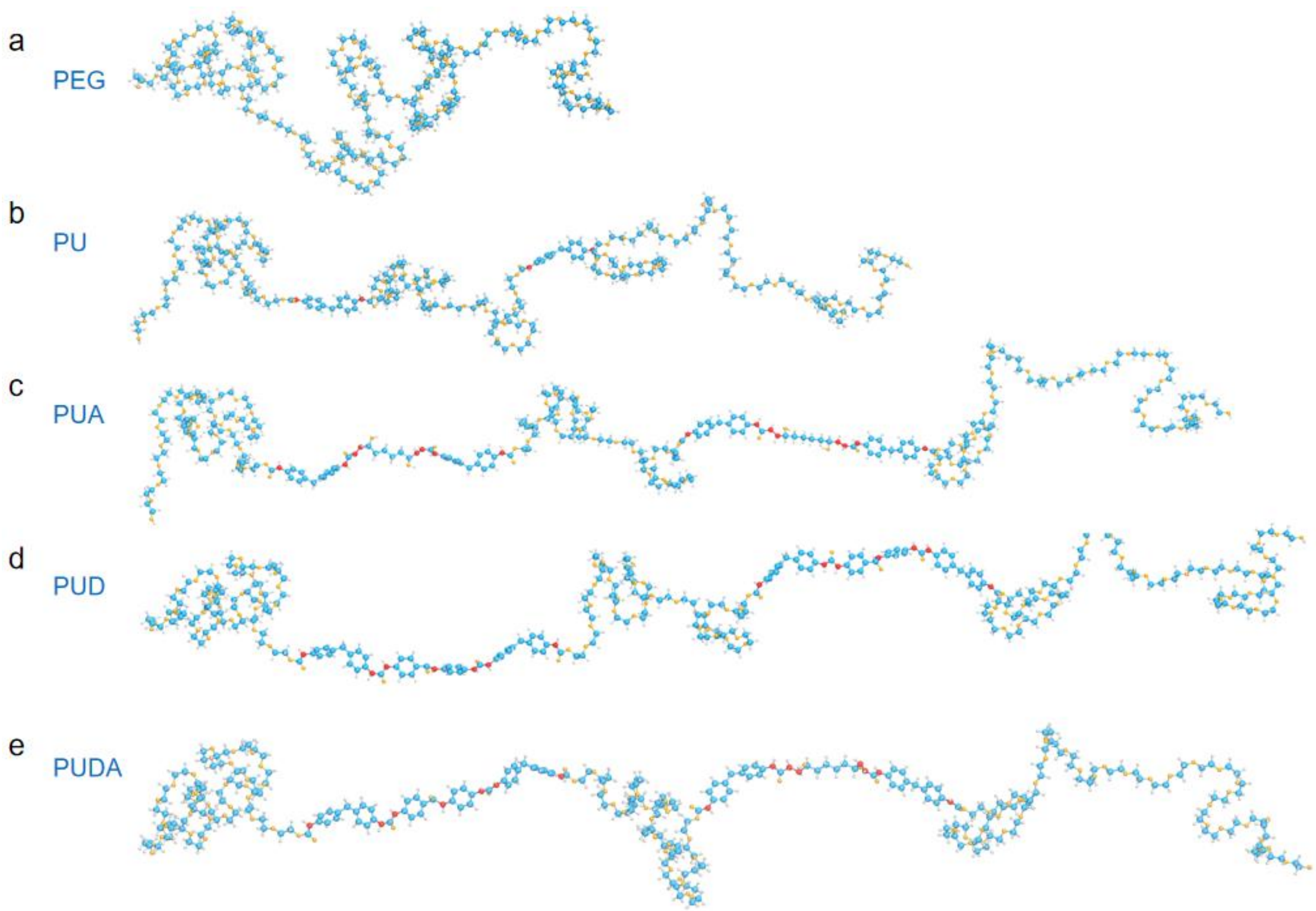

Supplementary Fig. 8 Atomic structures of (a) PEG, (b) PU, (c) PUA, (d) PUD, and (e) PUDA. Different colored spheres represent atoms: blue, C; red, N; yellow, O; gray, H.

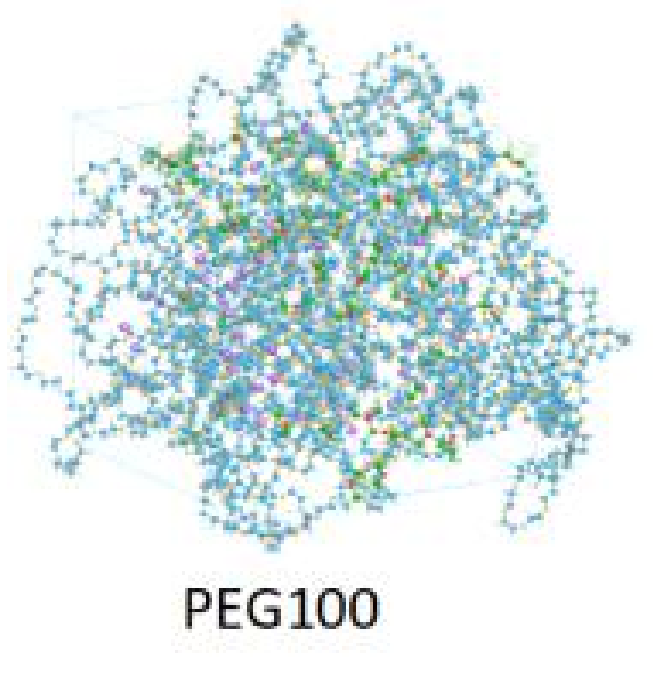

Supplementary Fig. 9 Snapshots of PEG-based solid electrolyte molecular structure. Different colored spheres represent atoms: purple, Li; green, S; blue, C; red, N; sky blue, F; yellow, O; gray, H.

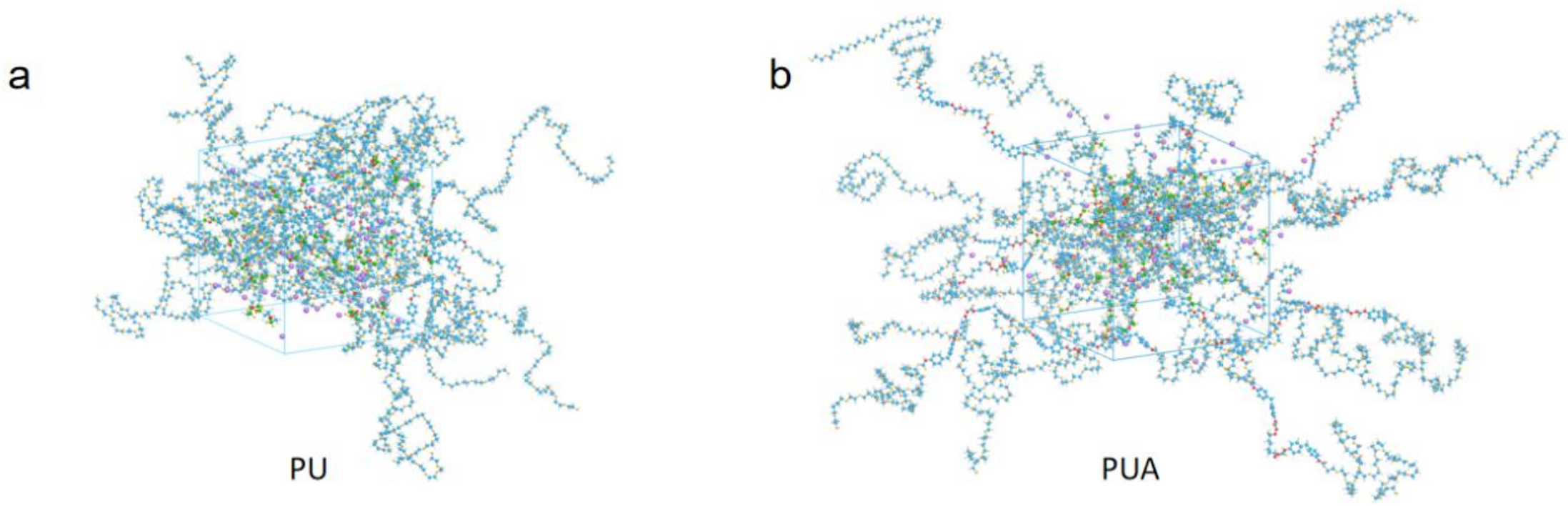

Supplementary Fig. 10 Snapshots of (a) PU-based and (b) PUA-based solid electrolyte molecular structure. Different colored spheres represent atoms: purple, Li; green, S; blue, C; red, N; sky blue, F; yellow, O; gray, H.

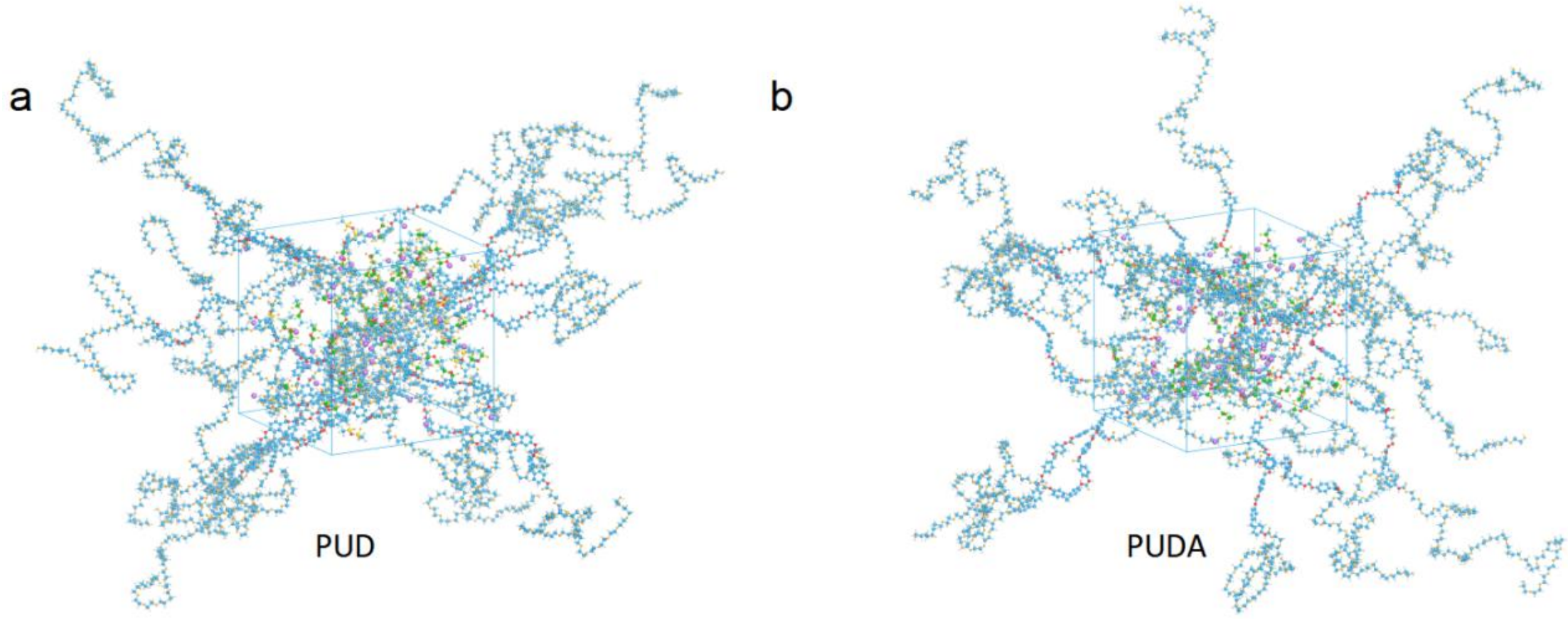

Supplementary Fig. 11 Snapshots of (a) PUD-based and (b) PUDA-based solid electrolyte molecular structure. Different colored spheres represent atoms: purple, Li; green, S; blue, C; red, N; sky blue, F; yellow, O; gray, H.

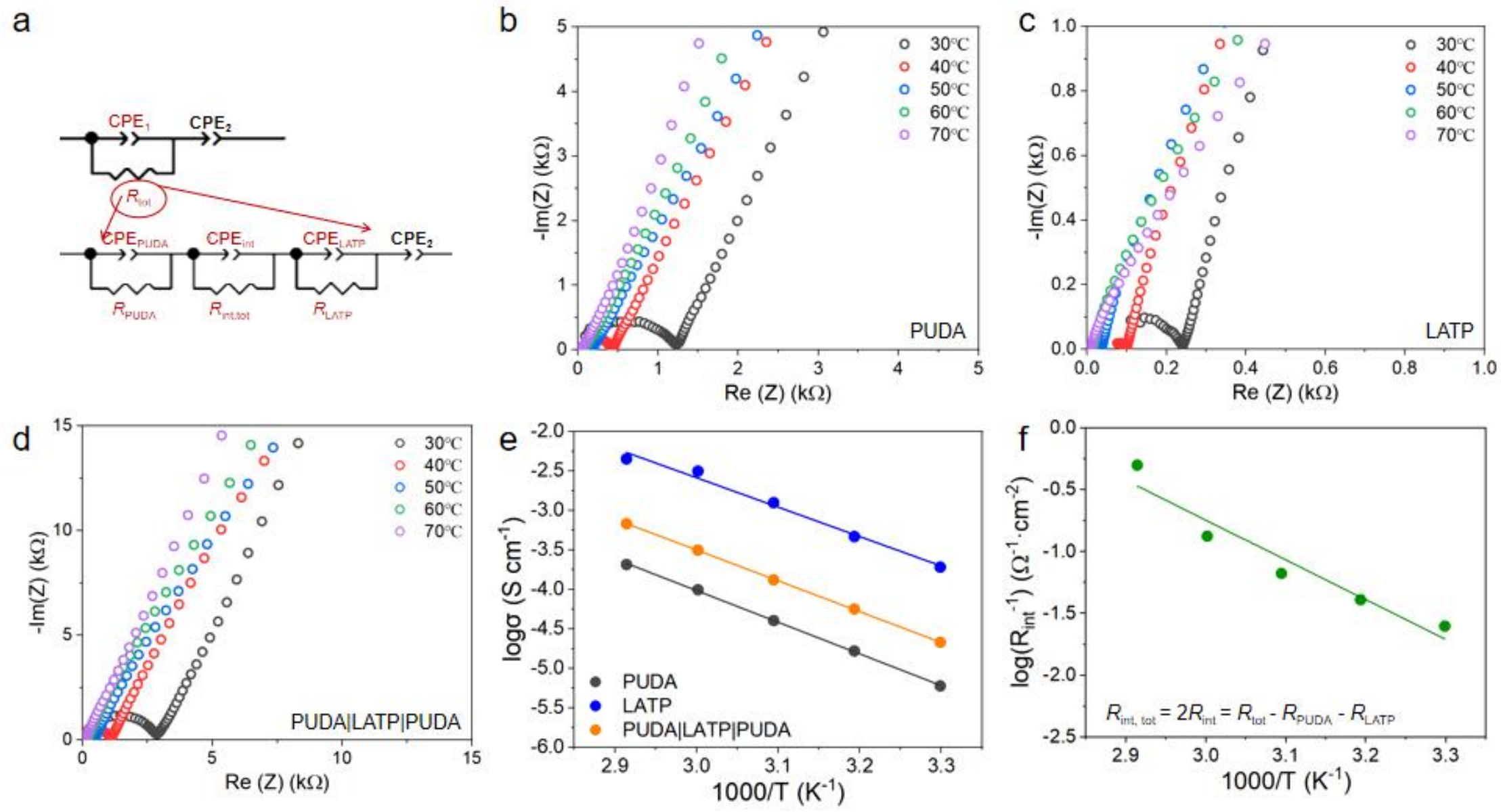

Supplementary Fig. 12 Interfacial resistance analysis of the PUDA|LATP interface. (a) Equivalent circuit model of the SS|PUDA|LATP|PUDA|SS symmetric cell. Constant phase elements (CPEs) are used instead of capacitances to fit the impedance spectra. (b-d) Nyquist plots of SS|PUDA|SS, SS|LATP|SS, and SS|PUDA|LATP|PUDA|SS cells measured at various temperatures. (e) Ionic conductivity of PUDA, LATP, and the PUDA|LATP|PUDA stack as a function of temperature. (f) Arrhenius plot of $1/R_{int}$, normalized by the contact area of the PUDA-LATP interface.

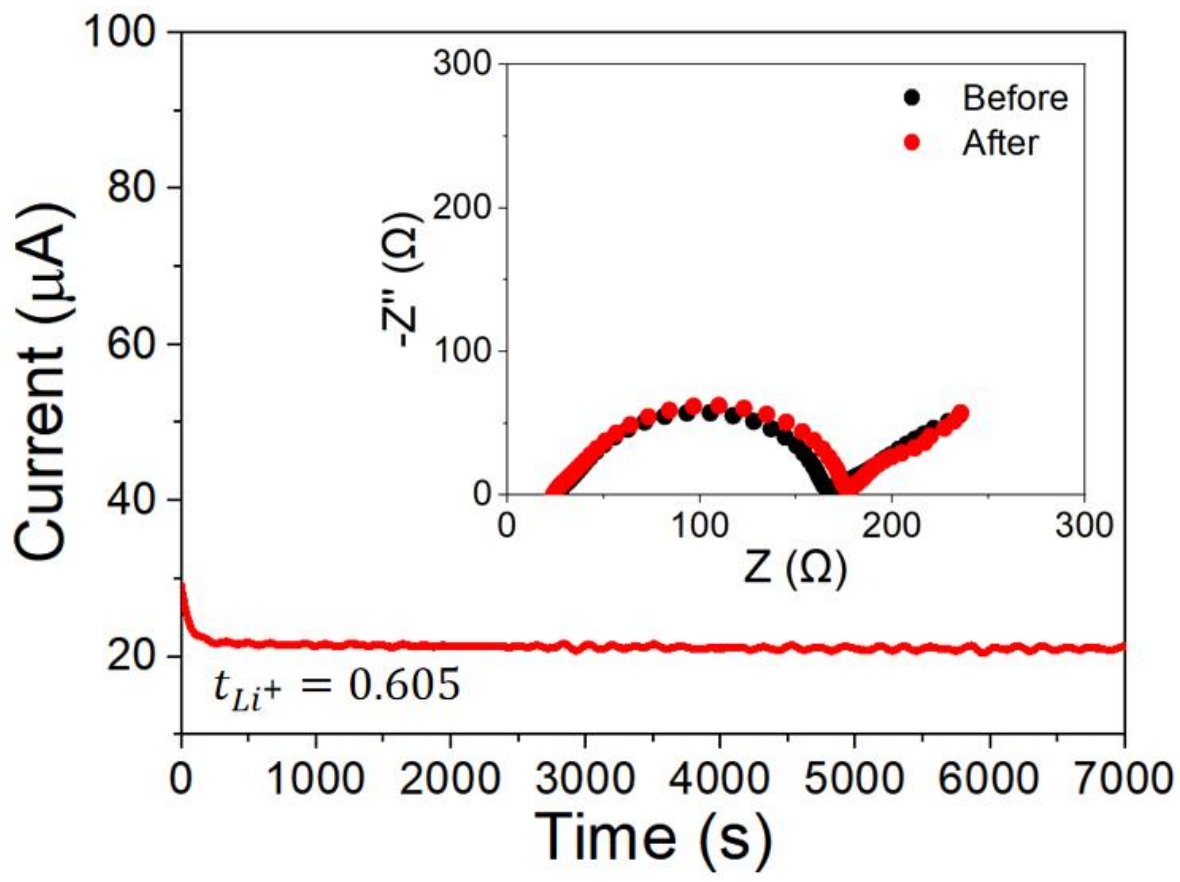

Supplementary Fig. 13 Chronoamperometry curve and AC impedance spectra before and after polarization of the Li|Tri-PUDA-*x*@LATP|Li symmetric cell.

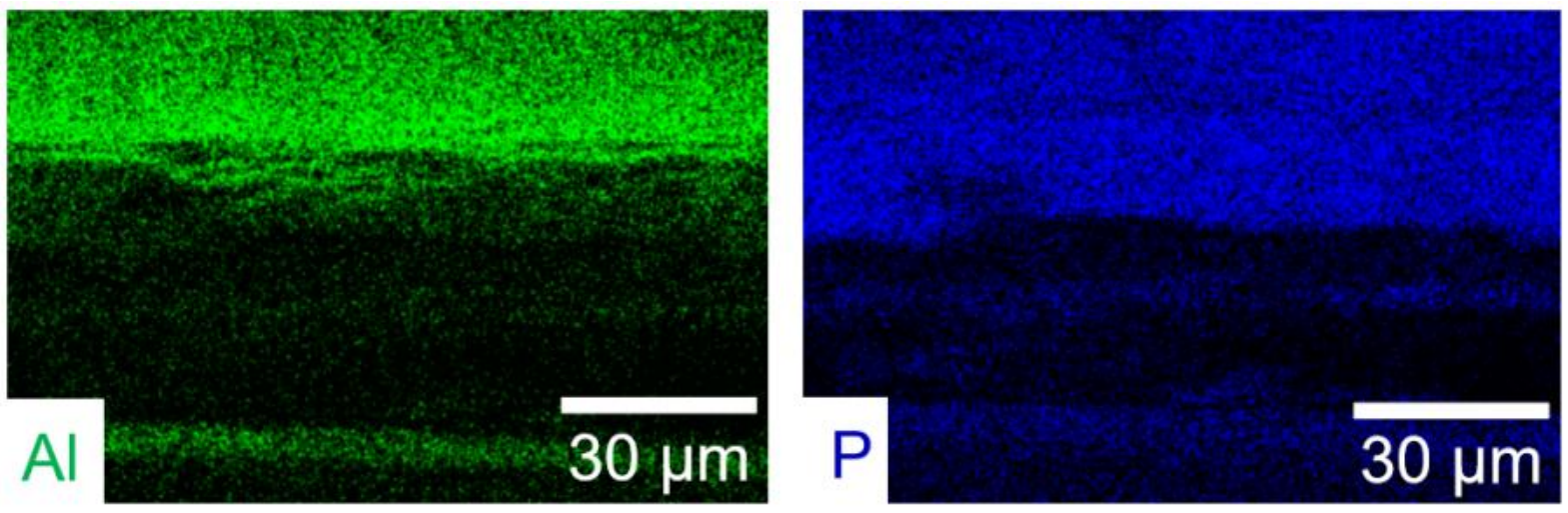

Supplementary Fig. 14 EDS elemental mapping of Tri-PUDA-*x*@LATP.

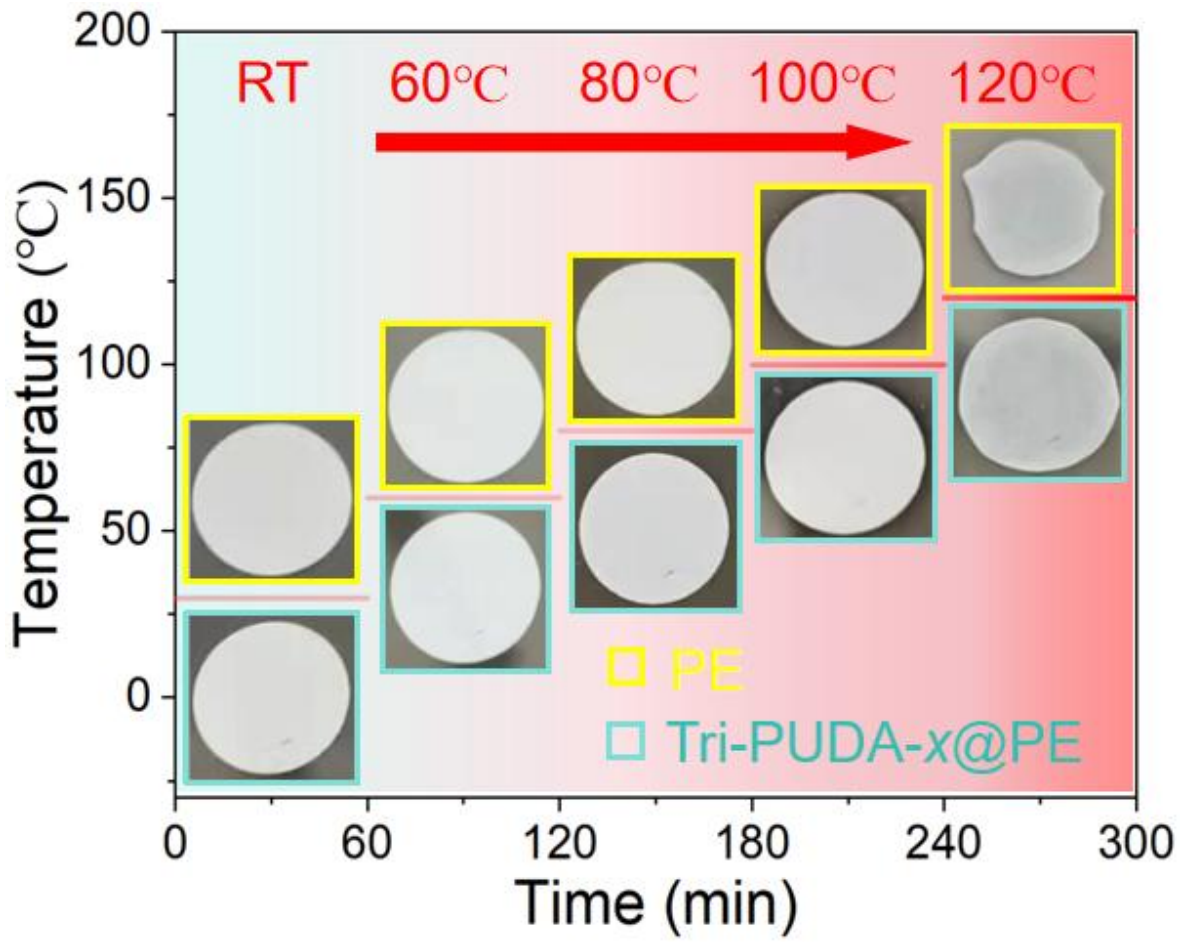

Supplementary Fig. 15 Thermal shrinkage of PE and Tri-PUDA-*x*@PE after 1h isothermal treatment.

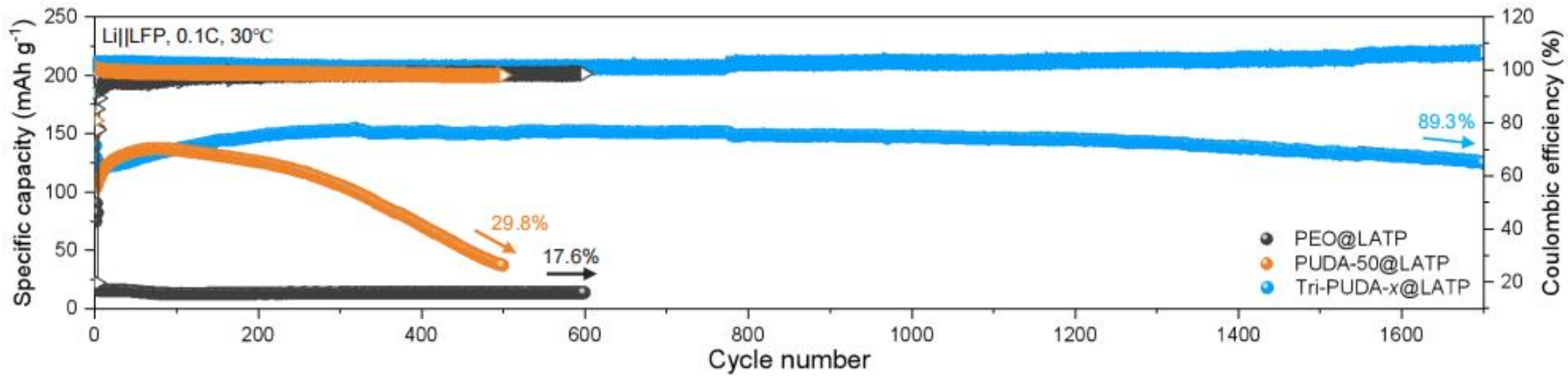

Supplementary Fig. 16 Cycling stability of PEO@LATP, PUDA-50@LATP, and Tri-PUDA-*x*@LATP in solid-state Li||LFP cells at 0.1C, 30°C.

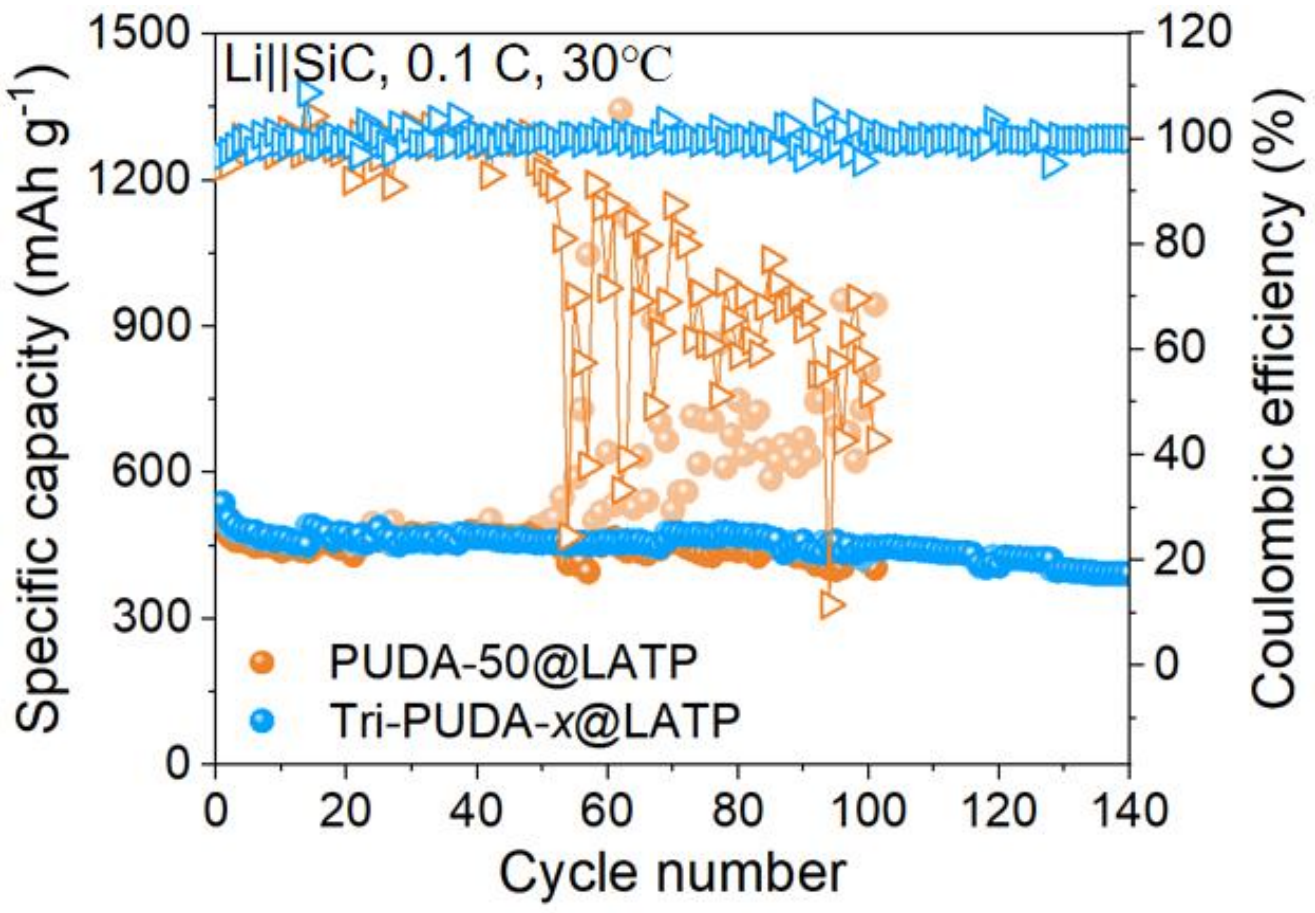

Supplementary Fig. 17 Cycling performance of solid-state Li||SiC half-cells with PUDA-50@LATP and Tri-PUDA-*x*@LATP at 0.1C, 30℃.

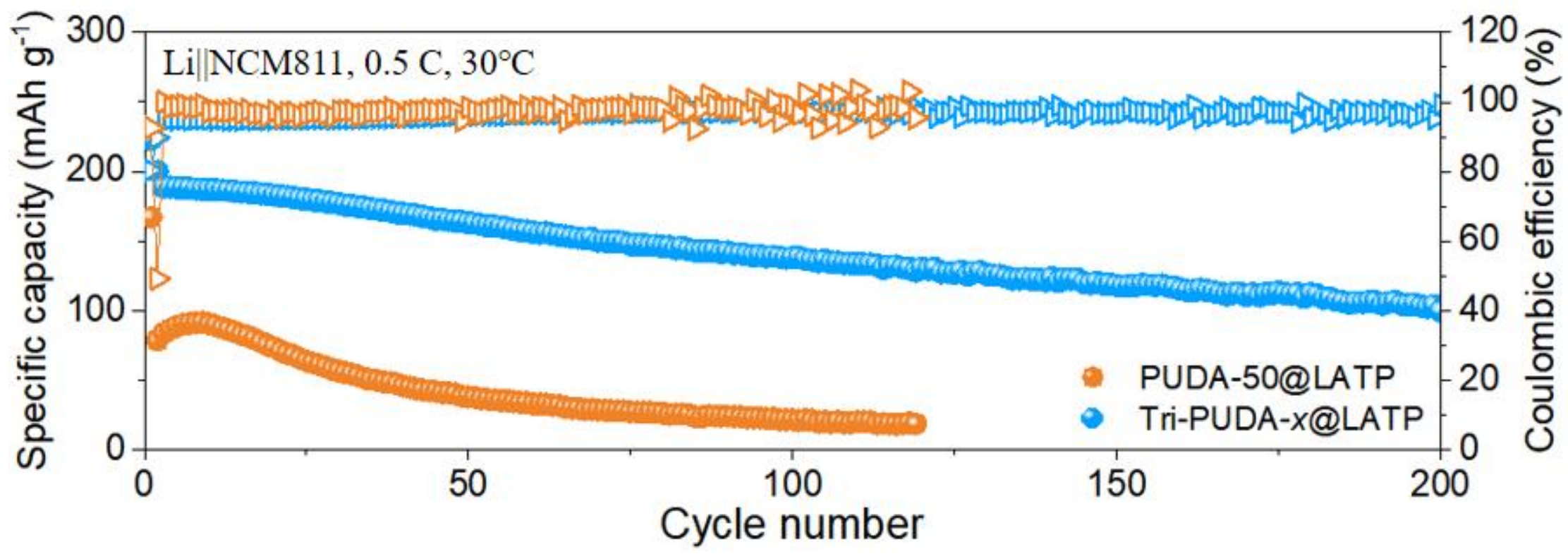

Supplementary Fig. 18 Cycling stability of solid-state Li||NCM811 cells with PUDA-50@LATP and Tri-PUDA-*x*@LATP at 0.5C, 30°C.

Supplementary Table 1. Comparison of stress-strain values of composite solid electrolytes.

Number	Strain (%)	Stress (MPa)	References
1	1.3	5.28	Ref. 1
2	10	4.515	Ref. 2
3	13	3	Ref. 3
4	20	4.5	Ref. 4
5	25	4.48	Ref. 5
6	40	3.1	Ref. 6
7	45	3.2	Ref. 7
8	45	8.5	Ref. 8
9	55	3	Ref. 9
10	60	0.8	Ref. 10
11	60	5.9	Ref. 11
12	70	4.533	Ref. 12
13	80	0.8	Ref. 13
14	115.5	2.85	Ref. 14
15	140	0.6	Ref. 15
16	170	4.2	Ref. 16
17	180	0.45	Ref. 17
18	185	3.3	Ref. 18
19	200	1.49	Ref. 19
20	213.5	12.3	Ref. 20
21	273	0.48	Ref. 21
22	300	4.43	Ref. 22
23	300	10.5	Ref. 23
24	377	0.63	Ref. 24
25	420	0.28	Ref. 25
26	511	11.9	Ref. 26
27	600	24	Ref. 27

28	893.2	0.86	Ref. 28
29	1045	2	Ref. 29
30	1131	15.97	Ref. 30
31	1400	25.3	Ref. 31
32	1500	5	Ref. 32
33	1600	15.6	Ref. 33
34	1700	27.83	Ref. 34
35	1900	23	Ref. 35
36	2000	1.28	Ref. 36
37	2000	88.3	Ref. 37
38	2050	76.5	Ref. 38
39	2300	13	Ref. 39
40	2700	0.46	Ref. 40
41	3175	3.8	Ref. 41
42	4000	0.8	Ref. 42

Supplementary Table 2. Comparison of cycling performance of Li||Li symmetric cells.

Number	Current density/capacity (mA cm^{-2}/mAh cm^{-2})	Polarization voltage (mV)	Cycling time (h)	References
1	0.1/0.1 (60°C)	50	2115	Ref. 30
2	0.1/0.1 (60°C)	20	440	Ref. 43
3	0.1/0.1 (50°C)	100	220	Ref. 44
4	0.1/0.1 (RT)	50	600	Ref. 45
5	0.1/0.1 (45°C)	70	887	Ref. 46
6	0.1/0.1 (30°C)	220	750	Ref. 47
7	0.1/0.1 (60°C)	68	1200	Ref. 1
8	0.1/0.1 (60°C)	40	930	Ref. 19
9	0.1/0.05 (Temp N/A)	5.6	220	Ref. 48
10	0.1/- (Temp N/A)	50	100	Ref. 49
11	0.1/0.1 (50°C)	50	1000	Ref. 50
12	0.1/- (60°C)	17	300	Ref. 51
13	0.1/0.1 (RT)	34	850	Ref. 52
14	0.025/0.05 (60°C)	9	400	Ref. 28
15	0.1/0.05 (RT)	20	500	Ref. 53
16	0.5/0.5 (Temp N/A)	26	500	Ref. 54
17	0.05&0.01/- (60°C)	200	300	Ref. 9

Supplementary Table 3. Fitting parameters and errors for the equivalent circuit of the SS|PUDA|LATP|PUDA|SS symmetric cell.

Electrolytes	Temperature (°C)	R (Ω)	Fitting error (χ^2)
LATP	30	239.8	0.0025761
	40	98.3	0.0015912
	50	36.6	0.0013296
	60	14.6	0.0008716
	70	10.2	0.0200660
PUDA	30	1213.2	0.0006116
	40	437.5	0.0015012
	50	180.3	0.0013822
	60	73.4	0.0014149
	70	35.1	1.2072E13
PUDA|LATP|PUDA	30	2826.8	0.0011153
	40	1071.5	0.0090058
	50	457.6	0.0058166
	60	191.6	0.0050429
	70	88.9	0.0090058

References

1. Zheng, X. et al. Bridging $Li_7La_3Zr_2O_{12}$ Nanofibers with poly(ethylene oxide) by coordination bonds to enhance the cycling stability of all-solid-state lithium metal batteries. *ACS Appl. Mater. Interfaces*. **14**, 5346-5354 (2022).
2. Wang, C. et al. Soy protein as a dual-functional bridge enabling high performance solid electrolyte for Li metal batteries. *J. Power Sources*. **620**, 235260 (2024).
3. Han, W., Li, G. & Zhang, J. Diversifying ion-transport pathways of composite solid electrolytes for high-performance solid-state lithium-metal batteries. *ACS Appl. Mater. Interfaces*. **16**, 27280-27290 (2024).
4. Yan, C. et al. Garnet-rich composite solid electrolytes for dendrite-free, high-rate, solid-state lithium-metal batteries. *Energy Storage Mater.* **26**, 448-456 (2020).
5. Cheng, H., Yan, C., Orenstein, R., Chang, L. & Zhang, X. $Li_{6.28}La_3Zr_2Al_{0.24}O_{12}$-reinforced single-ion conducting composite polymer electrolyte for room-temperature Li-metal batteries. *J. Electrochem. Soc.* **171**, (2024).
6. Li, Y. et al. Polymer nanofibers framework composite solid electrolyte with lithium dendrite suppression for long life all-solid-state lithium metal battery. *Chem. Eng. J.* **440**, 135816 (2022).
7. Da, X. et al. CO_2 -assisted induced self-assembled aramid nanofiber aerogel composite solid polymer electrolyte for all-solid-state lithium-metal batteries. *Adv. Energy Mater.* **14**, 2303527 (2024).
8. Xiang, H. et al. 3D core-shell nanofibers framework and functional ceramic nanoparticles synergistically reinforced composite polymer electrolytes for high-performance all-solid-state lithium metal battery. *Chin. Chem. Lett.* **35**, 109182 (2024).
9. Zhao, Y. et al. Polymer electrolyte with dual functional groups designed via theoretical calculation for all-solid-state lithium batteries. *J. Power Sources*. **450**, 227614 (2020).
10. Yang, G. et al. Anomalously high elastic modulus of a poly(ethylene oxide)-based composite electrolyte. *Energy Storage Mater.* **35**, 431-442 (2021).
11. Wu, M. et al. 3D Coral-like LLZO/PVDF composite electrolytes with enhanced ionic conductivity and mechanical flexibility for solid-state lithium batteries. *ACS Appl. Mater. Interfaces*. **12**, 52652-52659 (2020).
12. Yang, J. et al. Crosslinked polymer-in-salt solid electrolyte with multiple ion transport paths for solid-state lithium metal batteries. *Energy Storage Mater.* **64**, 103088 (2024).
13. Lee, E. J. et al. Poly(urethane acrylate)-based gel polymer films for mechanically stable, transparent, and highly conductive polymer electrolyte applications. *J. Appl. Polym. Sci.* **134**, 45009 (2017).
14. Ren, Z. et al. Insight into the integration way of ceramic solid-state electrolyte fillers in the composite electrolyte for high performance solid-state lithium metal battery. *Energy Storage Mater.* **51**, 130-138 (2022).
15. Liu, Y. et al. Composition modulation and structure design of Inorganic-in-polymer composite solid electrolytes for advanced lithium batteries.

Small. **16**, 1902813 (2020).
16. Zhai, P. et al. Thin laminar composite solid electrolyte with high ionic conductivity and mechanical strength towards advanced all-solid-state lithium–sulfur battery. *J. Mater. Chem. A*. **8**, 23344-23353 (2020).
17. Li, J., Yang, L., Zhang, H. & Ji, X. Self-healing composite solid electrolytes with enhanced Li+ transport and mechanical properties for safe lithium metal batteries. *Chem. Eng. J.* **438**, 135418 (2022).
18. Cui, P. et al. High ion conductivity based on a polyurethane composite solid electrolyte for all-solid-state lithium batteries. *Rsc Adv.* **12**, 3828-3837 (2022).
19. Li, Y. et al. boosting the ion mobility in solid polymer electrolytes using hollow polymer nanospheres as an additive. *ACS Appl. Mater. Interfaces*. **14**, 18360-18372 (2022).
20. Wang, T. et al. Engineering a flexible and mechanically strong composite electrolyte for solid-state lithium batteries. *J. Energy Chem.* **46**, 187-190 (2020).
21. Song, J. et al. Revealing the effect of double bond-modified $Li_{6.75}La_3Zr_{1.75}Ta_{0.25}O_{12}$ on the Li-ion conduction of composite solid electrolytes. *Mater. Today Energy*. **43**, 101583 (2024).
22. Li, Y. et al. Bacterial cellulose composite solid polymer electrolyte with high tensile strength and lithium dendrite inhibition for long life battery. *Energy Environ. Mater.* **4**, 434-443 (2021).
23. Zhu, L. et al. Tunneling interpenetrative lithium ion conduction channels in polymer-in-ceramic composite solid electrolytes. *J. Am. Chem. Soc.* **146**, 6591-6603 (2024).
24. Huang, Z. X., Zhang, T., Zhang, Z. P., Rong, M. Z. & Zhang, M. Q. Highly ionic conductive, self-healing, $Li_{10}GeP_2S_{12}$-filled composite solid electrolytes based on reversibly interlocked macromolecule networks for lithium metal batteries with improved cycling stability. *ACS Appl. Mater. Interfaces*. **16**, 42736-42747 (2024).
25. Lee, M. J. et al. Elastomeric electrolytes for high-energy solid-state lithium batteries. *Nature*. **601**, 217-222 (2022).
26. Wang, Q. et al. Ultrathin, Mechanically Robust Quasi-Solid Composite Electrolyte for Solid-State Lithium Metal Batteries. *ACS Appl. Mater. Interfaces*. **16**, 22482-22492 (2024).
27. Wang, H. et al. Lithiated copper polyphthalocyanine with extended π-conjugation induces LiF-rich solid electrolyte interphase toward long-life solid-state lithium-metal batteries. *Adv. Energy Mater.* **13**, 2204425 (2023).
28. Hu, Z. et al. Hydroxyl-rich single-ion conductors enable solid hybrid polymer electrolytes with excellent compatibility for dendrite-free lithium metal batteries. *J. Membr. Sci.* **657**, 120666 (2022).
29. Li, Y. et al. A composite solid polymer electrolyte incorporating MnO_2 nanosheets with reinforced mechanical properties and electrochemical stability for lithium metal batteries. *J. Mater. Chem. A*. **8**, 2021-2032 (2020).
30. Yi, X. et al. Achieving balanced performance and safety for manufacturing all-solid-state lithium metal batteries by polymer base adjustment. *Adv. Energy Mater.* **15**, 2404973 (2025).

31. Li, S. et al. Metal-organic framework-derived elastic solid polymer electrolytes enabled by covalent crosslinking for high-performance lithium metal batteries. *Adv. Funct. Mater.* **35**, 2415495 (2025).
32. Wu, L. et al. Flame-retardant polyurethane-based solid-state polymer electrolytes enabled by covalent bonding for lithium metal batteries. *Adv. Funct. Mater.* **34**, 2310084 (2024).
33. Hashimoto, K. et al. Strain-induced crystallization and phase separation used for fabricating a tough and stiff slide-ring solid polymer electrolyte. *Sci. Adv.* **9**, eadi8505 (2023).
34. Chen, J. et al. Phase-locked constructing dynamic supramolecular ionic conductive elastomers with superior toughness, autonomous self-healing and recyclability. *Nat. Commun.* **13**, 4868 (2022).
35. Bao, J. et al. Polycarbonate-based polyurethane as a polymer electrolyte matrix for all-solid-state lithium batteries. *J. Power Sources*. **389**, 84-92 (2018).
36. Lv, N. et al. PEO-based composite solid electrolyte for lithium battery with enhanced interface structure. *J. Alloy. Compd.* **938**, 168675 (2023).
37. Pei, F. et al. Interfacial self-healing polymer electrolytes for long-cycle solid-state lithium-sulfur batteries. *Nat. Commun.* **15**, 351 (2024).
38. Pei, F. et al. Multisite crosslinked poly(ether-urethane)-based polymer electrolytes for high-voltage solid-state lithium metal batteries. *Adv. Mater.* **36**, 2409269 (2024).
39. Huang, Q. et al. Supremely elastic gel polymer electrolyte enables a reliable electrode structure for silicon-based anodes. *Nat. Commun.* **10**, 5586 (2019).
40. Xu, H. et al. Deciphering and integrating functionalized side chains for high ion-conductive elastic ternary copolymer solid-state electrolytes for safe lithium metal batteries. *Angewandte Chemie International Edition*. **63**, e202406637 (2024).
41. Zheng, T., Cui, X., Chu, Y., Li, H. & Pan, Q. Ultrahigh elastic polymer electrolytes for solid-state lithium batteries with robust interfaces. *ACS Appl. Mater. Interfaces*. **14**, 5932-5939 (2022).
42. Zhang, N., He, J., Han, W. & Wang, Y. Composite solid electrolyte PEO/SN/$LiAlO_2$ for a solid-state lithium battery. *J. Mater. Sci.* **54**, 9603-9612 (2019).
43. Guo, Q. et al. Poly(ethylene glycol) brush on $Li_{6.4}La_3Zr_{1.4}Ta_{0.6}O_{12}$ towards intimate interfacial compatibility in composite polymer electrolyte for flexible all-solid-state lithium metal batteries. *J. Power Sources*. **498**, 229934 (2021).
44. Porcarelli, L. et al. Single-ion conducting polymer nanoparticles as functional fillers for solid electrolytes in lithium metal batteries. *ACS Appl. Mater. Interfaces*. **13**, 54354-54362 (2021).
45. Jia, M., Bi, Z., Shi, C., Zhao, N. & Guo, X. Air-stable dopamine-treated garnet ceramic particles for high-performance composite electrolytes. *J. Power Sources*. **486**, 229363 (2021).
46. Tong, R., Chen, L., Shao, G., Wang, H. & Wang, C. An integrated solvent-free modification and composite process of $Li_{6.4}La_3Zr_{1.4}Ta_{0.6}O_{12}$/Poly(ethylene oxide)

solid electrolytes: Enhanced compatibility and cycle performance. *J. Power Sources*. **492**, 229672 (2021).
47. Peng, L. et al. Enhanced ionic conductivity and interface compatibility of PVDF-LLZTO composite solid electrolytes by interfacial maleic acid modification. *J. Colloid. Interface. Sci.* **613**, 368-375 (2022).
48. Dubey, B. P., Sahoo, A., Thangadurai, V. & Sharma, Y. Fabrication of ultra-thin, flexible, dendrite-free, robust and nanostructured solid electrolyte membranes for solid-state Li-batteries. *J. Mater. Chem. A*. **10**, 12196-12212 (2022).
49. Zhao, E., Guo, Y., Zhang, A., Wang, H. & Xu, G. Polydopamine coated TiO_2 nanofiber fillers for polyethylene oxide hybrid electrolytes for efficient and durable all solid state lithium ion batteries. *Nanoscale*. **14**, 890-897 (2022).
50. Tong, R. et al. Constructing the lithium polymeric salt interfacial phase in composite solid-state electrolytes for enhancing cycle performance of lithium metal batteries. *Chem. Eng. J.* **442**, 136154 (2022).
51. Chen, W. et al. Bridging interparticle Li^+ conduction in a soft ceramic oxide electrolyte. *J. Am. Chem. Soc.* **143**, 5717-5726 (2021).
52. Yang, H. et al. Chemical interaction and enhanced interfacial ion transport in a ceramic nanofiber–polymer composite electrolyte for all-solid-state lithium metal batteries. *J. Mater. Chem. A* **8**, 7261-7272 (2020).
53. Zhang, Z. et al. Dendrite-free lithium–metal batteries at high rate realized using a composite solid electrolyte with an ester–PO_4 complex and stable interphase. *J. Mater. Chem. A* **7**, 23173-23181 (2019).
54. Gao, Y. et al. Polyurethane/LLZTO solid electrolyte with excellent mechanical strength and electrochemical property for advanced lithium metal battery. *Chem. Eng. J.* **474**, 145446 (2023).
