# Supplementary material for: A mechano-integrated gradient electrolyte for long-cycling solid-state lithium metal batteries
Source: Nat Commun. 2026 Jun 24;17:8277. doi: 10.1038/s41467-026-74573-0 (PMC13470014; doi:10.1038/s41467-026-74573-0)
Supplement: Supplementary file 2 [file 41467_2026_74573_MOESM2_ESM.pdf]

Inventory of Supplementary items

Supplementary data 1: Atomic structure of ADH.

Supplementary data 2: Atomic structure of DABA.

Supplementary data 3: Atomic structure of MDI.

Supplementary data 4: Molecular structure of PEG-based polymer electrolyte.

Supplementary data 5: Molecular structure of PU-based polymer electrolyte.

Supplementary data 6: Molecular structure of PUA-based polymer electrolyte.

Supplementary data 7: Molecular structure of PUD-based polymer electrolyte.

Supplementary data 8: Molecular structure of PUDA-based polymer electrolyte.
