# Supplementary material for: A mechano-integrated gradient electrolyte for long-cycling solid-state lithium metal batteries
Source: Nat Commun. 2026 Jun 24;17:8277. doi: 10.1038/s41467-026-74573-0 (PMC13470014; doi:10.1038/s41467-026-74573-0)
Supplement: Supplementary file 3 [file 41467_2026_74573_MOESM4_ESM.pdf]

nature portfolio

Peer Review File

A mechano-integrated gradient electrolyte for long-cycling solid-state lithium metal batteries

Corresponding Author: Professor Wending Pan

Parts of this Peer Review File have been redacted as indicated to remove third-party material.

This file contains all reviewer reports in order by version, followed by all author rebuttals in order by version.

Version 0:

Reviewer comments:

Reviewer #1

(Remarks to the Author)
This work proposed a new design of solid polymer composite electrolyte, the mechano-integrated gradient electrolyte. In this design LATP, a fast ion conducting oxide material, is dispersed into a polymer framework that consists of dynamic hydrogen bonding framework. Different layers of this composite electrolyte with different ceramic concentration were then stacked together to form the gradient structure. The electrolyte was shown to possess very high ionic conductivity, high mechanical strength, and high resistant to dendrite formation and redox decomposition. Overall, I found this paper interesting, and it is worth publishing in NC platform. But I do have some outstanding concerns. Also, I felt the writing is not that easy to follow. It appears that there are a lot of points the authors want to make, but lacks some sort of focus.

1. It's a bit strange to see 5000%, 200 wt%, and numbers like this. What are these compared with? Or what is the reference state of 100%? I sometimes find it difficult to get what the authors are trying to say.
2. Mechano-integrated is a catchy word. But what does it really mean? What makes it stand out from other compositing strategy?
3. What is the main innovation in this work? Is it the polymer design, or the scalable processing? Or the synthesis of a new polymer?
4. The ionic conductivity is quite good. But how much of this is coming from Li+? Have the authors measured the transference number? Could it be possible coming from H+ or some kind of hydrated proton?
5. What is the fundamental reason for the high echem stability? When I looked at the chemistry of the polymer, some of these functional groups are not electrochemically stable against high potential.

Reviewer #2

(Remarks to the Author)
The manuscript reports a mechano-integrated gradient electrolyte using a highly viscoelastic polymer matrix and LATP solid electrolyte fillers dispersed in the polymer matrix in a graded manner. The authors highlight the advantages of their design: the composite with a high LATP content is placed at the cathode side to improve oxidative stability, the composite with low LATP content remains soft to maintain good interfacial contact with the lithium metal anode, and the composite with intermediate LATP content provides sufficient ionic conduction in the bulk. The overall advancement toward solvent-less composite solid electrolytes is appreciated, especially considering the minimal weight loss at a high temperature of 200 oC. However, the use of EMIM–TFSI ionic liquid should be clearly declared in the main manuscript, and the claimed ion conduction across the organic–inorganic interface requires further justification, given the widely recognized large interfacial resistance between organic and inorganic phases. Therefore, some details and explanations are needed before publication.
1. The author claimed that the asymmetric interfacial demands of lithium metal anodes and high-voltage cathodes remain unmet: the former requires soft, adaptive interfaces to maintain contact during cycling, while the latter necessitates rigid, dense barriers against oxidation. While this understanding is generally appreciated, the description may not be sufficiently accurate. Both anode and cathode interfaces benefit from soft and adaptive contact. Cathode materials are typically oxide-based, and rigid contact is insufficient to ensure good ion conduction, which is a known challenge for oxide-based solid electrolytes on the cathode side. In this work, the ceramic-enriched electrolyte at the cathode appears to primarily improve

oxidative stability rather than mechanical rigidity, which should be clarified to avoid confusion.
2. The formation process of the mechano-integrated gradient electrolyte is not clearly described. After checking both the main manuscript and the Supporting Information, no clear description of how the spatial ceramic gradient is formed can be found. It only becomes apparent from the final figure that the structure is stacked layer by layer. This important fabrication detail should be clarified earlier.
3. The authors claim that the organic–inorganic interphase facilitates Li+ transport. However, it is widely recognized that the interfacial ion transfer resistance between organic and inorganic materials is typically large. Although many composite electrolytes have been reported, their performance improvement is often limited. Fundamental studies (e.g., ACS Omega 2021, 6, 24, 16187–16193) have shown that large interfacial resistance results in limited contribution of LATP particles to Li-ion conduction, with most ions traveling through either the polymer or the LATP phase. Therefore, additional evidence is required to support this claim.
4. EMIM–TFSI ionic liquid is used as a core component in this composite electrolyte and may significantly contribute to the observed ionic conductivity. However, this information is only disclosed in the Supporting Information. The authors should clearly state and discuss its role in the main text. Accordingly, the schematic in Fig. 1b should be revised. The term “all-solid-state” may not be appropriate, as EMIM–TFSI is liquid at room temperature.
5. Fig. 2b requires further explanation of the blue and yellow regions.
6. PUDA-10 and PUDA-100 exhibit ionic conductivities that are approximately one order of magnitude lower. Could this lead to insufficient ion transport near the electrodes, particularly at the cathode side? It is unclear whether these regions could limit the overall cell performance.
7. Fig. 4d presents a very interesting characterization and is greatly appreciated.
8. In Fig. 5d, the reported value of 6.78% might not appear to correspond to the y-axis scale.
9. In Fig. 5e, the Coulombic efficiency exceeding 100% requires explanation.
10. LATP is known to be unstable against lithium metal. The rationale for choosing LATP in this design should be clarified. Could other oxide-based solid electrolytes be applied within the same design framework?

Reviewer #3

(Remarks to the Author)
- Can the PUDA fabricated in this study actually function as a lithium-ion conducting polymer electrolyte in a manner comparable to other polymer electrolytes? Even if lithium-ion solvation is possible through the ether groups, the manuscript also emphasizes that rigidity increases with the incorporation of biphenyl groups, raising concerns about whether conventional segmental motion–assisted ion transport can realistically occur. Please provide a more in-depth explanation of the ionic conductivity value of pristine PUDA shown in Figure 3d and its corresponding transport mechanism.
- How is the filler-loading capacity calculated? Is it appropriate to express this value as exceeding 200 in units of wt%?
- The description that LATP is “spatially graded” in the membrane is somewhat confusing. Moreover, this concept is introduced before Figure 2, whereas the supporting data appear in Figure 3, which may further confuse readers. A reorganization of the manuscript structure seems necessary.
- In this electrolyte membrane, how does lithium-ion transport occur in regions containing only 10 wt% LATP? Even if specific layers exhibit high LATP content and thus good ionic conductivity, layers with low LATP content are likely to determine the membrane's overall ionic conductivity. Therefore, additional clarification and discussion on this point are required.
- In Figure 1d, does “Sp” correspond to the wt% mentioned earlier? If so, the terminology should be unified for consistency.
- Figure 2g suggests that high bulk and shear moduli are beneficial. However, the manuscript does not clearly explain why higher mechanical moduli are advantageous in this context. Further justification is needed.
- What is the origin of the difference in crystallinity between PUDA and PUDA-0?
- In some parts of the manuscript, the thickness of the fabricated electrolyte membrane is described as 12 μm, whereas in other sections it appears to be 40 μm. This inconsistency should be corrected.
- Why does this polymer electrolyte exhibit superior oxidative stability compared to PEO? Additionally, in the LSV experiments used to evaluate the oxidative stability of the polymer electrolyte, what material was used to provide electronic conductivity in the electrode? Without an electronically conductive component in the electrode, proper redox reactions cannot occur, which may lead to significant overestimation of the electrochemical stability window. This is precisely why solid electrolytes are typically evaluated using electrodes containing a substantial amount of carbon conductive additive when determining electrochemical stability windows.
- Why were most of the cell performance data obtained using LFP cathodes? And why was NCM811 used only in the pouch-cell configuration? Furthermore, in Figure 5e, the capacity appears to increase for nearly 200 cycles—what is the underlying reason for this behavior?
- It is highly unusual that none of the cathode electrodes used in this study contains a solid electrolyte. How can the cells operate without a solid electrolyte in the composite cathode? Does the polymer from the polymer electrolyte layer infiltrate into the cathode during cell fabrication? Or is the cathode loading so low that electrochemical cycling is possible simply through contact with the electrolyte membrane, even without electrolyte inside the electrode? If the latter is the case, this configuration is not practical at all. For realistic assessment, the system should be demonstrated at higher practical areal loadings (e.g., ~3–4 mAh cm^{-2}), and therefore, data obtained from electrodes without a solid electrolyte are difficult to accept.

Minor comments
- There are several capitalization errors in units (e.g., MPa), which appear both in the figures and the main text, and should be corrected.
- Superscripts for numbers and units appear to have been inserted by direct copy-and-paste (e.g., from ChatGPT or similar tools) rather than using proper word-processing formatting. As a result, their appearance is inconsistent and should be

revised.
- Figure 6 appears to be partially cut off.

Version 1:

Reviewer comments:

Reviewer #1

(Remarks to the Author)
My concerns have been addressed well.

Reviewer #2

(Remarks to the Author)
We thank the authors for their detailed responses. The manuscript has been significantly improved and most of the previous questions have been addressed. To further strengthen the clarity and robustness of the study, we would like to suggest clarification on the following point:
As mentioned in the response to Comment 3, conducting a SS||SS symmetric cell measurement could further support the evaluation of the interfacial resistance at the organic/inorganic interface and make the argument more convincing.

Reviewer #3

(Remarks to the Author)
The author has addressed all the comments.

Version 2:

Reviewer comments:

Reviewer #2

(Remarks to the Author)
The reviewer appreciates the authors' efforts to improve the manuscript and provide a more comprehensive understanding of the inorganic/organic interface.

Open Access This Peer Review File is licensed under a Creative Commons Attribution 4.0 International License, which permits use, sharing, adaptation, distribution and reproduction in any medium or format, as long as you give appropriate credit to the original author(s) and the source, provide a link to the Creative Commons license, and indicate if changes were made.

In cases where reviewers are anonymous, credit should be given to 'Anonymous Referee' and the source.
The images or other third party material in this Peer Review File are included in the article's Creative Commons license, unless indicated otherwise in a credit line to the material. If material is not included in the article's Creative Commons license and your intended use is not permitted by statutory regulation or exceeds the permitted use, you will need to obtain permission directly from the copyright holder.
To view a copy of this license, visit https://creativecommons.org/licenses/by/4.0/

The main corrections in the manuscript and the responds to the Reviewers' comments are as following:

Reviewer #1: *This work proposed a new design of solid polymer composite electrolyte, the mechano-integrated gradient electrolyte. In this design LATP, a fast ion conducting oxide material, is dispersed into a polymer framework that consists of dynamic hydrogen bonding framework. Different layers of this composite electrolyte with different ceramic concentration were then stacked together to form the gradient structure. The electrolyte was shown to possess very high ionic conductivity, high mechanical strength, and high resistant to dendrite formation and redox decomposition. Overall, I found this paper interesting, and it is worth publishing in NC platform. But I do have some outstanding concerns. Also, I felt the writing is not that easy to follow. It appears that there are a lot of points the authors want to make, but lacks some sort of focus.*

Author Reply: We would like to express our sincere gratitude to the reviewer for the positive and constructive comments. We have carefully addressed all the points raised and revised the manuscript as shown below:

1. It's a bit strange to see 5000%, 200 wt%, and numbers like this. What are these compared with? Or what is the reference state of 100%? I sometimes find it difficult to get what the authors are trying to say.

Author Reply: We sincerely thank the Reviewer for this insightful comment. We realize that the lack of clearly defined reference states for the reported percentages (e.g., 5000% and 200 wt%) could cause confusion. We have now clarified these numbers:

(1) Regarding "**200 wt%**": The notation "wt%" is commonly used in the composite electrolyte literature to denote the mass ratio of inorganic filler to the polymer matrix, where 100 wt% corresponds to equal masses of filler and polymer. We acknowledge that values exceeding 100 wt% can appear unconventional and appreciate the opportunity to clarify. Indeed, the ability of our PUDA matrix to accommodate >200 wt% LATP, meaning the filler mass is more than twice that of the polymer, represents a key material advance. This exceptional loading capability stems directly from the ultra-viscoelastic, hydrogen-bonded network of PUDA, which maintains cohesive film integrity and high elongation (>1477%) even at 200 wt% high filler contents. In contrast, most conventional polymer electrolytes (e.g., PEO, PVDF, PAN) become brittle and lose processability when filler loading exceeds 50–60 wt% (elongation<300%). We have revised the text to explicitly define the wt% reference state in the revised manuscript.

(2) Regarding "**>5000% fracture strain**": This value refers to the engineering fracture strain of the pristine PUDA polymer electrolyte (PUDA-0), measured via standard tensile testing. A >5000% strain signifies that a 2 cm sample can be stretched beyond 1 meter without fracture, demonstrating extraordinary viscoelasticity. This property is the foundation for accommodating high filler loadings and achieving

conformal interfacial contact.

Proposed Text Additions to Manuscript:

In Section 2.1 (Molecular Design), after introducing PUDA-*x* notation (**Page 7**):

The filler loading (*x* in PUDA-*x*) is defined as the weight percentage of LATP relative to the PUDA polymer matrix (e.g., 200 wt% denotes a LATP : PUDA mass ratio of 2:1).

In Section 2.2, when first presenting mechanical data (**Page 14**):

The pristine PUDA matrix (PUDA-0) exhibits exceptional fracture strain exceeding 5000% (**Fig. 2**g), capable of stretching to over fifty times its original length, which surpasses most reported polymer electrolytes (**Fig. 2**h, **Table S1**). This extreme viscoelasticity, coupled with autonomous self-healing, provides the fundamental property required for processing high-filler-load composites.

In the caption of Fig. 2g and Fig. 2h (**Page 16**):

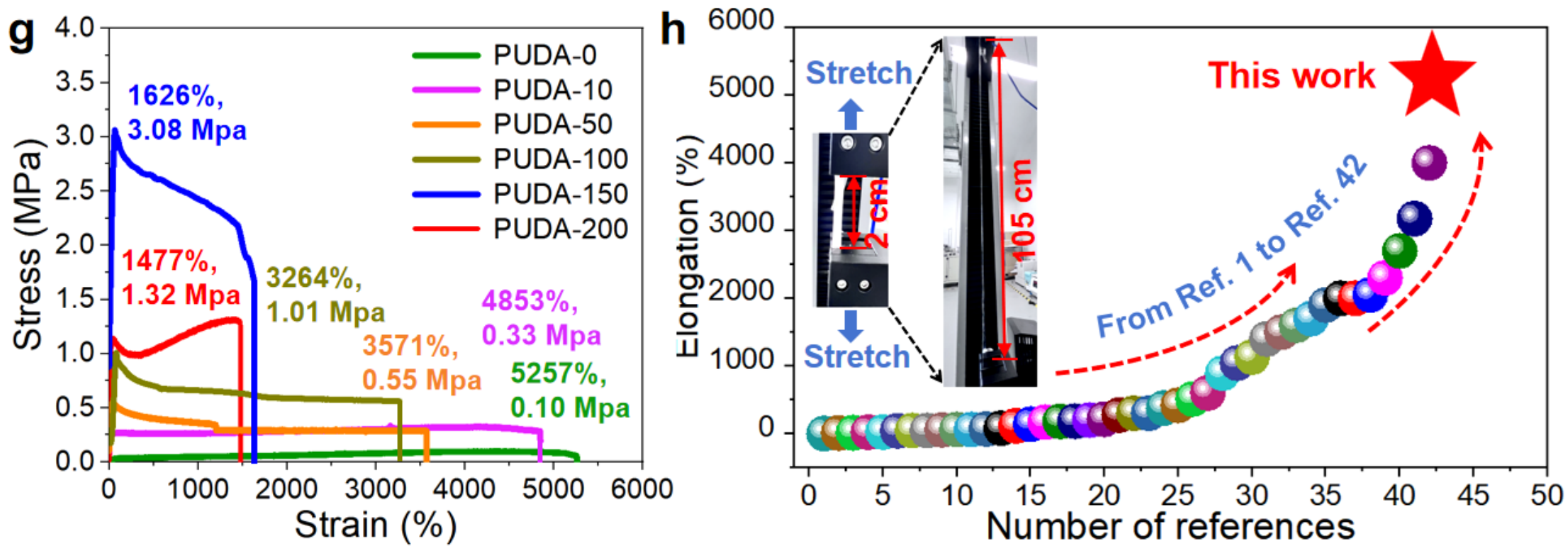

Fig. 2 | g, Stress-strain curves of PUDA-*x* composites. The filler loading *x* is defined as the weight percentage of LATP relative to the PUDA polymer matrix. **h**, Elongation at break (fracture strain) comparison between PUDA-0 and previously reported polymer electrolytes.

2. Mechano-integrated is a catchy word. But what does it really mean? What makes it stand out from other compositing strategy?

Author Reply: We thank the Reviewer for raising this essential point, which allows us to clarify this foundational concept of our work. The term "Mechano-Integrated" is indeed central to our design philosophy and distinguishes our approach from conventional composite or laminated strategies. It describes a single-material system in which mechanical properties are spatially programmed at multiple scales to match location-specific interfacial requirements, while maintaining ionic transport continuity and chemical/mechanical integrity across the entire electrolyte. In essence, "Mechano-Integrated" stands out because it achieves what laminated or blended composites cannot: spatially tailored mechanics without introducing weak interfaces, all while maintaining a single, solution-processable, and continuously ion-conducting

membrane.

(1) Molecular Integration: The polymer matrix (PUDA) is engineered with dynamic hydrogen-bonding motifs (DBDA and ADH), which simultaneously deliver ultra-high elasticity (>5000% fracture strain) and high toughness (>80 MPa tensile strength). This molecular design enables extreme filler loading (>200 wt% LATP) without brittleness, a fundamental limitation in conventional composites.

(2) Microstructural Integration: The inorganic filler (LATP) is not merely blended or stacked, but spatially graded in a continuous manner (10→50→100 wt%). This creates smooth transitions in mechanical modulus and Li^+ flux, eliminating the stress concentration and interfacial impedance typical of laminated heterostructures.

(3) Functional Integration: Within this monolithic gradient, each region is designed to fulfill a distinct mechanical role, soft conformal contact at the anode, rigid dendrite-blocking in the bulk, and dense oxidation protection at the cathode, while remaining ionically continuous via a triphasic (organic/inorganic/interphase) network.

In short, “Mechano-Integrated” means that mechanical adaptation is not an added feature but is embedded into the electrolyte’s architecture from the molecular level upwards, enabling it to reconcile conflicting interfacial requirements within a single, solution-processable, and scalable membrane.

To clarify this concept and highlight what distinguishes our strategy from conventional composite or multilayer designs, we have made the following revisions in the revised manuscript:

In Section 2.1, we have added a dedicated paragraph to explicitly define and contrast the “Mechano-Integrated” approach (**Page 7** and **Page 8**):

The MIGE architecture embodies the “Mechano-Integrated” principle through coherent design across three scales: (i) Molecular integration via dynamic hydrogen bonds, enabling both extreme elongation and high tensile strength; (ii) Microstructural integration via a continuous LATP gradient, ensuring smooth spatial transitions in mechanics and Li^+ flux; and (iii) Functional integration, where compliant anode contact, rigid dendrite suppression, and dense cathode protection are unified within a single, ionically continuous membrane.

Unlike conventional composites with homogenized fillers or simply stacked heterostructures, MIGE is a continuously graded, monolithic architecture. In this design, mechanical properties, such as modulus, adhesion, and viscoelasticity, are spatially tailored to meet the distinct demands of each electrode interface, without introducing internal boundaries that create stress concentrations. This is realized by synergistically coupling a single-phase, ultra-elastic polymer matrix with a continuously graded filler distribution, thereby embedding mechanical adaptation directly into the ion-conduction network.

In the Conclusion (Section 3), the final summary now reinforces the integrative nature of the strategy (**Page 31**):

Crucially, the MIGE represents a mechanically integrated system, as opposed to a

mechanically compromised composite or an assembled laminate. Mechanical functionality is not merely appended but is inherently encoded from the molecular level upward into the electrolyte's morphology and chemistry. This integration enables a single membrane to meet spatially conflicting interfacial demands.

3. What is the main innovation in this work? Is it the polymer design, or the scalable processing? Or the synthesis of a new polymer?

Author Reply: We thank the Reviewer for the opportunity to clarify our contribution. The principal innovation is the integrated **design of a Mechano-Integrated Gradient Electrolyte (MIGE)** that synergistically unites molecular engineering, microstructure architecture, and scalable manufacturing into a coherent platform, not merely one of these aspects in isolation. Specifically, the innovation unfolds across three interdependent levels:

(1) A function-oriented polymer matrix (PUDA). Through rational incorporation of dual hydrogen-bonding extenders (DBDA and ADH), we synthesized a polyurethane elastomer that exhibits unprecedented ultra-viscoelasticity (>5000% fracture strain). This is not simply a "new polymer", but a material whose mechanical properties are precisely tailored to overcome the classic adhesion-brittleness trade-off in composite electrolytes, enabling the subsequent structural and processing advances.

(2) A spatially graded architecture enabled by the polymer's extreme adhesion. The ultra-viscoelastic PUDA matrix can homogeneously incorporate >200 wt% of LATP filler, far exceeding conventional loading limits. This allows us to structure a single, monolithic electrolyte with spatially varying LATP content to decouple the conflicting interfacial demands of Li-metal anodes and high-voltage cathodes.

(3) Inherent compatibility with scalable, solution-based manufacturing. The combination of PUDA's solution processability, low drying temperature (60-80°C), and excellent mechanical integrity (>80 MPa tensile strength) allows the MIGE to be fabricated via high-speed roll-to-roll (R2R) coating (80-100 m/min). The process directly yields flexible, self-standing electrolyte films that require no external stack pressure during cell operation, a critical advantage for practical battery assembly.

Thus, the innovation lies in the interdependent synergy of these three elements: the molecularly designed polymer enables the gradient composite structure, and the resulting mechanical and electrochemical properties in turn enable a scalable, industrially viable fabrication route. This integrated approach directly resolves the long-standing conflict between interfacial compliance and electrochemical robustness in solid-state batteries.

To sharpen this focus, we have revised the Introduction and Conclusion to clearly frame this integrated innovation from the outset:

In the Introduction (Section 1), we now state (**Page 5**):

Herein, we report a Mechano-Integrated Gradient Electrolyte (MIGE) that coalesces molecular design, microstructural engineering, and scalable manufacturing

into a unified platform. We engineer an ultra-viscoelastic polyurethane matrix (PUDA) via dynamic hydrogen-bonding motifs, achieving >5000% fracture strain and exceptional filler-loading capacity (>200 wt%). This enables the construction of a monolithic electrolyte film with a spatially graded LATP architecture (10-100 wt%), which decouples interfacial demands: a compliant anode layer ensures conformal lithium contact, an intermediate percolation layer facilitates rapid ion transport (~10^{-4} S cm^{-1}), and a rigid, ceramic-rich cathode interface delivers oxidation stability beyond 4.9 V. Crucially, the single-phase polymer framework circumvents interfacial degradation while offering high tensile strength (>80 MPa) and full solution processability, key to scalable manufacturing. This work establishes a new design paradigm for solid electrolytes that directly addresses the core mechano-electrochemical coupling issue. The resulting MIGE platform offers a scalable, solution-processable route to high-performance, pressure-free SSLMBs.

In the Conclusion (Section 3), we summarize (**Page 30**):

In this work, we have demonstrated a multi-scale design paradigm for solid electrolytes through the development of a Mechano-Integrated Gradient Electrolyte (MIGE). This approach systematically integrates molecular engineering, mesoscale architecture, and macroscopic functionality to overcome long-standing limitations in conventional solid electrolyte design.

The MIGE architecture represents a fundamental advance beyond homogeneous composite. By spatially grading the LATP content, we decouple the interfacial priorities: ultra-conformal, strain-tolerant contact at the anode and a dense, oxidation-resistant yet still conformal interface at the cathode.

4. The ionic conductivity is quite good. But how much of this is coming from Li^+? Have the authors measured the transference number? Could it be possible coming from H^+ or some kind of hydrated proton?

Author Reply: We thank the Reviewer for pointing out this important aspect. While the current study focuses on the overall ionic conductivity of the mechano-integrated system, we acknowledge that determining the Li^+ transference number would provide deeper mechanistic insight. To directly address this, we have performed potentiostatic polarization measurements (Bruce-Vincent method) on symmetric Li|Tri-PUDA-*x*@LATP|Li cells to determine the Li^+ transference number (t_{Li+}). The results confirm that the ionic conductivity is predominantly contributed by Li^+ migration.The measured t_{Li+} for the Tri-PUDA-50@LATP is 0.61 at 25 °C (see the newly added Fig. S12 and related analysis in the Supporting Information). This value is significantly higher than that of conventional PEO-based electrolytes (typically t_{Li+} < 0.3) and confirms that Li^+ is the primary charge carrier in our system.

We believe the contribution from H^+ or hydrated protons to the observed ionic conductivity is negligible, which is for several reasons: 1) Material synthesis and handling: All electrolyte membranes were vacuum-dried at 80 °C for 12 hours, and cell assembly was performed in an Ar-filled glovebox (H_2O< 0.1 ppm, O_2< 0.1 ppm). The hydrophobic polymer matrix and pre-dried LATP fillers were stored and

processed under inert atmosphere to eliminate moisture. 2) Electrochemical stability: The wide, stable electrochemical window up to 4.9 V vs. Li/Li^+ (Fig. 3f) and the ultra-stable Li plating/stripping over 7500 h (Fig. 4a,b) are inconsistent with proton conduction, which would induce severe parasitic reactions at low potentials. 3) Comparative control: We conducted additional impedance measurements on cells with blocking electrodes (Au|electrolyte|Au). No significant protonic conduction pathway was detected, further confirming the dominant role of Li^+ transport.

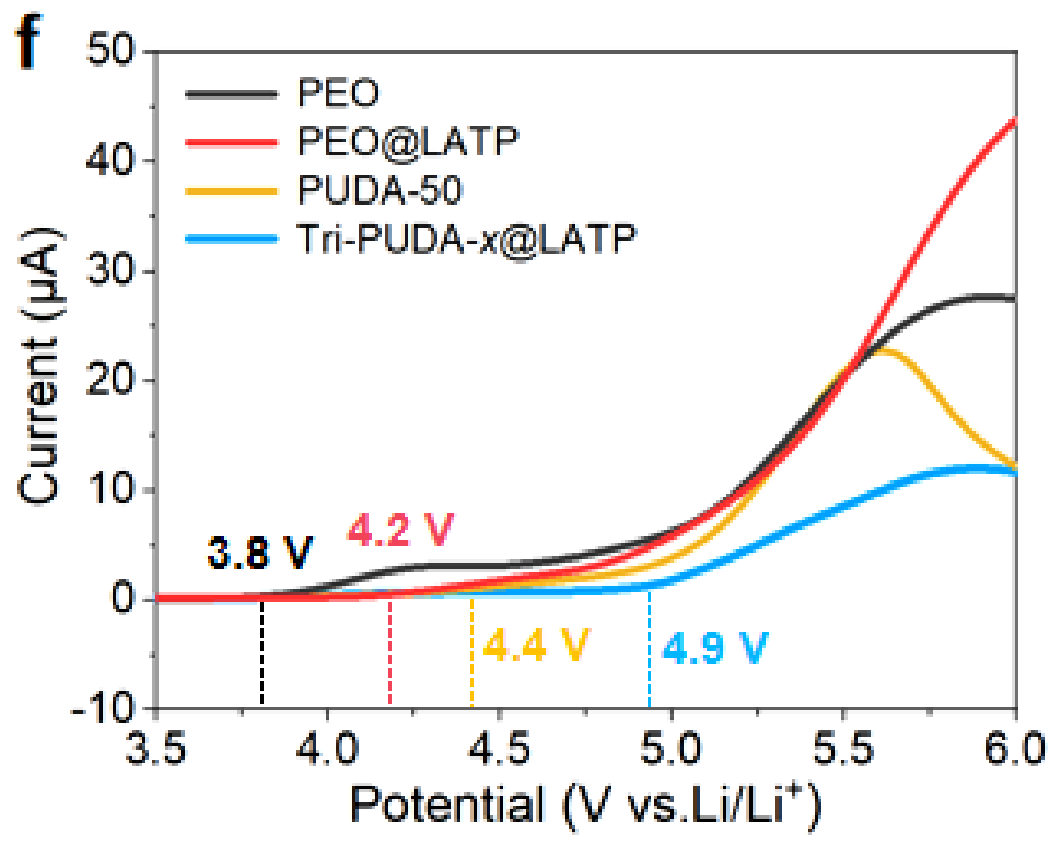

Fig. 3 | f, Linear sweep voltammetry curves comparing electrochemical stability of PEO, PEO@LATP, PUDA-50, and Tri-PUDA-*x*@LATP.

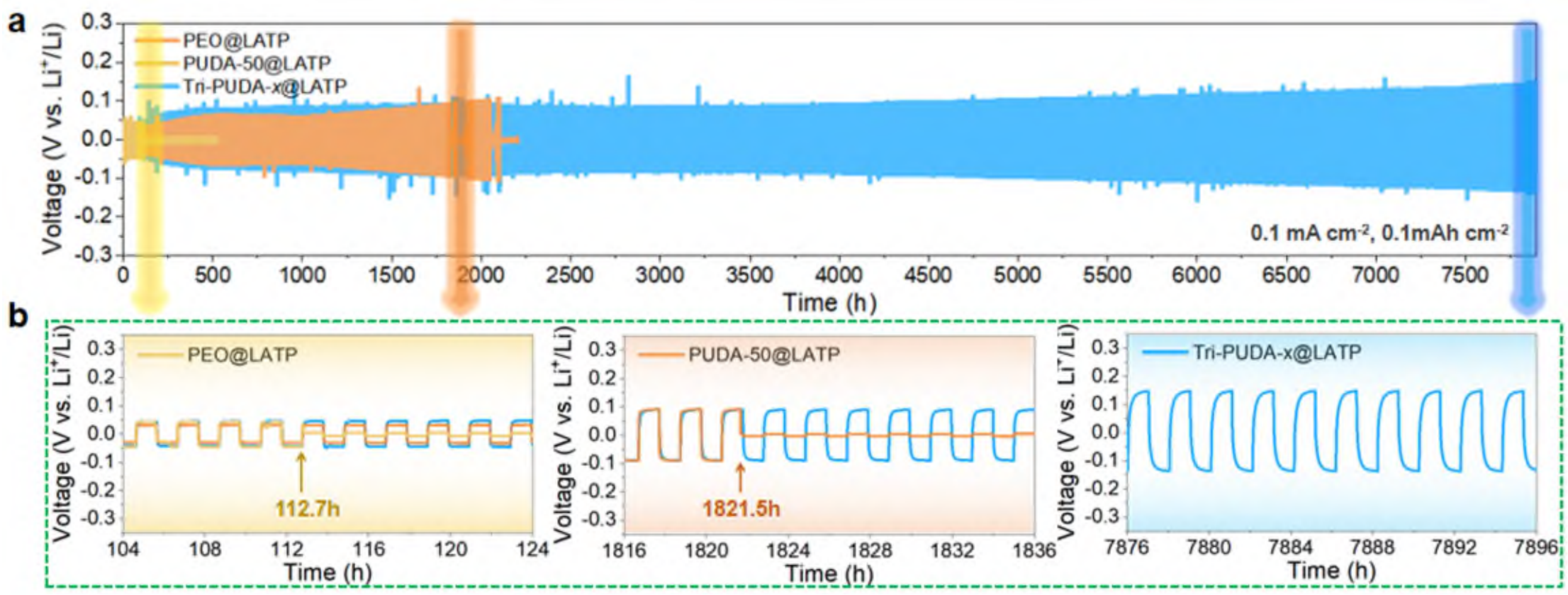

Fig. 4 | a,**b**, Long-term cycling performance of cells with PEO@LATP, PUDA-50@LATP, and Tri-PUDA-*x*@LATP at 0.1 mA cm^{-2}/0.1 mAh cm^{-2}, 60°C.

Revised/Added Text in Main Manuscript:

In Section 2.3, we have added the following paragraph (**Page 19**):

The Li^+ transference number (t_{Li^+}) of the Tri-PUDA-*x*@LATP electrolyte reached 0.605 at 25 °C (**Fig. S12**), significantly higher than that of conventional PEO-based systems (typically <0.3).

In Supporting Information, we have added a new Fig. S12 and the following text:

To elucidate the dominant charge carrier, the Li^+ transference number (t_{Li^+}) of the Tri-PUDA-*x*@LATP electrolyte was determined via potentiostatic polarization

using the Bruce-Vincent method. A constant polarization of 10 mV was applied to a Li|Tri-PUDA-*x*@LATP|Li symmetric cells for 7000s, and the current evolution was recorded to calculate t_{Li^+}.

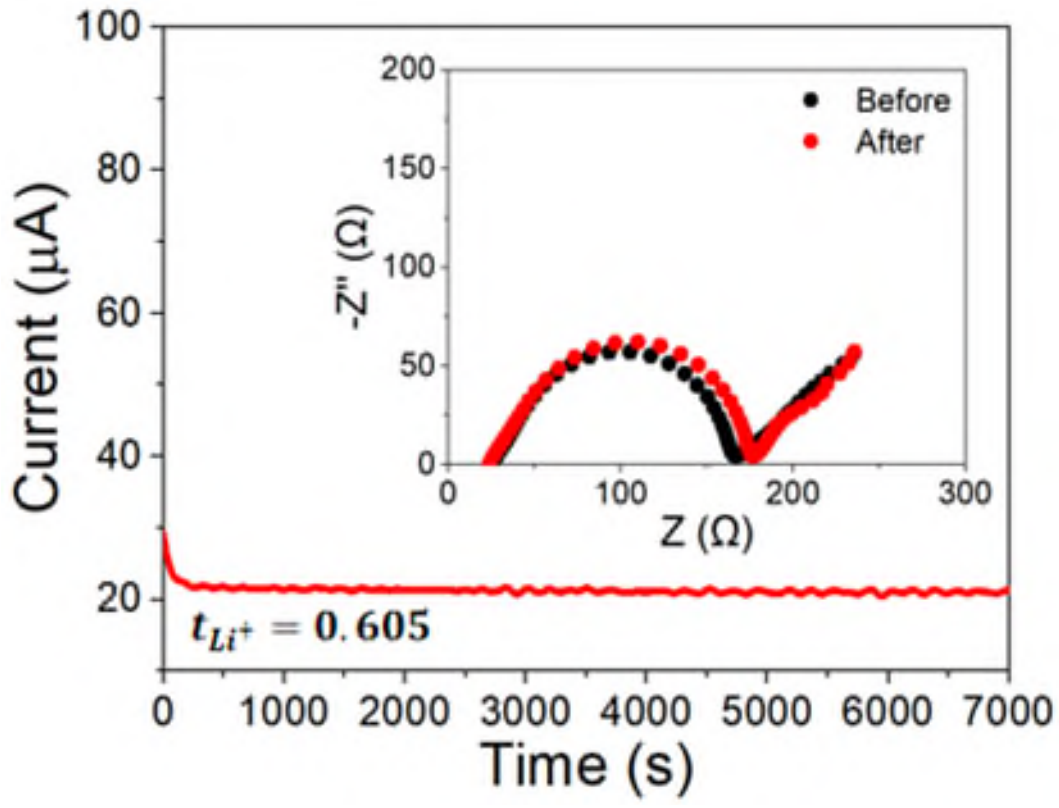

Fig. S12 | Chronoamperometry curve and AC impedance spectra before and after polarization of the Li|Tri-PUDA-*x*@LATP|Li symmetric cell.

5. What is the fundamental reason for the high echem stability? When I looked at the chemistry of the polymer, some of these functional groups are not electrochemically stable against high potential.

Author Reply: We sincerely thank the Reviewer's insightful observation on the origin of the high electrochemical stability. The observed stability up to 4.9 V vs. Li^+/Li does not stem from the synthesis of any single component, but from a synergistic interface engineering strategy enabled by the unique mechano-integrated gradient architecture. The fundamental reason for the high stability is the construction of a ceramic-dominant, dense physical barrier at the cathode interface, a feat made possible by the unprecedented mechanics of the PUDA binder. The key points are detailed below:

(1) Physical barrier effect of the cathode-facing gradient layer:

The cathode-facing layer in our MIGE (PUDA-100) is not a conventional polymer-rich composite but a ceramic-dense membrane. As characterized in Figs. 1c, 2d, and 3h, this layer contains 100 wt% LATP filler relative to the polymer. In this configuration, the LATP particles (>5 V vs. Li^+/Li) form a continuous, percolating network that constitutes the primary electrochemically active interface with the cathode. This architecture minimizes the direct exposure of vulnerable polymer functional groups to the oxidizing cathode surface, with the dense LATP layer serving as a protective physical barrier. The PUDA polymer, due to its ultra-viscoelasticity (>5000% strain), acts exclusively as an ultra-thin, conformal adhesive at the particle boundaries, enabling the fabrication of this dense, crack-free ceramic layer, a structure impossible to achieve with brittle binders like PEO.

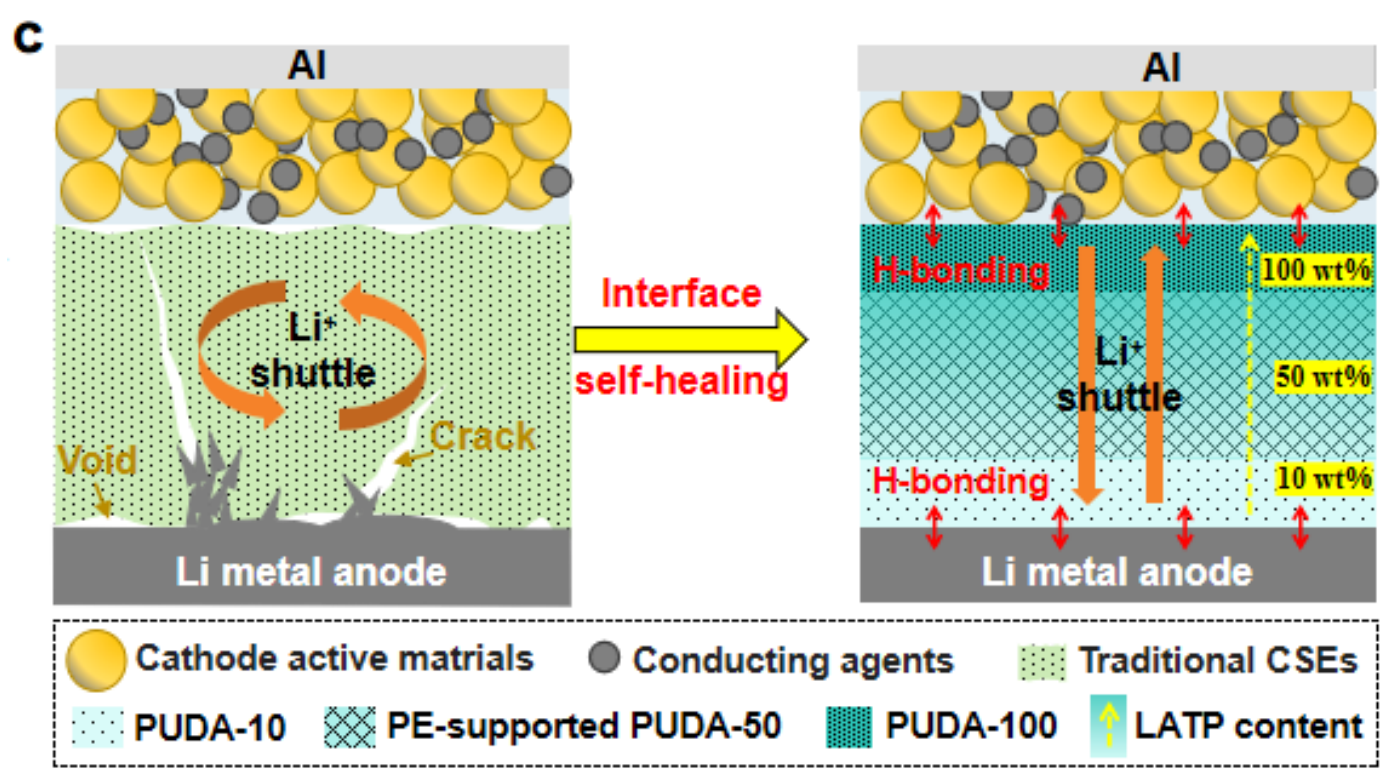

Fig. 1 | c, Structural comparison between conventional laminated and integrated MIGE-based SSLMB configurations.

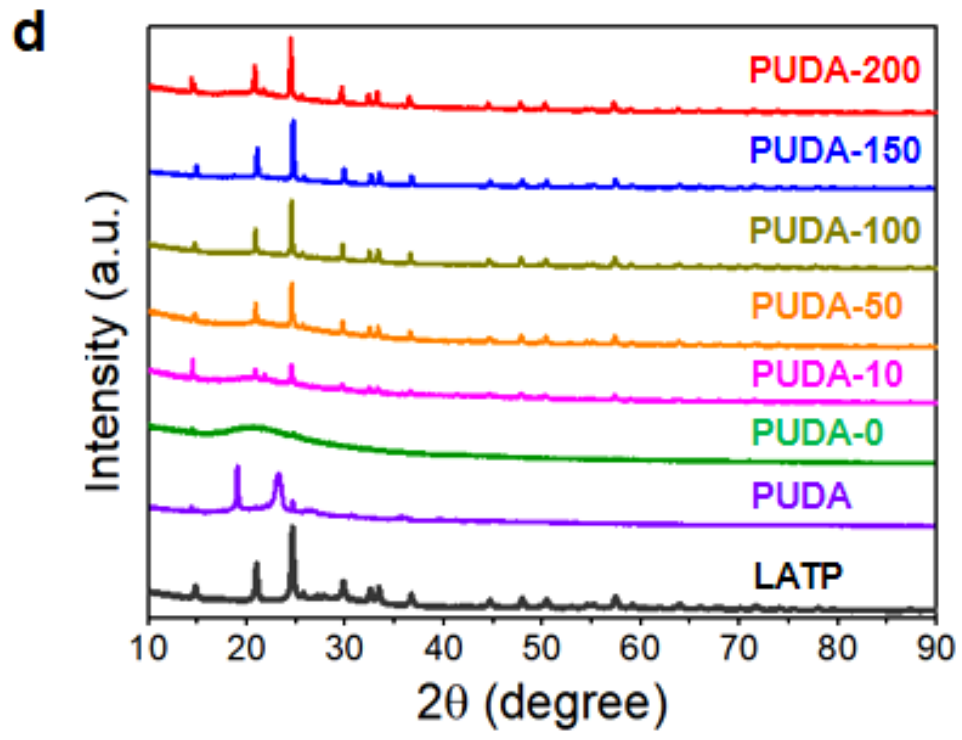

Fig. 2 | d, XRD patterns of LATP, PUDA, and PUDA-*x* composites.

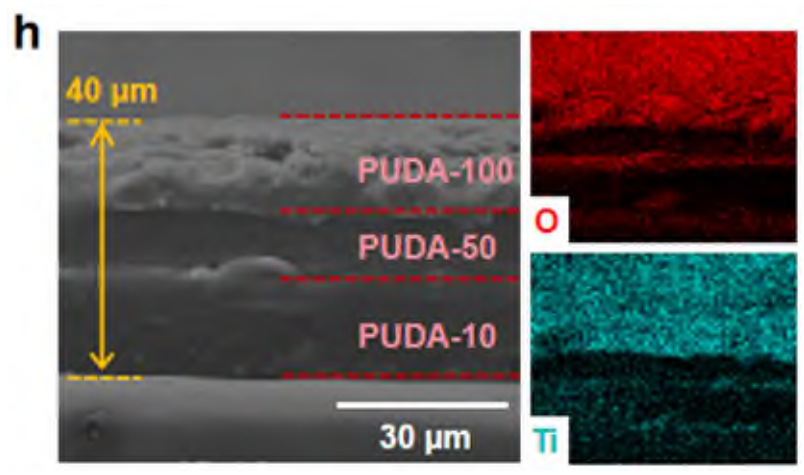

Fig. 3 | h, Cross-sectional SEM image and EDS elemental mapping of Tri-PUDA-*x*@LATP.

(2) Role of compositional and interfacial engineering:

While the dense ceramic layer is the primary stabilizing factor, our design incorporates additional elements for interfacial robustness. A small, optimized amount of [EMIM][TFSI] (10 wt% relative to polymer, 5.77 wt% relative to the total solid content) is included. It is known that its main function is to enhance ionic conductivity, improve interfacial wettability, and contribute to a more stable initial interface formation at the triple-phase boundary, though the dominant stabilizing factor remains the physical barrier described above. In addition, the dense hydrogen-bonding network formed by MDI, DBDA and ADH significantly restricts the segmental motion of polymer chains, particularly within the filler-rich cathode layer. This not only contributes to mechanical robustness but also kinetically hinder the reorganization and approach of susceptible functional groups towards the oxidizing cathode surface. This rationale is consistent with reported strategies (see **Figure 1**) that employ crosslinkers, such as PEG-HMDI (stable up to 4.8 V) and PTMG-HDI

(stable up to 4.1 V), to suppress chain mobility and thereby improve oxidative stability (e.g., ***Adv. Mater. 2024, 36, 2409269. Nat. Commun. 2024, 15, 351*****).**

The Reviewer's concern likely arises from comparing our polymer's functional groups to those in homogeneous PEO-based electrolytes. In such systems, the polymer forms a continuous, exposed film, making its functional groups the primary interface with the cathode, leading to the well-known ~3.8 V limit. Our work introduces a paradigm shift: we bypass the intrinsic chemical limitation of the polymer by re-engineering the interfacial architecture itself. We use the polymer's unprecedented mechanics not as the primary interface, but as an enabling binder to construct a composite where the stable ceramic phase defines the electrochemical interface.

[Figure Redacted]

Figure 1. Linear sweep voltammetry of the previous reported polymer-based solid electrolytes. (a) *Adv. Mater. 2024, 36, 2409269.* (b) *Nat. Commun. 2024, 15, 351*.

To make this crucial point unequivocally clear, we propose the following additions and revisions into the manuscript:

In Section 2.1 (Design), when introducing the gradient layers (**Page 8**):

A cathode-facing layer (PUDA-100, 100 wt% LATP) engineered as a ceramic-dense barrier, where LATP particles forms a continuous protective layer with the PUDA binder serving as a conformal adhesive at particle boundaries.

In Section 2.3, alongside the LSV data (Fig. 3f, **Page 19**):

The enhanced oxidative stability originates from the synergistic combination of a more robust polyurethane backbone[12] and, dominantly, the formation of a dense, ceramic-rich protection layer at the cathode interface. This structural design, enabled by PUDA's unique binder properties, effectively decouples the requirement for intrinsic polymer stability from achieving high-voltage cell operation.

In Section 2.1, under electrolyte preparation (Fig. 1b, **Page 6**):

A controlled amount of 1-ethyl-3-methylimidazolium bis(trifluoromethylsulfonyl)imide ([EMIM][TFSI], 10 wt% relative to the polymer) was added to the precursor solution to enhance interfacial stability and ionic conductivity.

In Section 2.5, alongside the NCM811 data (Fig. 5g, **Page 25**):

This work demonstrates that high-voltage stability in polymer-based composites can be achieved through a mechano-integrated design that controls interfacial architecture. By utilizing an ultra-viscoelastic binder to construct a ceramic-rich protective interface, we physically decouple conventional polymer functionalities from the harsh cathode environment, offering a scalable pathway to high-voltage SSLMBs.

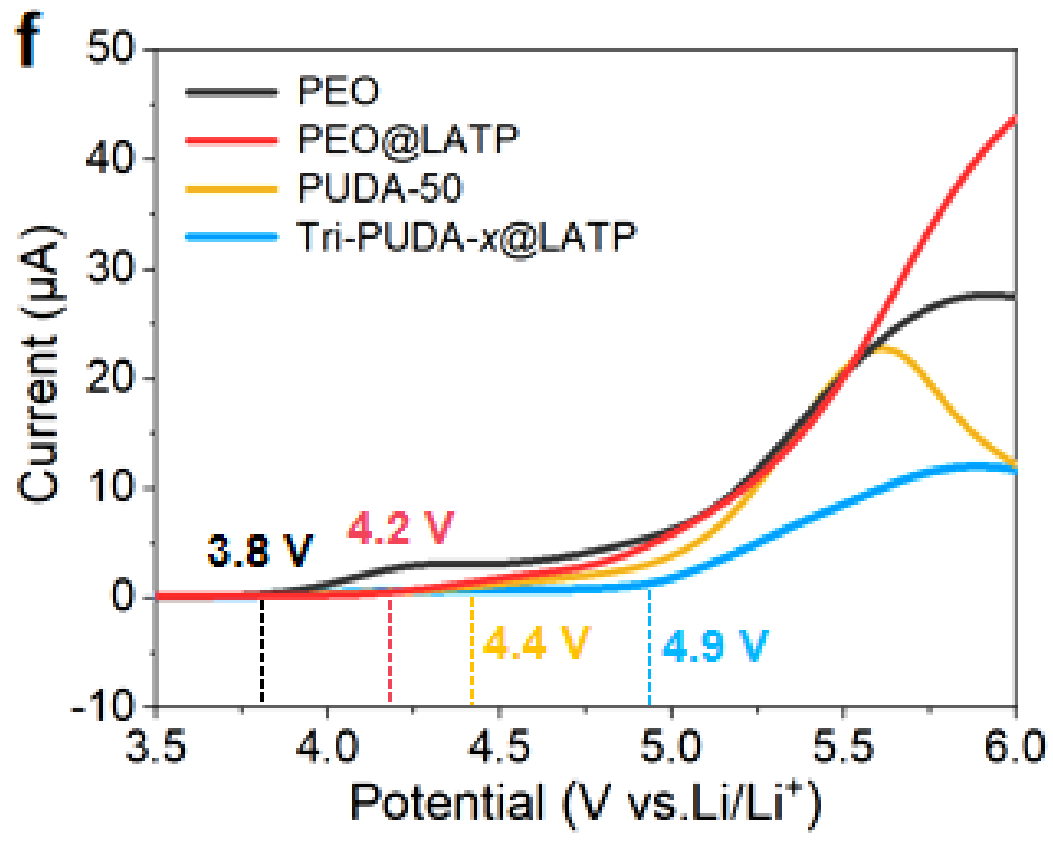

Fig. 3 | f, Linear sweep voltammetry curves comparing electrochemical stability of PEO, PEO@LATP, PUDA-50, and Tri-PUDA-*x*@LATP.

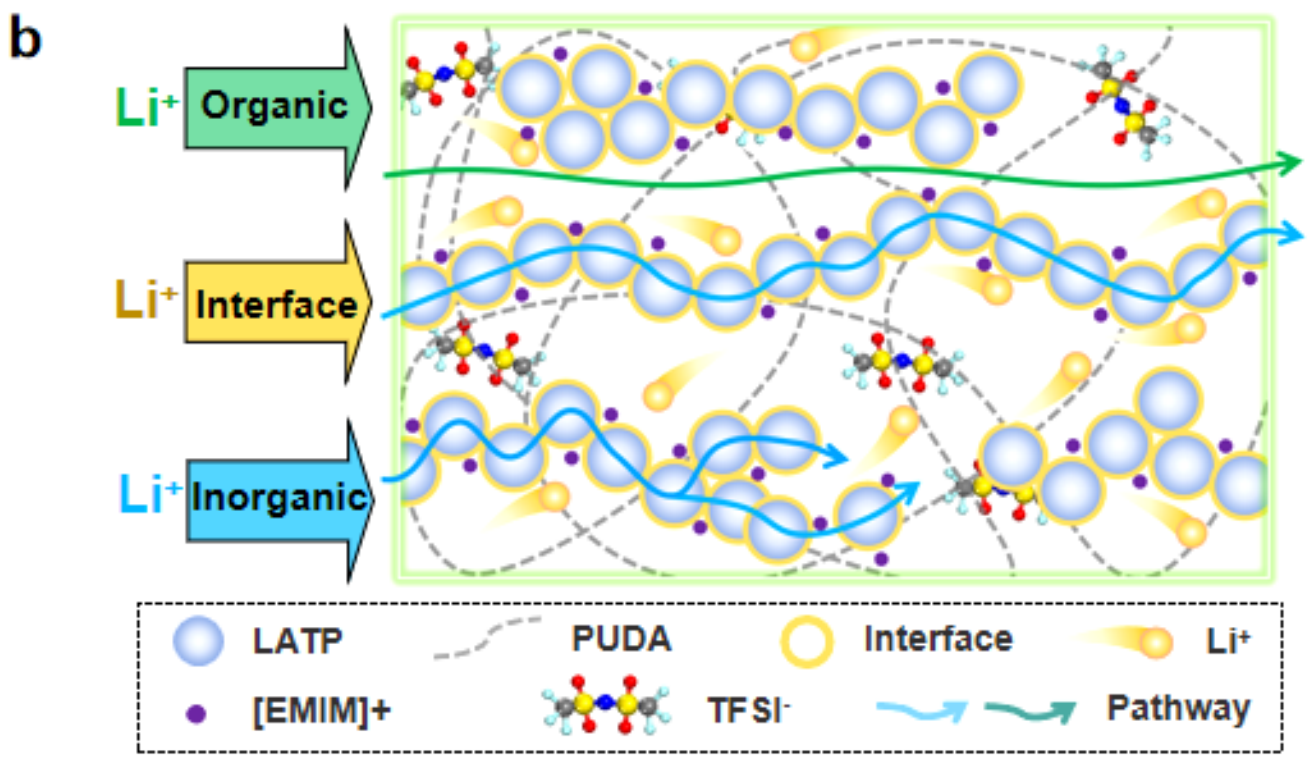

Fig. 1 | b, Schematic of the triphasic ion-transport pathways within the PUDA-based composite electrolyte, including the polymer matrix (dashed gray line), LATP filler (blue circle), and the localized ionic liquid molecules (purple dot) at interfaces aiding particle dispersion.

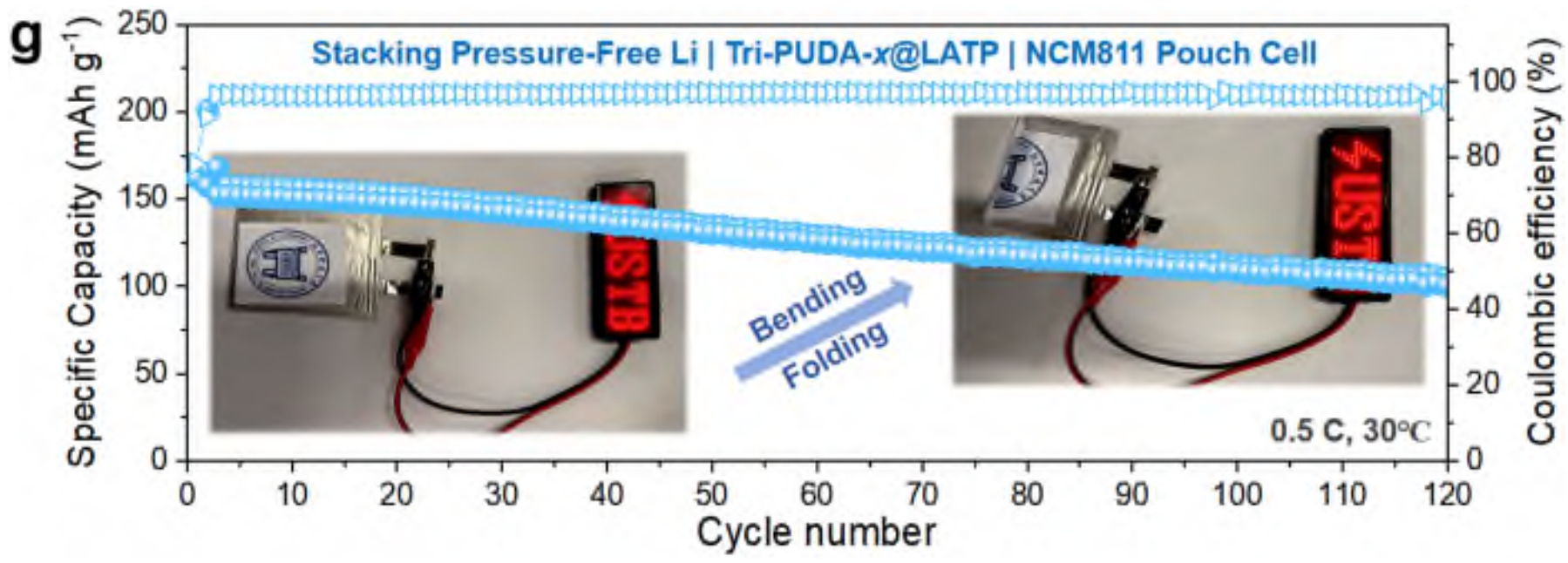

Fig. 5 | g, Cycling stability of solid-state Li|Tri-PUDA-*x*@LATP|NCM811 pouch cell without applied stacking pressure at 0.5C.

Reviewer #2: *The manuscript reports a mechano-integrated gradient electrolyte using a highly viscoelastic polymer matrix and LATP solid electrolyte fillers dispersed in the polymer matrix in a graded manner. The authors highlight the advantages of their design: the composite with a high LATP content is placed at the cathode side to improve oxidative stability, the composite with low LATP content remains soft to*

maintain good interfacial contact with the lithium metal anode, and the composite with intermediate LATP content provides sufficient ionic conduction in the bulk. The overall advancement toward solvent-less composite solid electrolytes is appreciated, especially considering the minimal weight loss at a high temperature of 200 ℃. However, the use of EMIM–TFSI ionic liquid should be clearly declared in the main manuscript, and the claimed ion conduction across the organic–inorganic interface requires further justification, given the widely recognized large interfacial resistance between organic and inorganic phases. Therefore, some details and explanations are needed before publication.

Author Reply: We sincerely thank Reviewer #2 for the positive evaluation of our "mechano-integrated" gradient design and for recognizing its advantages in oxidative stability and high-temperature robustness. We have carefully addressed the points regarding the declaration of ionic liquids and the organic–inorganic interfacial resistance, with detailed responses and supporting data provided below.

1. The author claimed that the asymmetric interfacial demands of lithium metal anodes and high-voltage cathodes remain unmet: the former requires soft, adaptive interfaces to maintain contact during cycling, while the latter necessitates rigid, dense barriers against oxidation. While this understanding is generally appreciated, the description may not be sufficiently accurate. Both anode and cathode interfaces benefit from soft and adaptive contact. Cathode materials are typically oxide-based, and rigid contact is insufficient to ensure good ion conduction, which is a known challenge for oxide-based solid electrolytes on the cathode side. In this work, the ceramic-enriched electrolyte at the cathode appears to primarily improve oxidative stability rather than mechanical rigidity, which should be clarified to avoid confusion.

Author Reply: We agree with the Reviewer that both interfaces indeed require a degree of conformal contact to ensure efficient ion transport. Our intention was to highlight the different priorities at each interface: At the anode, the dominant challenge is the large, dynamic volume change during plating/stripping, which demands extreme deformability (PUDA-10 strain ~4853%) to maintain continuous contact. At the cathode, the priority is to provide a dense, pinhole-free barrier against oxidative decomposition at high voltage (>4.5 V), while still preserving sufficient interfacial contact (**PUDA-100 strain ~3264%, Fig. 2g**) through the viscoelastic polymer matrix.

In our MIGE architecture, the cathode-side layer (PUDA-100) is a ceramic-rich composite rather than a pure ceramic. The PUDA matrix remains continuous, providing the necessary viscoelasticity to ensure adequate contact with cathode particles, while the high filler loading dramatically reduces oxygen permeability and raises the oxidative stability limit. We have revised the text to avoid the term “rigid” and instead emphasize “a dense, ceramic-rich yet polymer-bonded interface” that combines oxidation resistance with maintained interfacial conformity.

Revised/Added Text in Main Manuscript:

Section 1 (Introduction) – revised to (**Page 4**):

Critically, the lithium metal anode requires an electrolyte with extreme deformability and adaptive viscoelasticity to maintain conformal contact throughout repeated plating/stripping cycles, whereas the high-voltage cathode demands a dense, pinhole-free, and ceramic-rich barrier against oxidation and transition-metal dissolution, while still preserving sufficient interfacial contact through the continuous polymer phase.

Section 2.1 (description of the cathode layer, **Page 15**) :

The cathode-facing layer (PUDA-100, 100 wt% LATP) forms a ceramic-rich yet polymer-bonded composite that serves as a dense oxygen barrier while retaining substantial deformability (~3264% strain). Even at 200 wt% LATP, where incipient agglomeration reduces tensile strength to 1.32 MPa, elongation remains exceptional (~1477%), far surpassing conventional PEO-based electrolytes[19,31].

Section 3 (Conclusion) – refined wording (**Page 30**):

By spatially grading the LATP content, we decouple the interfacial priorities: ultra-conformal, strain-tolerant contact at the anode and a dense, oxidation-resistant yet still conformal interface at the cathode.

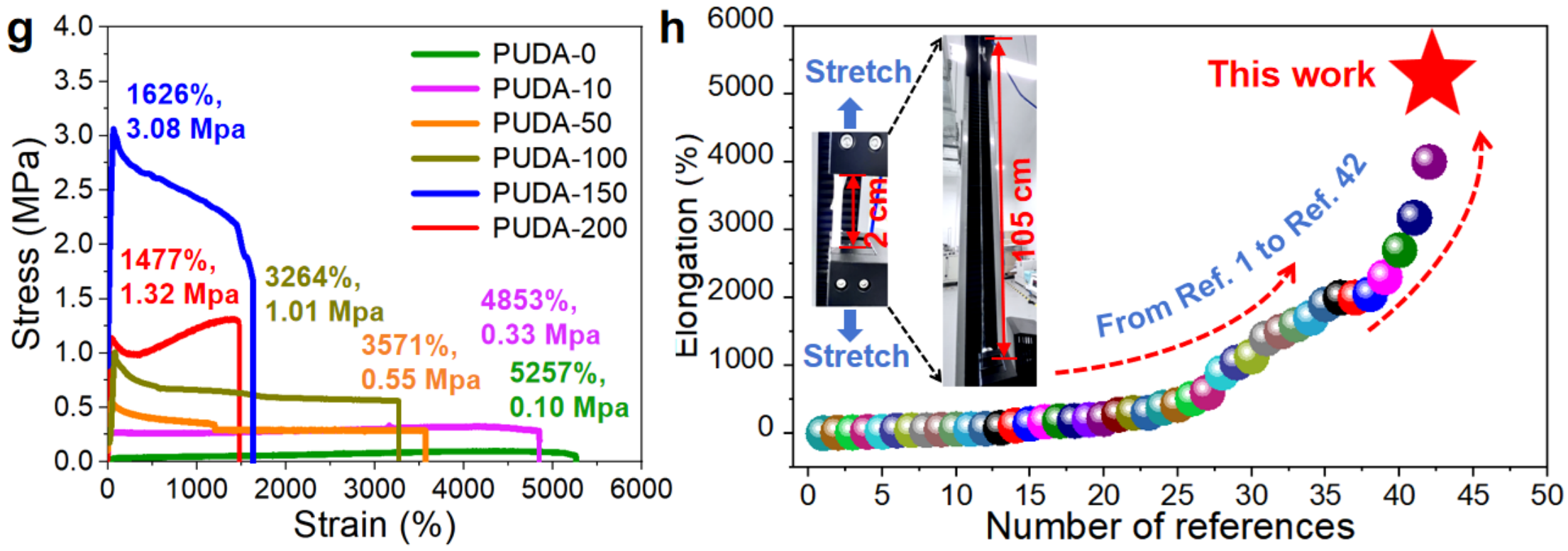

Fig. 2 | g, Stress-strain curves of PUDA-*x* composites. The filler loading *x* is defined as the weight percentage of LATP relative to the PUDA polymer matrix. **h**, Elongation at break (fracture strain) comparison between PUDA-0 and previously reported polymer electrolytes.

2. The formation process of the mechano-integrated gradient electrolyte is not clearly described. After checking both the main manuscript and the Supporting Information, no clear description of how the spatial ceramic gradient is formed can be found. It only becomes apparent from the final figure that the structure is stacked layer by layer. This important fabrication detail should be clarified earlier.

Author Reply: We sincerely thank the Reviewer for this insightful and constructive comment and apologize for the initial lack of clarity regarding the fabrication process of the mechano-integrated gradient electrolyte. It has been added now, which helped us significantly improve the clarity and completeness of the manuscript. As below, we

provide a detailed explanation of the fabrication strategy and specify the corresponding modifications we have made to the manuscript.
The spatial gradient of LATP fillers is achieved through a sequential multilayer slot-die coating process, enabled by the ultra-viscoelastic and strongly adhesive nature of the PUDA matrix. Because PUDA exhibits a fracture strain >5000% and can bind >200 wt% of inorganic particles without cracking, it allows the deposition of distinct layers with sharply differing filler contents (10 wt%, 50 wt%, and 100 wt% LATP) while maintaining interfacial cohesion and mechanical integrity throughout the coating and drying steps.

To address the Reviewer's concern, we have added the following clarifications in the revised manuscript and Supporting Information:

In the Supporting Information, we have expanded the description of the formation process, specifying coating parameters, drying conditions, and the equipment used.

The formation process of the MIGE comprises the following key steps:

1) Unwinding: A porous PE substrate (12 μm thick) is fed from a roll.

2) Front-side coating (ion-transport layer): A slurry containing PUDA-50 (50 wt% LATP) is uniformly coated onto one side of the PE via slot-die coating, followed by low-temperature drying (60-80 °C) and mild hot-pressing to ensure adhesion and eliminate pores.

3) Front-side coating (anode interface layer): A second slot-die coater deposits PUDA-10 (10 wt% LATP) directly onto the dried PUDA-50 layer, again followed by drying and hot-pressing. This yields a smooth, gradient interface between the two layers.

4) Reverse-side coating (cathode interface and symmetric ion-transport layer): The web is flipped, and two separate slot-die heads simultaneously coat PUDA-100 (100 wt% LATP) and another PUDA-50 layer onto the backside of the PE substrate. The high filler content (100 wt%) slurry forms a dense, ceramic-rich cathode-facing barrier, while the adjacent PUDA-50 ensures symmetric ion-transport pathways.

5) Calendering and winding: The coated web passes through a calendering unit to control total electrolyte thickness (~40 μm) and is then wound into master rolls. Protective release liners are applied on both sides to handle the tacky surface.

6) Transfer-printing assembly: The MIGE film is cut and laminated with Li metal anodes and cathodes (e.g., LFP or NCM811) via a solvent-free transfer-printing technique under minimal pressure, completing the cell assembly.

In Section 2.1, after describing the triphasic model, we now include a concise paragraph outlining the coating sequence (**Page 7**):

Compatible with roll-to-roll manufacturing, the MIGE is formed by sequential coating the functional layers onto an ultrathin (~12 μm), porous polyethylene (PE) substrate (see Supporting Information for details).

3. The authors claim that the organic–inorganic interphase facilitates Li^+ transport.

However, it is widely recognized that the interfacial ion transfer resistance between organic and inorganic materials is typically large. Although many composite electrolytes have been reported, their performance improvement is often limited. Fundamental studies (e.g., ACS Omega 2021, 6, 24, 16187–16193) have shown that large interfacial resistance results in limited contribution of LATP particles to Li-ion conduction, with most ions traveling through either the polymer or the LATP phase. Therefore, additional evidence is required to support this claim.

Author Reply: We thank the Reviewer for raising this fundamental and critical point regarding the role of the organic–inorganic interphase in composite solid electrolytes (CSEs). We fully agree that in many conventional CSEs, poor interfacial contact and high impedance between the polymer matrix and ceramic filler severely limit the filler's contribution to ion transport, as exemplified by the PVDF-HFP/LATP system cited (*ACS Omega 2021, 6, 16187–16193*). This widespread challenge underscores the necessity of deliberate interface engineering, which our work directly addresses.

The cited ACS Omega study uses a PVDF-HFP gel containing a high concentration of liquid plasticizer (sulfolane solvent, SL). The weight ratio of PVDF−HFP/[Li(SL)$_2$]-[TFSA] is 30:70. In such systems, the polymer's role is largely structural, and the interface with LATP is primarily physical. This often leads to poor wetting, high interfacial resistance, and limited contribution from the filler—consistent with the Reviewer's concern. By contrast, in our Tri-PUDA-*x*@LATP system, we engineer both the polymer matrix and the interface to mitigate this resistance. Below, we address the Reviewer's concern through the following points: The PUDA matrix is not a passive binder but a dynamic hydrogen-bonded network engineered with dual extenders (DBDA and ADH). This provides exceptional viscoelasticity (>5000% strain) and strong adhesive capability, promoting intimate, defect-minimized contact with LATP particles. We incorporate a moderate amount of ionic liquid (PUDA:[EMIM][TFSI]=10:1), which preferentially localizes at the polymer-filler interface, plasticizing the boundary region and enhancing local Li^+ mobility. At the optimal filler loading (50 wt% LATP), a continuous percolation network is formed. Crucially, the low-impedance interface we engineer enables this network to function effectively, allowing Li^+ to hop between LATP particles via the interfacial regions rather than being confined to a single phase.

A growing body of literature confirms that a well-engineered organic–inorganic interface can indeed enhance ion transport, contrary to the conventional view of it being always resistive. These studies underscore that interface properties are system-dependent, and strategic material design can transform the interface from a barrier into a facilitator. For example (see **Figure 1**):

[Figure Redacted]

Figure 1. (a) *Adv. Mater. 2024, 36, 2409269.* (b) *ACS Appl. Mater. Interfaces 2024, 16, 37, 48706–48712.* (c) *ACS Appl. Energy Mater. 2019, 2, 1452−1459.* (d) *ACS Appl. Mater. Interfaces 2018, 10, 4113−4120.* (e) *Energy Storage Mater. 2024, 65, 103091.* (f) *Adv. Mater. 2024, 2403078.*

Taking into account the suggestions of the Reviewer, we have toned down the definitive language regarding the interphase's role. Instead of claiming it "facilitates" transport unconditionally, we now state:

In Section 2.1 (Molecular Design), we have added the following clarification where the triphasic transport model is introduced (**Page 7**):

The strong adhesive nature of the PUDA matrix, combined with interfacial plasticization by the ionic liquid, is designed to minimize the Li^+ migration barrier at the organic–inorganic boundary. This engineered interphase, coupled with the formation of a percolated LATP network, enables efficient Li^+ transport throughout the multilayer gradient structure.

In Section 2.3 (Electrochemical Performance), the conclusion regarding the conductivity mechanism has been refined to (**Page 18**):

The high ionic conductivity achieved at the 50 wt% percolation threshold arises not only from the continuous PUDA and LATP network but also from a minimized interfacial impedance. This is attributed to the PUDA matrix's exceptional adhesion and the interface-active role of the ionic liquid, which collectively facilitate Li^+ exchange between phases, a critical advancement over conventional high-resistance composite interfaces.

4. EMIM–TFSI ionic liquid is used as a core component in this composite electrolyte and may significantly contribute to the observed ionic conductivity. However, this information is only disclosed in the Supporting Information. The authors should clearly state and discuss its role in the main text. Accordingly, the schematic in Fig. 1b should be revised. The term "all-solid-state" may not be appropriate, as EMIM–TFSI is liquid at room temperature.

Author Reply: We sincerely thank the Reviewer for this insightful and important comment. The [EMIM][TFSI] was introduced in a very low mass ratio (10 wt% relative to the PUDA polymer, 5.77 wt% relative to the total solid content) primarily to improve interfacial wetting and to assist in the dispersion of high-loading inorganic fillers during solution processing.

In prior research, the terms "solid-state," "semi-solid," "all-solid-state," and "quasi-solid" have often been used interchangeably, and there is currently no internationally unified standard for defining "all-solid-state" batteries. Although the final Tri-PUDA-*x*@LATP electrolyte film is a self-standing, solvent-free, and non-flowable solid with a solid content exceeding 95 wt%. After being held under vacuum at 120 °C for 6 h, the mass loss was less than 0.3%, which meets the criteria outlined in the **"Assessment Method for All-Solid-State Batteries" proposed by China in 2025**. Furthermore, almost no significant mass loss (<1%) was observed at 200 °C. Nonetheless, to prevent any potential misunderstanding and to adhere to more rigorous terminology, we have replaced the term "all-solid-state" with "solid-state" throughout the revised manuscript.

Revised/Added Text in Main Manuscript:

We have added the following explanation in Section 2.1 (**Page 6**):

A controlled amount of 1-ethyl-3-methylimidazolium bis(trifluoromethylsulfonyl)imide ([EMIM][TFSI], 10 wt% relative to the polymer) was added to the precursor solution to enhance interfacial stability and ionic conductivity.

Furthermore, in Section 2.3, we have added (**Page 18**):

The high ionic conductivity achieved at the 50 wt% percolation threshold arises not only from the continuous PUDA and LATP network but also from a minimized interfacial impedance. This is attributed to the PUDA matrix's exceptional adhesion and the interface-active role of the ionic liquid, which collectively facilitate Li^+ exchange between phases, a critical advancement over conventional high-resistance composite interfaces.

We have revised Fig. 1b to include a representative fraction of ionic liquid molecules at the organic–inorganic interface. The revised caption now reads (**Page 9**):

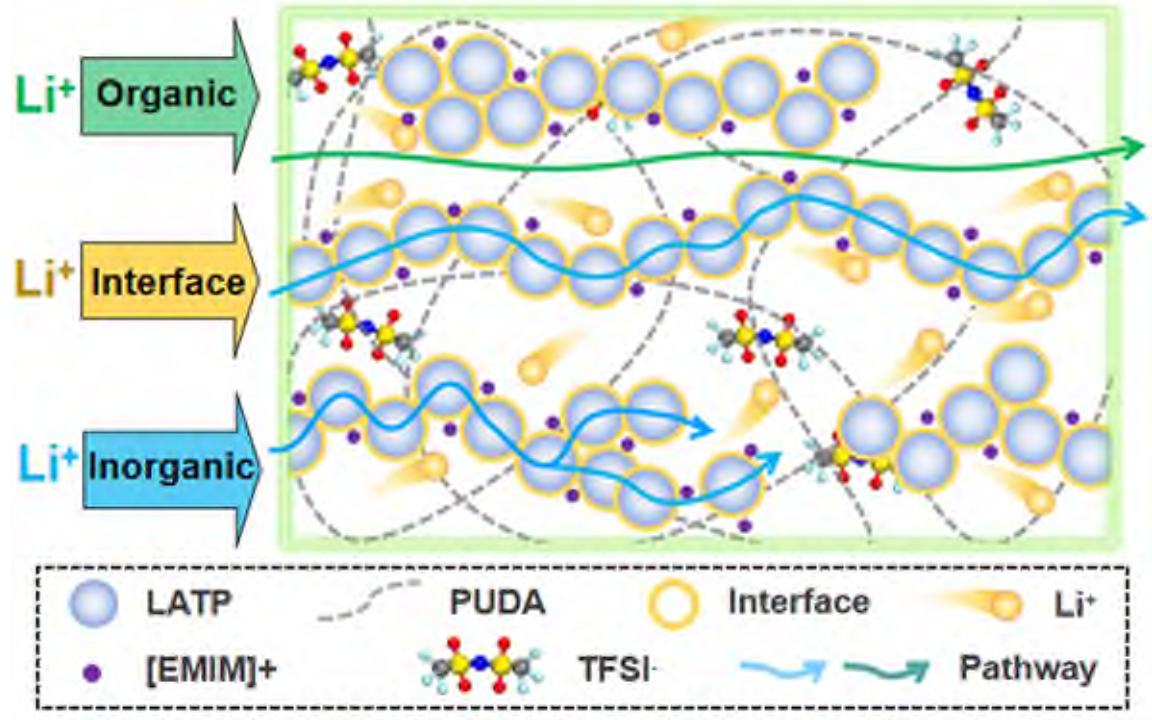

Fig. 1 | b, Schematic of the triphasic ion-transport pathways within the PUDA-based composite electrolyte, including the polymer matrix (dashed gray line), LATP filler (blue circle), and the localized ionic liquid molecules (purple dot) at interfaces aiding particle dispersion.

5. Fig. 2e requires further explanation of the blue and yellow regions.

Author Reply: We sincerely thank the Reviewer for this insightful comment, which allows us to clarify an important aspect of our microstructure-property analysis. The color-coded regions in Fig. 2e werre derived from image analysis of SEM micrographs and represent distinct microstructural states. As the Reviewer rightly highlighted, the blue regions correspond to interconnected inorganic (LATP) particle pathways. These networks provide low-resistance highways for Li^+ transport, leveraging the intrinsic high ionic conductivity of the ceramic filler. This state is optimally achieved at intermediate filler loadings (~50 wt%), where the polymer matrix's ultra-viscoelasticity maintains particle separation and prevents agglomeration, thereby enabling the formation of a triphasic (polymer/ceramic/interphase) conductive continuum. In contrast, the yellow regions signify areas of filler agglomeration and polymer-phase compression. At high filler contents (~100 wt%), despite the

exceptional adhesive property of PUDA, the increased particle-particle contact probability leads to local agglomeration. This not only disrupts the continuity of the ceramic network but also compresses the polymer-rich domains, which are essential for stress dissipation. Consequently, these regions exhibit increased tortuosity and reduced effective ionic conductivity, as corroborated by the conductivity drop observed for PUDA-100 (Fig. 3d). This phenomenon is more obvious in PUDA-150 and PUDA-200 (Figs. S6, S7).

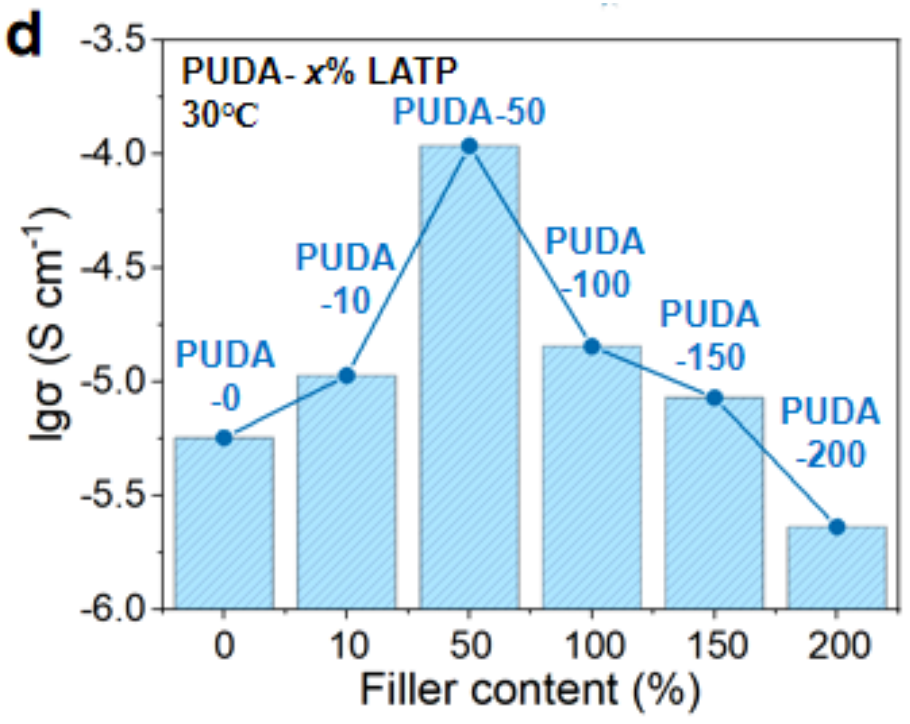

Fig. 3 | d, Ionic conductivity of PUDA-*x* electrolytes as a function of LATP content. **e**, Arrhenius plots of PEO, PUDA, PEO@LATP, and Tri-PUDA-*x*@LATP.

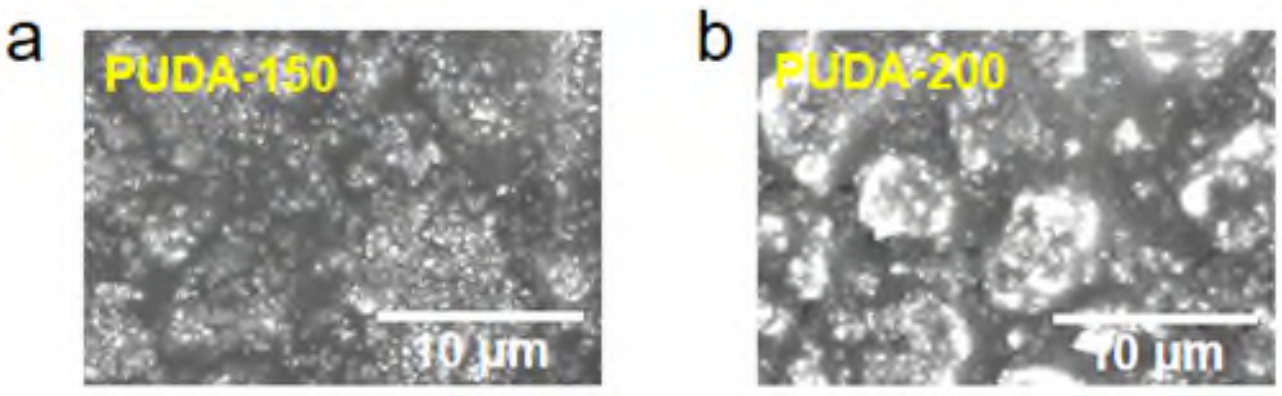

Fig. S6 | Surface SEM images of PUDA-*x* (*x* = 150 and 200).

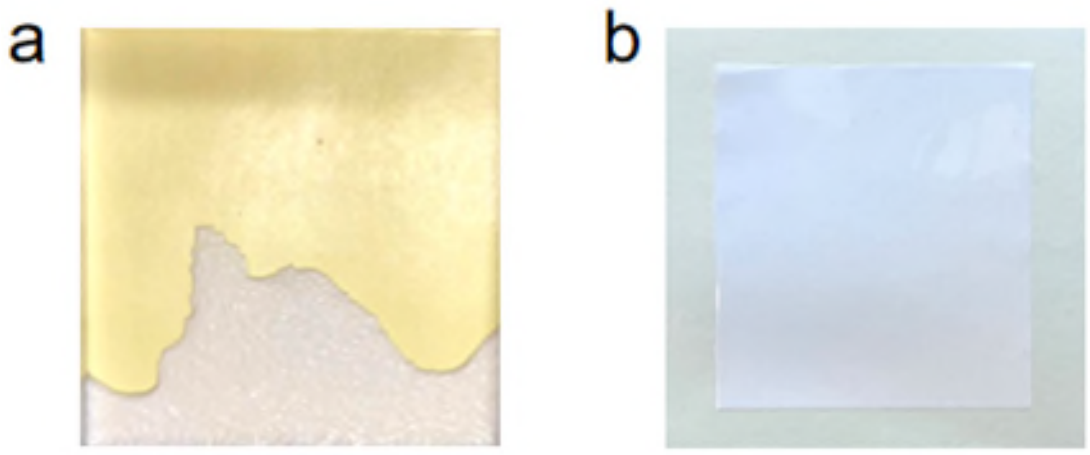

Fig. S7 | Comparison of the film-forming ability of PEO-100 and PUDA-200.

To make this interpretation clear and quantitative, we have revised the caption of Fig. 2e and added a dedicated paragraph in Section 2.2

Revised Fig. 2e caption (**Page 16**):

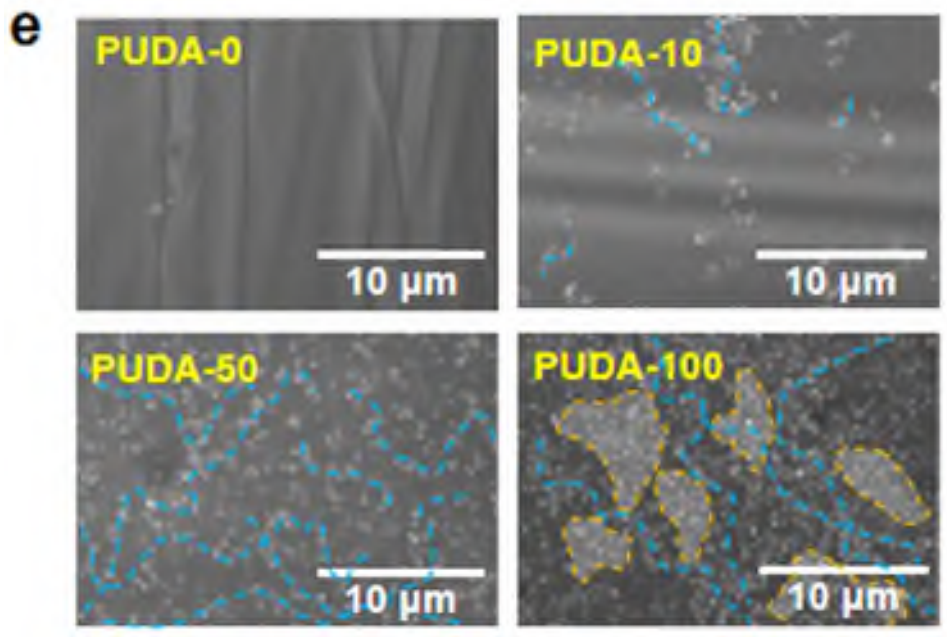

Fig. 2 | e, Surface SEM images of PUDA-*x* electrolytes. Blue regions denote interconnected LATP networks; yellow regions indicate agglomerated filler clusters.

Added Text in Section 2.2 (after SEM description, **Page 13**):

Image analysis of the SEM micrographs (**Fig. 2**e) reveals a spatially heterogeneous microstructure with two distinct regions: interconnected LATP networks (blue) that provide continuous pathways for Li^+ transport, and isolated filler agglomerates (yellow) where particle clustering and polymer-phase compression increase ionic-transport tortuosity. This agglomeration becomes more pronounced at higher LATP loadings of 150 wt% and 200 wt% (**Fig. S6**). Notably, even at 200 wt%, the PUDA-200 film remains crack-free, whereas PEO-based counterparts fail to form coherent films beyond 100 wt% (**Fig. S7**).

6. PUDA-10 and PUDA-100 exhibit ionic conductivities that are approximately one order of magnitude lower. Could this lead to insufficient ion transport near the electrodes, particularly at the cathode side? It is unclear whether these regions could limit the overall cell performance.

Author Reply: We sincerely thank the Reviewer for raising this important point regarding the ionic conductivity of the functional layers within our MIGE architecture. We fully agree that sufficient ion transport across the entire electrolyte is critical for cell performance. Our design philosophy and the resulting electrochemical data demonstrate that the slightly lower conductivity in the electrode-adjacent layers is a deliberate and optimal trade-off, and does not limit overall cell performance for the following reasons:

(1) The Reviewer correctly notes the bulk ionic conductivity of individual layers: PUDA-10 (~1.01 × 10^{-5} S cm^{-1}) and PUDA-100 (~1.43 × 10^{-5} S cm^{-1}) are lower than that of PUDA-50 (~1.09 × 10^{-4} S cm^{-1}). However, the effective ionic conductivity of the integrated Tri-PUDA-*x*@LATP (MIGE) electrolyte is 4.12 × 10^{-5} S cm^{-1} at 30°C (Fig. 3d), with a remarkably low activation energy of 0.24 eV (Fig. 3e). This value, only ~2.6 times lower than that of the best-conducting mid-layer, governs the total cell impedance and is highly competitive among dry-processed polymer-ceramic composites. The low activation energy signifies favorable transport kinetics throughout the integrated structure.

(2) The MIGE architecture is fundamentally designed to decouple and prioritize functions at each interface, which we identify as the more critical failure mode in long-cycling SSLMBs than maximizing bulk conductivity in every region. The

primary function of anode side (PUDA-10) is to provide atomically conformal, dynamically adaptive contact with the Li metal (~4853% strain), accommodating volume changes and suppressing void formation. The moderate conductivity here is a calculated trade-off for achieving this essential interfacial compliance. The primary role of cathode side (PUDA-100) is to act as a dense, pinhole-free oxygen barrier, providing exceptional anodic stability (>4.9 V, Fig. 3f) for high-voltage cathodes like NCM811 (Fig. 5g). The high ceramic content required for this stability inherently increases tortuosity but is paramount for cycle life.

(3) Crucially, as we highlighted, all three layers share an identical, homologous PUDA polymer matrix. During the final hot-pressing stage of our manufacturing process, these layers fuse into a monolithic, single-phase structure. This eliminates the sharp internal interfaces and associated ion-migration barriers that plague heterogeneous multilayer systems. Consequently, Li^+ traverse the electrolyte thickness without encountering sudden energy jumps, making the effective conductivity of the integrated whole the relevant metric, not the conductivity of individual layers in isolation.

The outstanding cell performance presented in the manuscript provides direct evidence that ion transport is not a limiting factor. The Li/LFP maintains excellent capacity retention up to 5C (118.5 mAh g^{-1}) and recovers fully upon returning to 0.1C (Fig. 5c). This would not be possible if the electrode-adjacent layers were severe ionic transport bottlenecks. The Li/LFP cell shows a very low polarization voltage (~0.117 V) at 0.5C, with a minimal fade rate of 0.026% per cycle (Fig. 5a, b) over 1000 cycles. Significant ionic blockage at the interfaces would manifest as a rapidly increasing polarization, which we do not observe. The operation of Li|NCM811 cell (Fig. 5g) further confirms that the ion supply through the cathode-side layer is sufficient for stable cycling.

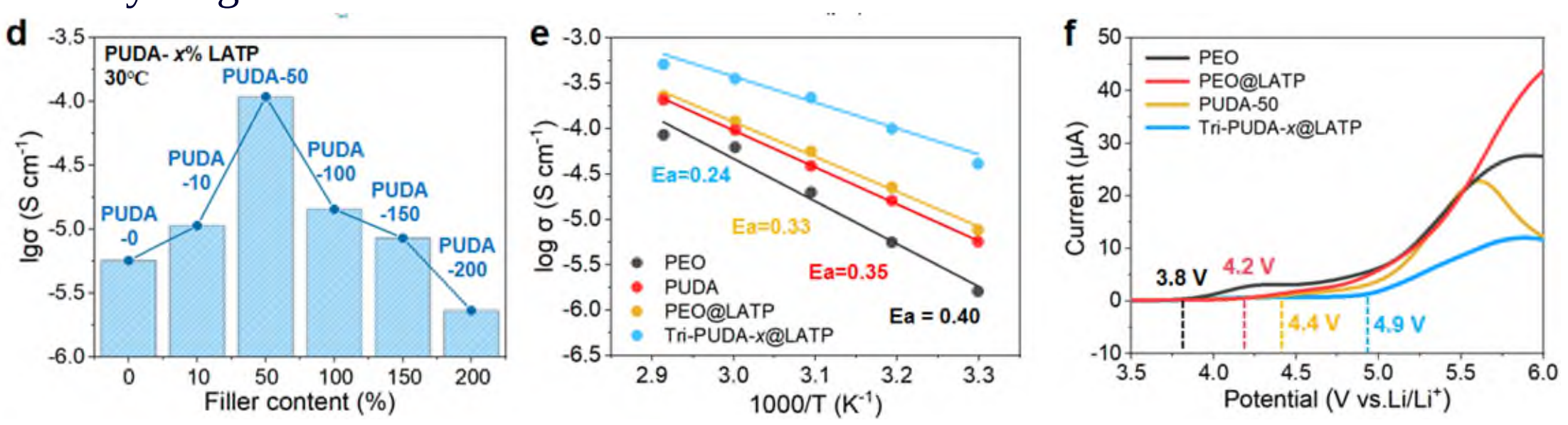

Fig. 3 | d, Ionic conductivity of PUDA-*x* electrolytes as a function of LATP content. **e**, Arrhenius plots of PEO, PUDA, PEO@LATP, and Tri-PUDA-*x*@LATP. **f**, Linear sweep voltammetry curves comparing electrochemical stability of PEO, PEO@LATP, PUDA-50, and Tri-PUDA-*x*@LATP.

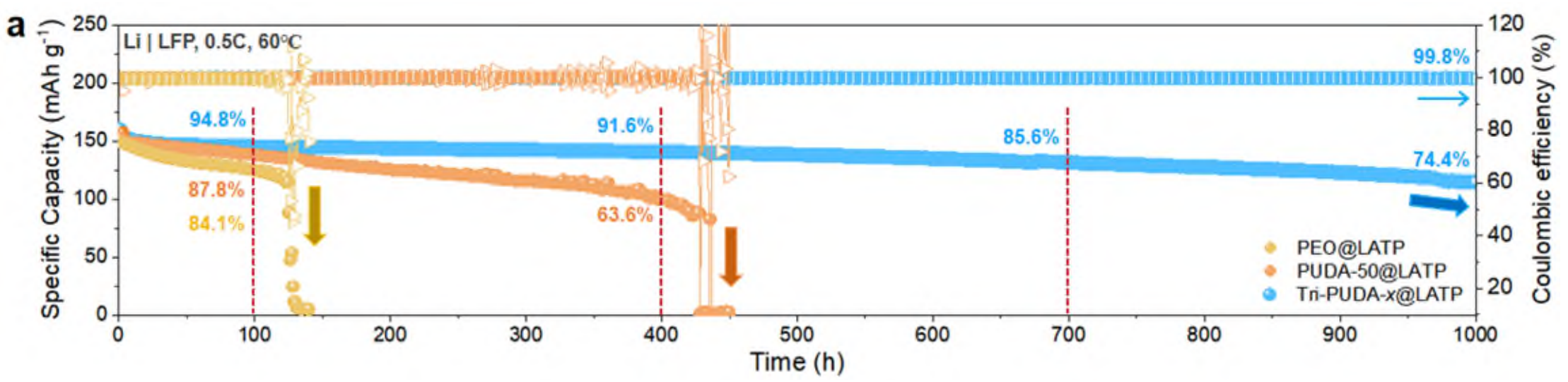

Fig. 5 | a, Long-term cycling performance of solid-state Li|LFP cells with PEO@LATP, PUDA-50@LATP, and Tri-PUDA-*x*@LATP at 0.5C.

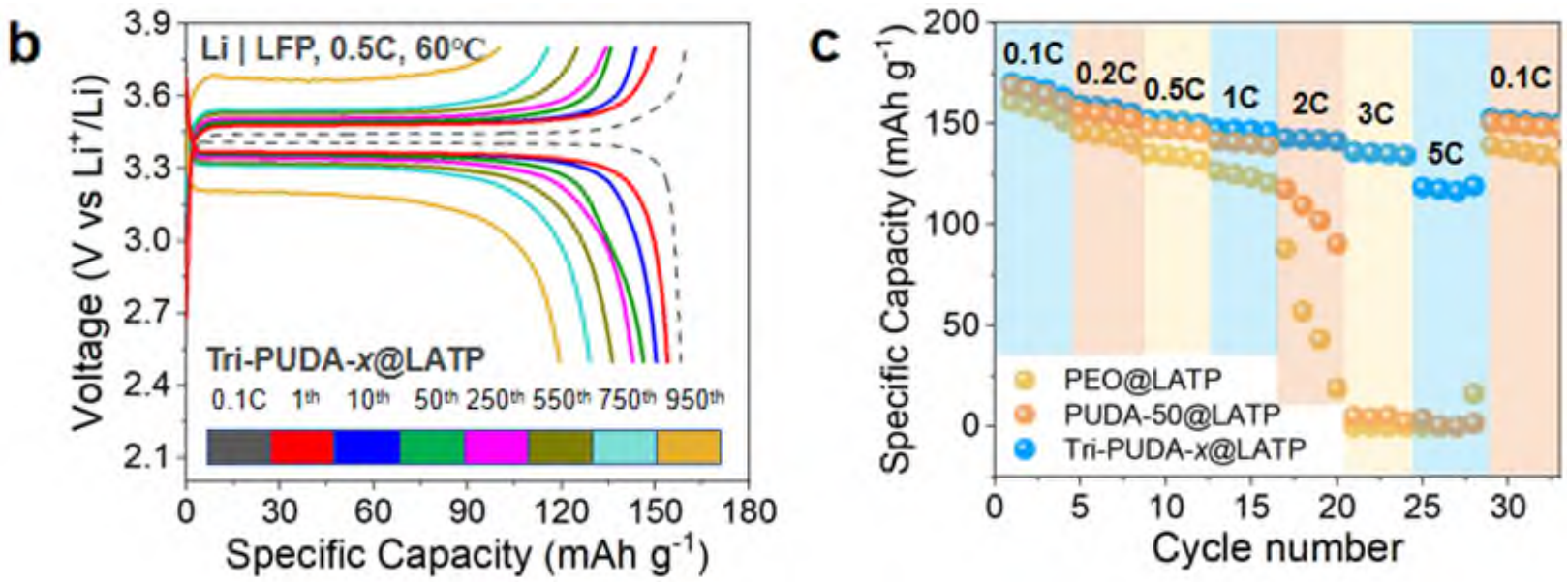

Fig. 5 | b, Galvanostatic charge–discharge profiles of solid-state Li|LFP cells with Tri-PUDA-*x*@LATP at 0.5C. **c**, Rate capability of solid-state Li|LFP cells with different electrolytes.

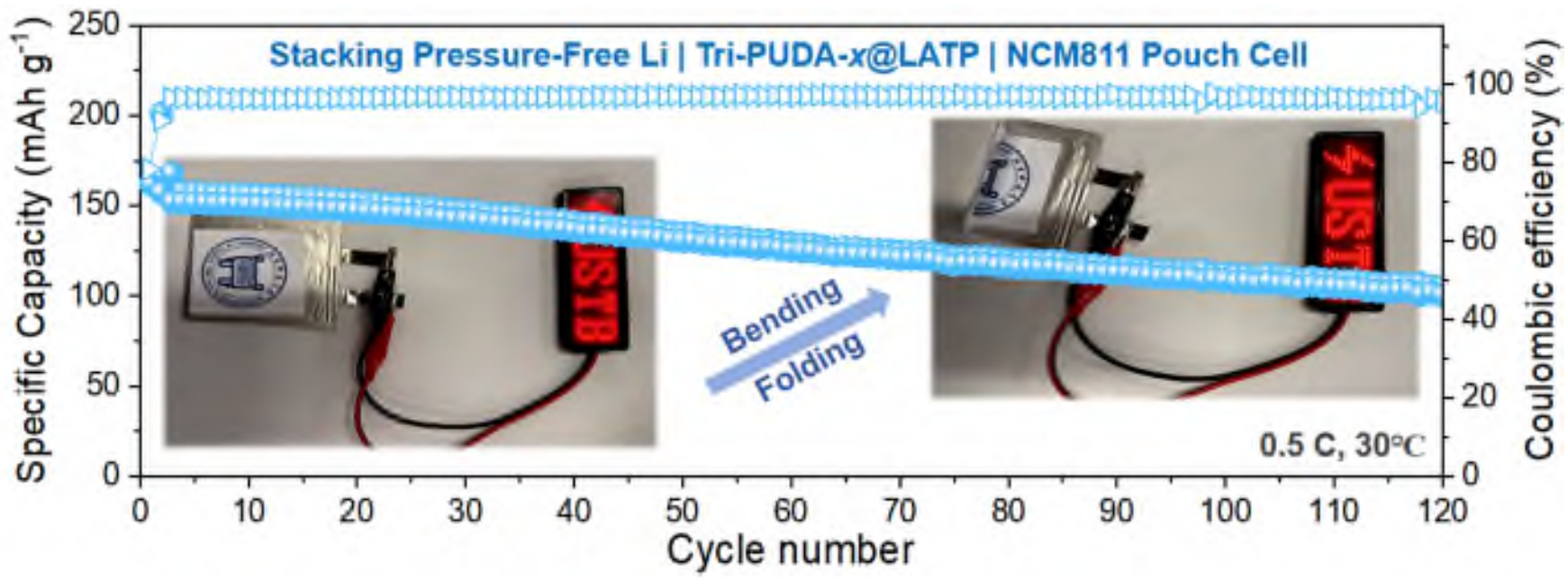

Fig. 5 | g, Cycling stability of solid-state Li|Tri-PUDA-*x*@LATP|NCM811 pouch cell without applied stacking pressure at 0.5C.

To address the Reviewer's concern more explicitly, we will add the following clarifications in the revised manuscript:

In Section 2.3 (**Page 18**):

While the PUDA-10 and PUDA-100 layers exhibit a lower bulk ionic conductivity compared to the percolated PUDA-50 layer, their primary function within the MIGE is to ensure optimal interfacial stability at the anode and cathode, respectively. The integrated Tri-PUDA-*x*@LATP electrolyte exhibits an effective ionic conductivity of 4.12×10^{-5} S cm^{-1} with a low activation energy of 0.24 eV. Crucially, the homologous polymer matrix creates a monolithic structure without internal ion-migration barriers, ensuring sufficient Li^+ flux across the cell. The Li^+ transference number (t_{Li^+}) of the Tri-PUDA-*x*@LATP electrolyte reached 0.605 at 25 °C (**Fig. S12**), significantly higher than that of conventional PEO-based systems (typically <0.3).

7. Fig. 4d presents a very interesting characterization and is greatly appreciated.

Author Reply: We sincerely thank the Reviewer for highlighting the value of Fig. 4d. It provides direct visual evidence of the critical role played by the dynamic hydrogen-bonding network in achieving the ultra-viscoelasticity and exceptional cohesive strength of the PUDA matrix.

8. In Fig. 5d, the reported value of 6.78% might not appear to correspond to the

y-axis scale.

Author Reply: We sincerely thank the Reviewer for the careful observation and constructive comment. The value of 6.78% originally indicated in Fig. 5d corresponds to the weight loss percentage derived from thermogravimetric analysis (TGA). We have carefully re-examined the TGA data and revised the corresponding figure. Additionally, the raw TGA plot (see **Figure 1**) is provided below for full transparency.

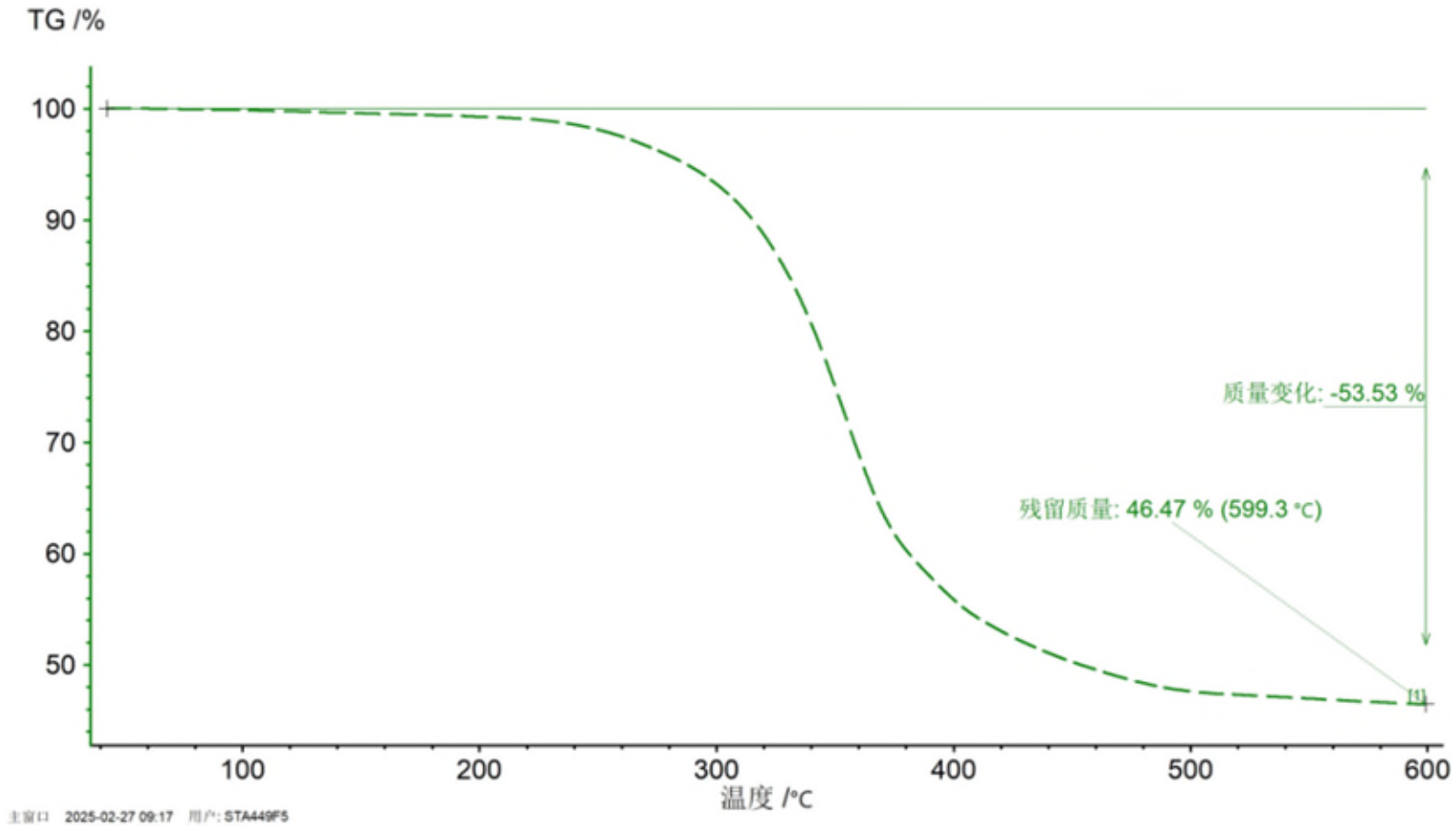

Figure 1. Raw TGA data plot.

Revised Figure in Main Manuscript (Page 26):

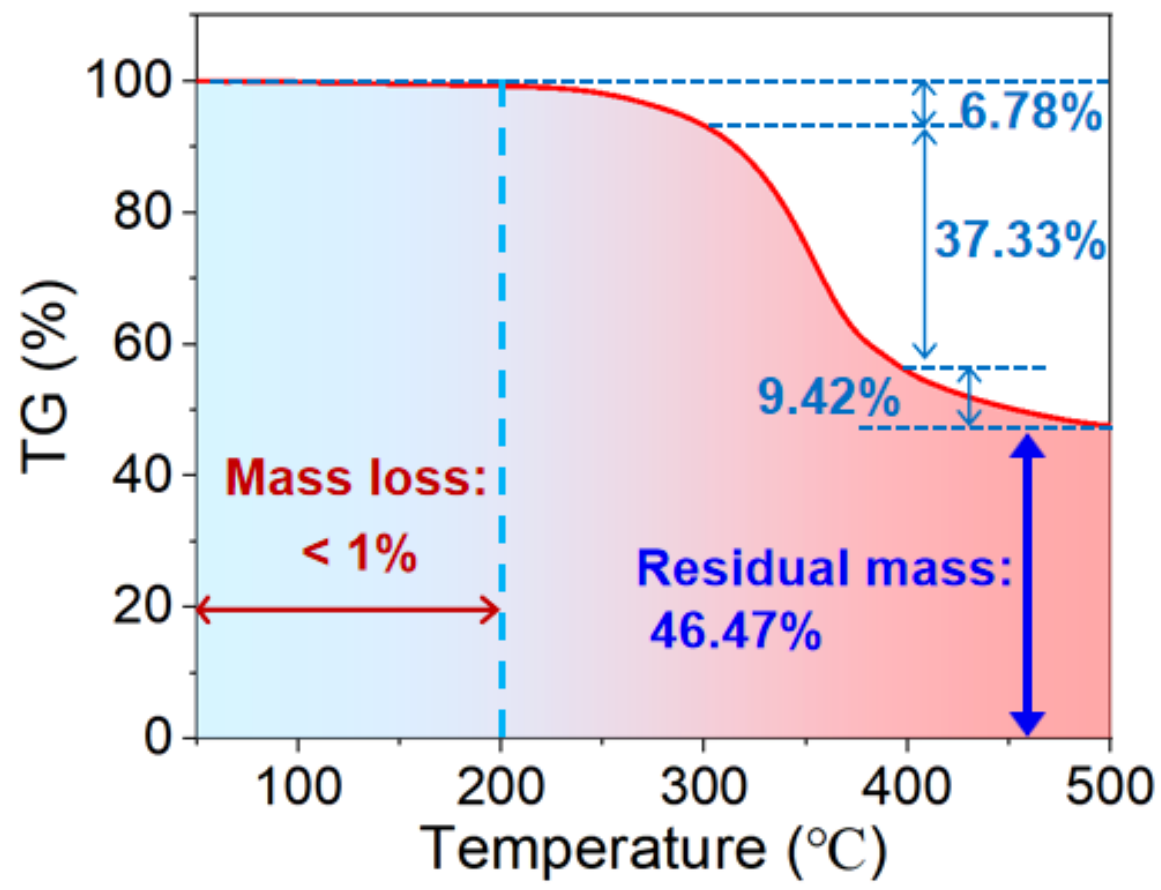

Fig. 5 | d, Thermogravimetric profiles (50-500°C, 10 K min^{-1} heating rate) of Tri-PUDA-*x*@LATP.

9. In Fig. 5e, the Coulombic efficiency exceeding 100% requires explanation.

Author Reply: We thank the Reviewer for raising this important point regarding the Coulombic efficiency (CE) values exceeding 100% observed during the long-term cycling of the Li/LFP cell with the Tri-PUDA-*x*@LATP electrolyte (Fig. 5e). We acknowledge that CE >100% is not typical in conventional liquid-electrolyte cells, and we appreciate the opportunity to clarify the underlying mechanism, which is closely related to the unique interfacial and self-healing properties of our MIGE system.

The observed CE>100% (reaching up to ~106.7%) arise from the gradual electrochemical reactivation of initially isolated or passivated lithium metal ("dead

Li") at the anode interface, coupled with continuous improvement of solid–solid contact during cycling. This is enabled by the ultra-viscoelastic and self-healing nature of the PUDA matrix. In solid-state batteries, especially those employing lithium-metal anodes, initial cycles often involve irreversible lithium loss due to poor interfacial contact, formation of isolated Li particles, or partial passivation. In our system, the dynamic hydrogen-bonding network of PUDA allows the electrolyte to maintain adaptive and conformal contact with the lithium anode. Over prolonged cycling, this viscoelastic interface can progressively recover some of the initially isolated lithium. This leads to a slight increase in the measured discharge capacity relative to the preceding charge capacity, thereby resulting in a CE temporarily above 100%.

This phenomenon has been documented in several high-quality studies on interface-adaptive solid electrolytes. For example (see **Figure 1**):

[Figure Redacted]

Figure 1. (a) *Nature Materials 2025, 24, 907–916.* (b) *Nature Communications 2023 14, 4077.*

Revised/Added Text in Main Manuscript:

To provide a clear explanation for readers, we have added the following sentences in Section 2.5 where Fig. 5e is discussed (**Page 24**):

The gradual capacity increase during the initial ~200 cycles at 30 °C reflects progressive interfacial optimization enabled by the viscoelastic PUDA matrix. Under near-ambient conditions, the ultra-elastic electrolyte dynamically improves solid-solid contact, which reduces interfacial resistance and gradually activates additional capacity from the LFP cathode. This self-adapting process stabilizes after ~200 cycles, leading to highly stable long-term cycling. The occasional exceedance of 100 % Coulombic efficiency is attributed to the reversible recovery of previously isolated lithium, facilitated by the continuous interfacial improvement and dynamic self-healing of the PUDA framework, a phenomenon consistent with prior reports on adaptive solid-state interfaces [32, 33].

References:

32. Wang, C. et al. The influence of pressure on lithium dealloying in solid-state and liquid electrolyte batteries. *Nat. Mater.* **24**, 907-916 (2025).
33. Lu, P. et al. Realizing long-cycling all-solid-state Li-In||TiS_2 batteries using $Li_{6+x}M_xAs_{1-x}S_5I$ (M=Si, Sn) sulfide solid electrolytes. *Nat. Commun.* **14**, 4077 (2023).

10. LATP is known to be unstable against lithium metal. The rationale for choosing LATP in this design should be clarified. Could other oxide-based solid electrolytes be applied within the same design framework?

Author Reply: We thank the Reviewer for this excellent question. The stability of LATP in direct contact with Li metal is indeed a well-known challenge, primarily due to the reduction of Ti^{4+}. Our design intentionally circumvents this issue through its spatially graded architecture. The key innovation lies in decoupling the mechanical/ionic function of the filler from the interfacial chemical stability requirement.

We selected LATP as a model oxide filler for several compelling reasons relevant to establishing our gradient electrolyte concept: LATP offers a compelling combination of high bulk ionic conductivity (~10^{-3} S cm^{-1}) and outstanding anodic stability (>5 V vs. Li^+/Li), making it an ideal material for the cathode-facing layer (PUDA-100) and the central ion-transport layer (PUDA-50), where high oxidation resistance and efficient Li^+ percolation are paramount. Its rigidity provides the essential mechanical scaffold within the composite to enhance the overall modulus and contribute to dendrite suppression. Its commercial availability and thoroughly characterized properties make it an excellent benchmark material to validate the fundamental feasibility of our Mechano-Integrated Gradient Electrolyte (MIGE) architecture, enabled by the novel PUDA matrix.

The key to stability lies in the functional design of the anode-facing layer (PUDA-10). This layer is deliberately formulated with a low LATP content (10 wt%), creating a composite that is dominated by the continuous, ultra-viscoelastic PUDA matrix. The primary role of the sparse LATP particles is to subtly modify the local mechanical environment and reduce the crystallinity of polymers, not to establish direct ionic contact with Li metal. The PUDA polymer phase acts as a conformal, protective barrier, ensuring intimate electrode contact while preventing direct physical contact between the majority of LATP particles and the Li metal anode. Li^+ transport in this region occurs predominantly through the PUDA matrix. The most direct experimental evidence for the success of this isolation strategy is the exceptional interfacial stability demonstrated in our electrochemical tests: the Li|Li symmetric cell with Tri-PUDA-*x*@LATP exhibits over 7500 hours of stable plating/stripping (Fig. 4a,b) without signs of rapid polarization growth or sudden failure. Persistent Ti^{4+} reduction would manifest as rapidly increasing polarization and early cell failure, which we do not observe.

We fully agree with the Reviewer that the MIGE framework is a versatile platform, not limited to LATP. The choice of filler can and should be tailored to the specific chemical requirements of each interface. The ultra-viscoelastic PUDA matrix, with its exceptional filler-loading capacity (>200 wt%) and strong adhesion, is inherently material-agnostic. This enables the integration of various functional fillers (e.g., garnet-type LLZO for enhanced anode compatibility, sulfides for higher conductivity, or core-shell engineered particles) into the same gradient architecture, allowing for spatial optimization of chemistry and function.

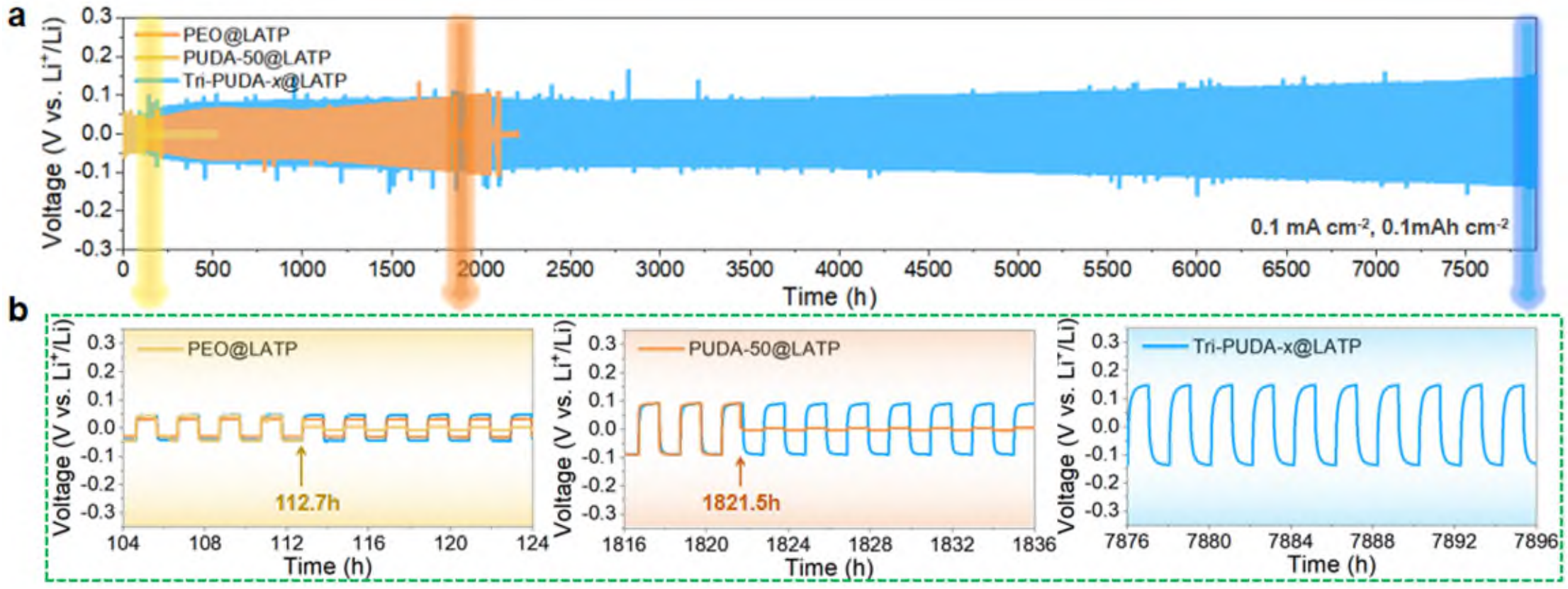

Fig. 4 | a,**b**, Long-term cycling performance of cells with PEO@LATP, PUDA-50@LATP, and Tri-PUDA-*x*@LATP at 0.1 mA cm^{-2}/0.1 mAh cm^{-2}, 60°C.

We have incorporated clarifications in the main text to directly address the Reviewer's concern and underscore the platform's versatility:

In Section 2.1 (Molecular Design), we have added the following sentence when introducing the anode layer (**Page 7**):

An anode-facing layer (PUDA-10, 10 wt% LATP) that is polymer-dominated, designed to achieve atomically conformal contact with Li metal while isolating the majority of the oxide filler from direct reduction at the anode interface.

In the Conclusion, we have expanded the discussion on the generality of the platform (**Page 31**):

Furthermore, the MIGE platform presents a generalizable design framework. While LATP serves as an effective model filler herein, the ultra-viscoelastic PUDA matrix can host a variety of inorganic fillers. Their composition can be spatially tailored within the gradient—for instance, employing anode-stable garnets (e.g., LLZO) at the Li interface while retaining high-conductivity, oxidation-resistant fillers like LATP at the cathode—to precisely address interfacial chemical requirements, offering a highly adaptable materials platform for diverse SSLMB chemistries.

Reviewer #3:

1. Can the PUDA fabricated in this study actually function as a lithium-ion conducting polymer electrolyte in a manner comparable to other polymer electrolytes? Even if lithium-ion solvation is possible through the ether groups, the manuscript also emphasizes that rigidity increases with the incorporation of biphenyl groups, raising concerns about whether conventional segmental motion–assisted ion transport can realistically occur. Please provide a more in-depth explanation of the ionic conductivity value of pristine PUDA shown in Figure 3d and its corresponding transport mechanism.

Author Reply: We thank the Reviewer for raising this critical point regarding the ion-transport mechanism in our polyurethane-based electrolyte (PUDA). We agree

that the incorporation of rigid biphenyl groups (from MDI and DBDA) enhances mechanical strength, which could theoretically impede chain mobility. However, our molecular design strategically utilizes microphase separation, a well-established principle in polyurethane elastomers, to create distinct domains: rigid hard segments for mechanical integrity and soft, amorphous poly(ethylene glycol) (PEG) segments for ion transport. Below, we provide a comprehensive, multi-scale explanation combining molecular design, theoretical simulation, experimental data, and literature precedent to clarify that PUDA not only functions as an effective Li^+-conducting polymer electrolyte but does so through a synergistic mechanism that surpasses conventional PEO-based systems

(1) Molecular design ensures amorphous character and segmental mobility

The PUDA matrix is synthesized from poly(ethylene glycol) (PEG, M_w=2000) soft segments, methylene diphenyl diisocyanate (MDI), and dual functional extenders (DBDA and ADH). While DBDA introduces rigid biphenyl units that form robust physical crosslinks within hard segments, the long, flexible PEG chains (soft segments) remain amorphous and highly mobile at room temperature. This is a classic design principle in polyurethane-based solid electrolytes, where microphase separation creates ionic conduction pathways primarily through the soft, PEG-rich domains (***Adv. Mater.* 2024, 36, 2409269. *Nat. Commun.* 2024, 15, 351. *Chem. Eng. J.* 2022, 430, 132659**). XRD patterns (Fig. 2d) confirm the suppressed crystallinity of PUDA-0, indicating a predominantly amorphous structure conducive to segmental motion.

(2) Dual ion-transport mechanism: Segmental motion assisted by targeted Li^+ hopping

Our data support a dual mechanism that overcomes the limitations of purely segmental motion: (i) Segmental Motion of PEG Chains: The ether oxygen (–O–) atoms in PEG are well-known Li^+ solvation sites. The flexible PEG chains provide the necessary segmental motion to facilitate Li^+ mobility, as in conventional PEO-based electrolytes. (ii) Dynamic Hopping via Functional Extenders: The key innovation lies in the strategic role of the ADH extender. Its hydrazide groups (–$CONHNH_2$) are stronger Lewis bases than ether oxygens. Density functional theory (DFT) calculations (Fig. 3c) confirm favorable binding energies for ADH–Li^+ (-0.38 eV) and DBDA–Li^+ (-0.55 eV). These extenders are integrated at the interfaces between soft and hard microdomains, creating a pervasive network of low-energy-barrier hopping sites. Consequently, Li^+ migration occurs via a combination of vehicular transport with mobile PEG chains and efficient hopping between adjacent coordination sites offered by ADH and DBDA. This synergistic mechanism is corroborated by molecular dynamics (MD) simulations (Fig. 3b), which show a significantly enhanced Li^+ self-diffusion coefficient for the PUD and PUA system compared to its precursors.

(3) Experimental conductivity and activation energy data

The room-temperature ionic conductivity of pristine PUDA (5.69×10^{-6} S cm^{-1}) and PUDA-50 (1.09×10^{-4} S cm^{-1}) (Fig. 3d) is comparable to or exceeds that of many reported polyurethane-based solid electrolytes at similar temperatures (***Electrochim. Acta* 2019, 299, 902–913. *Electrochim. Acta* 2017, 241, 526–534. *J. Appl. Polym. Sci.***

2017, 134 45554). More importantly, the activation energy (E_a) for ion conduction in pristine PUDA is 0.35 eV (Fig. 3e). This value is lower than that of pure PEO (0.40 eV) and falls within the range typical for segmental-motion-assisted polymer electrolytes (0.2-0.4 eV). The lower E_a for PUDA-0 directly indicates that the introduced extenders do not hinder but actually facilitate ion transport by providing additional, energetically favorable hopping pathways, thereby lowering the overall energy barrier for Li^+ migration.

(4) Comparison with literature on polyurethane-based electrolytes

Our results align with established work on polyurethanes for solid electrolytes. For instance, studies have shown that properly designed polyurethanes containing PEG soft segments can achieve ionic conductivities on the order of 10^{-7}-10^{-5} S cm^{-1} at room temperature, with transport occurring primarily through the amorphous PEG phase (***J. Power Sources 2018, 389, 84–92. Chem. Eng. J. 2022, 430, 132659. J. Electro. Soc. 2020, 167, 080525***). The rigid hard segments (often based on MDI and chain extenders) provide mechanical integrity without blocking ion conduction, as long as a continuous, mobile soft-segment phase exists. Our PUDA system represents a successful extreme optimization of this principle, achieving unprecedented viscoelasticity without sacrificing ionic transport functionality. Most importantly, the ultimate validation of PUDA as an effective Li^+ conductor is its exceptional performance in complete solid-state batteries, including long-term cycling stability in symmetric cells (>7900 h) and high-capacity retention in cells (74.4% after 1000 cycles).

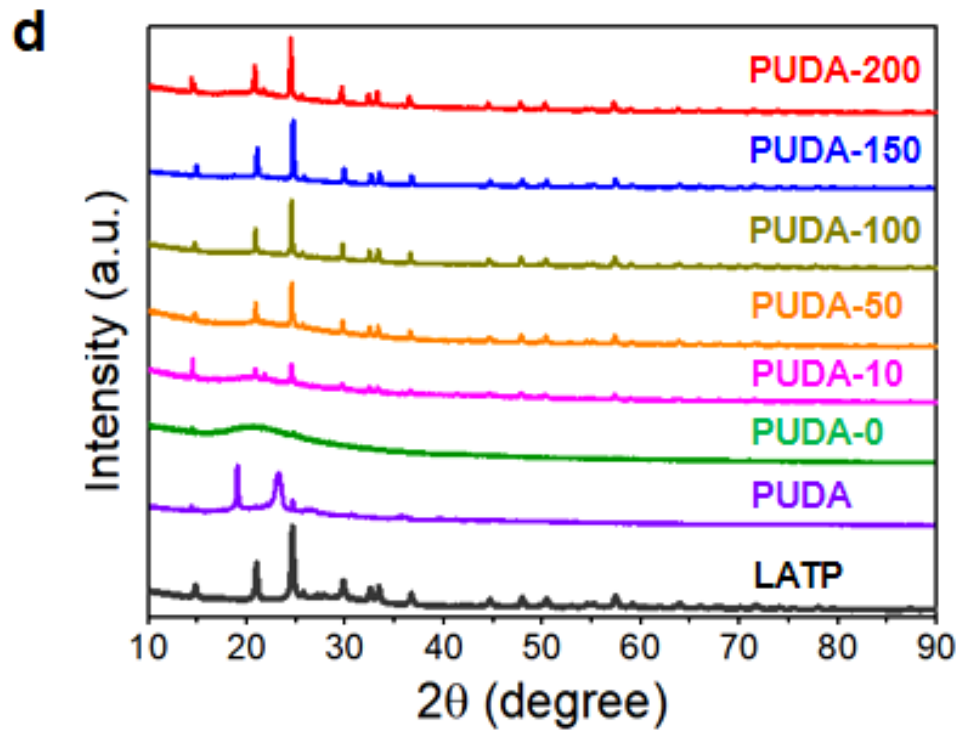

Fig. 2 | d, XRD patterns of LATP, PUDA, and PUDA-*x* composites.

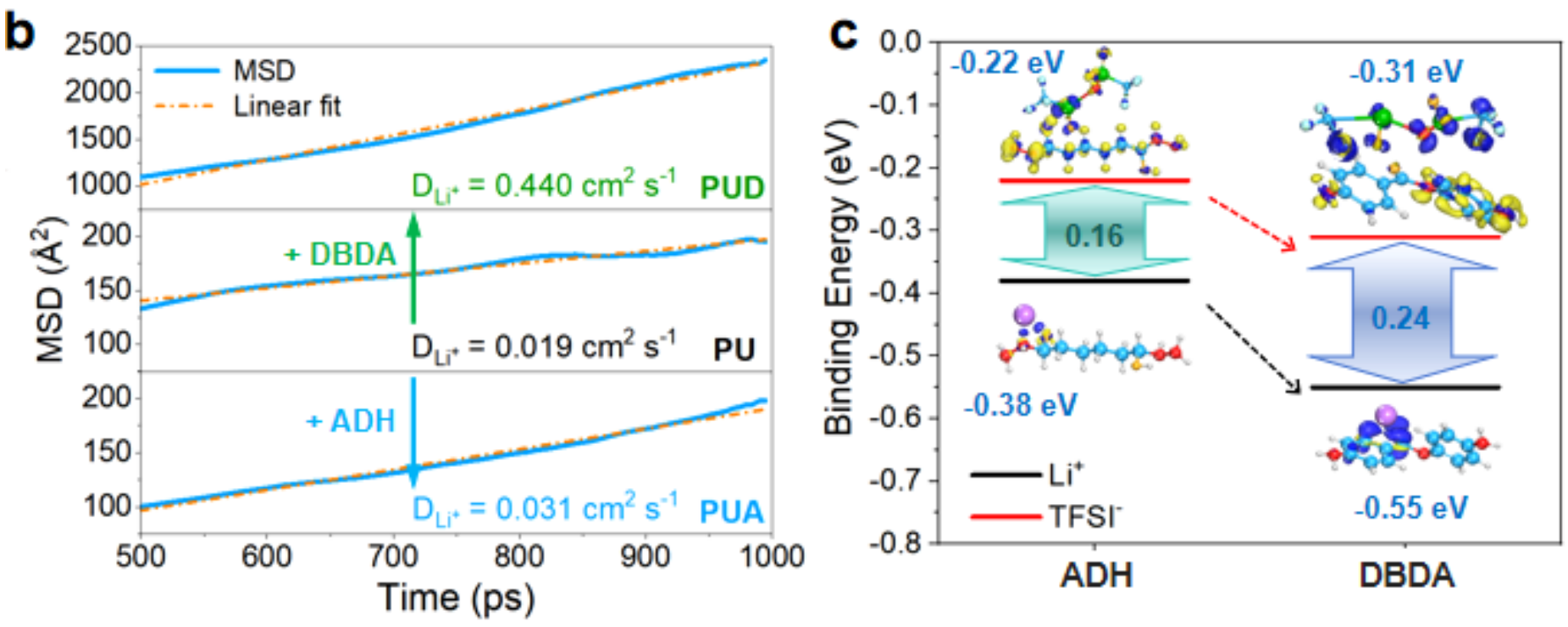

Fig. 3 | b, Mean squared displacement (MSD) from MD simulations, illustrating ion diffusion kinetics influenced by DBDA and ADH. **c**, Intermolecular interaction energies among DBDA, ADH, Li^+, and $TFSI^-$ species.

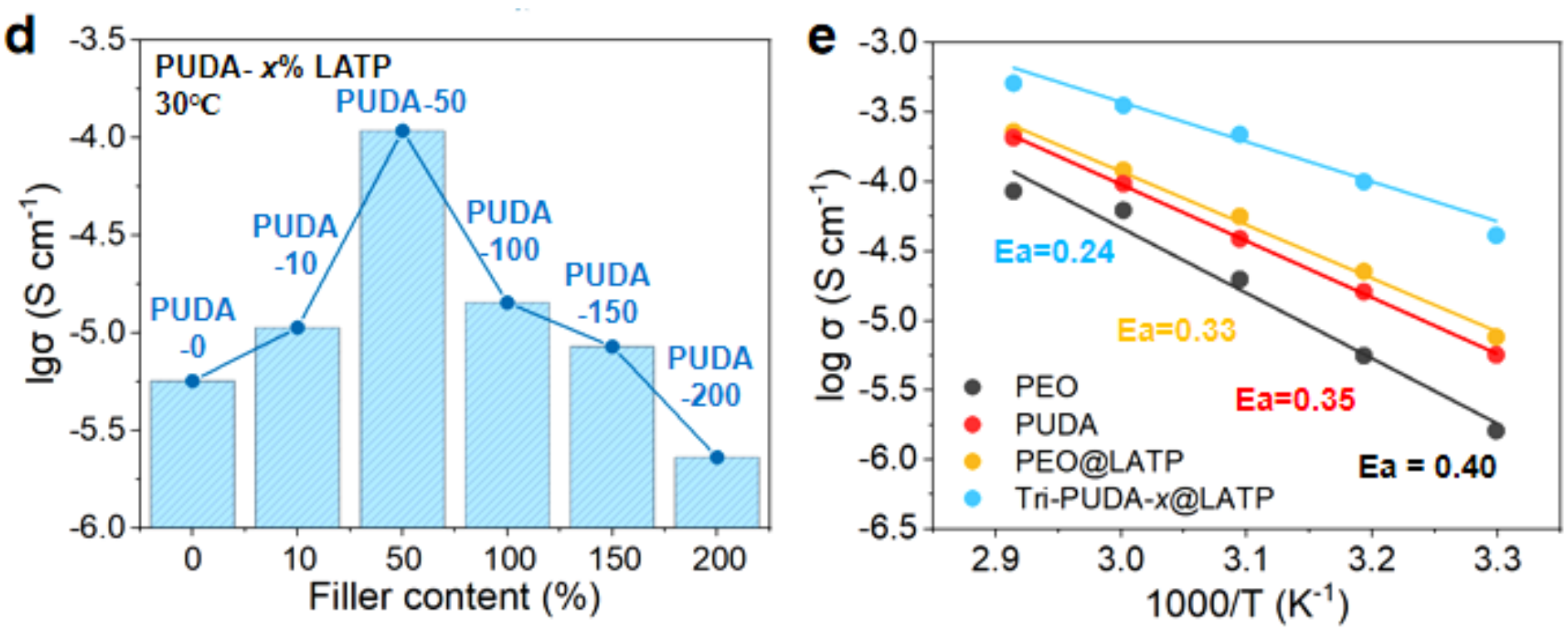

Fig. 3 | d, Ionic conductivity of PUDA-*x* electrolytes as a function of LATP content. **e**, Arrhenius plots of PEO, PUDA, PEO@LATP, and Tri-PUDA-*x*@LATP.

To address the Reviewer's concern more clearly, we will augment the discussion in Section 2.3 with the following text (**Page 17** and **Page 18**):

Binding energy calculations further elucidated the ion-coordination mechanism in PUDA. Values of -0.38 eV for ADH–Li^+, -0.55 eV for DBDA–Li^+, -0.22 eV for ADH–TFSI-, and -0.31 eV for DBDA–$TFSI^-$ suggest moderate yet selective ion coordination (**Fig. 3**c). The differential binding affinity (ΔE) between Li^+ and $TFSI^-$ facilitates (0.16 eV, ADH; 0.24 eV, DBDA) efficient salt dissociation, corroborating PUDA's superior ionic dissociation capability and supporting an ion-hopping conduction mechanism through alternating soft and rigid chain domains.

The ionic conductivity of pristine PUDA-0 (5.69×10^{-6} S cm^{-1} at 30 °C) and its lower activation energy (0.35 eV) compared to PEO (0.40 eV) confirm effective Li^+ transport (**Fig. 3**e). This performance originates from a dual transport mechanism enabled by the microphase-separated structure: (i) segmental motion within the continuous, amorphous PEG soft-segment phase solvates and mobilizes Li^+, while (ii) the engineered extenders (ADH and DBDA), located at the interfaces between soft and hard microdomains, provide a network of dynamic, low-energy-barrier hopping sites. This synergistic design ensures efficient Li^+ percolation while maintaining the mechanical robustness essential for dendrite suppression and interfacial stability.

2. How is the filler-loading capacity calculated? Is it appropriate to express this value as exceeding 200 in units of wt%?

Author Reply: We sincerely thank the Reviewer for this crucial comment, which highlights the need for clearer definitions. Below, we provide a detailed clarification and have incorporated corresponding revisions into the manuscript.

The filler loading (denoted as *x* in PUDA-*x*) is rigorously defined as the mass ratio of the inorganic filler (LATP) to the dry polymer matrix (PUDA), expressed in weight percent (wt%). For example, PUDA-200 indicates a LATP : PUDA mass ratio of 2:1 (i.e., 200 wt%). This convention is commonly employed in composite material science to describe systems where the filler content can substantially exceed the polymer mass, especially when highlighting exceptional polymer binding capacity.

The notation “>200 wt%” is used to emphasize the exceptional loading capability

of our PUDA matrix, which is a core innovation of this study. In conventional composite solid electrolytes such as PEO, PVDF, or PAN, filler loadings beyond 50–60 wt% typically lead to severe brittleness, film disintegration, and a drastic drop in elongation (<300%), resulting in high interfacial resistance and poor cycling (*Energy Storage Mater. 2020, 26, 448–456. Chem. Eng. J. 2022, 440, 135816. ACS Appl. Mater. Interfaces 2024, 16, 27280-27290.*). In contrast, our PUDA-200 composite (200 wt% LATP) maintains a cohesive, free-standing film with a remarkable elongation at break of 1477% (Fig. 2g). This exceptional mechanical tolerance enables the construction of continuous, triphasic percolation networks even at ultra-high inorganic contents. Thus, the "wt%" expression is not only mathematically appropriate but also functionally meaningful, as it directly correlates with the material's processing window and electrochemical performance.

Proposed Text Additions to Manuscript:

In Section 2.1 (Molecular Design), after introducing PUDA-*x* notation (**Page 7**):
The filler loading (*x* in PUDA-*x*) is defined as the weight percentage of LATP relative to the PUDA polymer matrix (e.g., 200 wt% denotes a LATP : PUDA mass ratio of 2:1).

3. The description that LATP is "spatially graded" in the membrane is somewhat confusing. Moreover, this concept is introduced before Figure 2, whereas the supporting data appear in Figure 3, which may further confuse readers. A reorganization of the manuscript structure seems necessary.
Author Reply: We sincerely thank the Reviewer for this insightful and critical comment. We agree that the original presentation of the "spatially graded" architecture could be clearer, particularly regarding the sequence of concept introduction versus experimental validation. To address this, we have thoroughly reorganized the manuscript to ensure a logical, step-by-step presentation: the gradient design is now explicitly introduced together with its schematic, immediately followed by the polymer matrix characterization that enables it, and then directly validated by microstructural, compositional, and functional data. Below we detail the specific modifications made in the text.

Revised/Added Text in Main Manuscript:

(1) We have revised the description of the MIGE design to be more precise and self-contained. The updated text in Section 2.1 now reads (**Page 7**):

Building on this molecularly tailored matrix, we constructed a Mechano-Integrated Gradient Electrolyte (MIGE, Tri-PUDA-*x*@LATP) in which the LATP filler content is programmed to vary continuously across the membrane thickness, as schematically contrasted in **Fig. 1**c.

The architecture comprises three functionally distinct layers: an anode-facing layer (PUDA-10, 10 wt% LATP) that is polymer-dominated, designed to achieve

atomically conformal contact with Li metal while isolating the majority of the oxide filler from direct reduction at the anode interface; an ion-transport interlayer (PUDA-50, 50 wt% LATP) infused into a porous PE scaffold, which forms a continuous 3D percolating network to ensure high ionic conductivity and a cathode-facing layer (PUDA-100, 100 wt% LATP) engineered as a ceramic-dense barrier, where LATP particles forms a continuous protective layer with the PUDA binder serving as a conformal adhesive at particle boundaries.

(2) We have enriched Fig. 1c to provide a clearer visual representation of the three-layer gradient architecture, explicitly labeling the LATP content in each functional layer (**Page 9**).

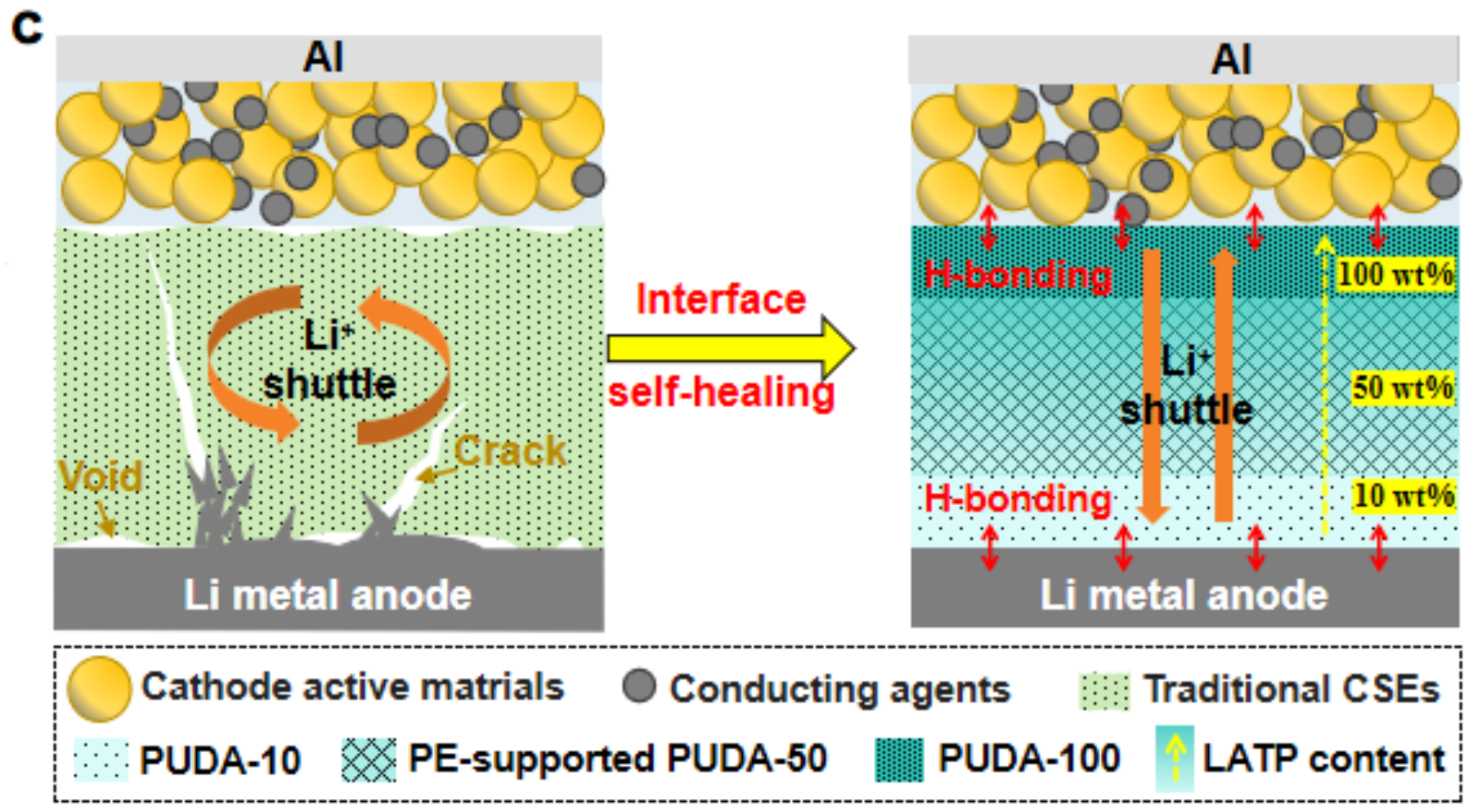

Fig. 1 | c, Structural comparison between conventional laminated and integrated MIGE-based SSLMB configurations.

(3) To avoid confusion between concept introduction and evidence presentation, we have revised the opening sentences of Sections 2.2-2.5 to explicitly signal what each section will validate:

Section 2.2 now begins (**Page 11**): The composition-dependent structural evolution and mechanical properties of the Tri-PUDA-*x*@LATP electrolyte were systematically characterized to validate its gradient architecture and multifunctional performance.

Section 2.3 opens with (**Page 17**): Building on the confirmed structural gradation, we next evaluate how this spatially designed architecture translates into graded electrochemical and mechanical functionality.

(4) Throughout the results discussion (Sections 2.3 to 2.5), we have inserted concise phrases that explicitly tie each key performance observation back to the gradient architecture:

Regarding the high oxidation stability (Sections 2.3, **Page 19**): Linear sweep voltammetry (**Fig. 3**f) reveals that the Tri-PUDA-*x*@LATP electrolyte achieves an expanded electrochemical stability window up to 4.9 V. This value significantly exceeds those of PEO-based polymer electrolytes (3.8 V) and PEO-LATP composites (4.2 V). The enhanced oxidative stability originates from the synergistic combination of a more robust polyurethane backbone[12] and, dominantly, the formation of a dense,

ceramic-rich protection layer at the cathode interface.

Explaining the long-term Li metal cycling (Sections 2.4, **Page 21**): This outstanding cyclability stems from the gradient rigidity-flexibility coupling inherent to the MIGE architecture.

Discussing rate performance (Sections 2.5, **Page 23**): By contrast, cells employing PEO-LATP or PUDA-50@LATP exhibited rapid capacity decay above 2C, highlighting the essential role of continuous triphasic percolation established by the graded filler distribution in sustaining high-rate performance.

(5) To eliminate any ambiguity about how the gradient is realized, we have added a detailed description of the roll-to-roll slot-die coating process in the Supporting Information:

The Tri-PUDA-*x*@LATP MIGE was fabricated via a sequential slot-die coating process. A porous polyethylene (PE) substrate (70% porosity, 12 μm thickness) was first coated with a PUDA-50 slurry, dried, and lightly calendared. Subsequently, the PUDA-10 layer was coated atop, followed by simultaneous double-side coating of the PUDA-50 and PUDA-100 layers on the opposite side of the PE substrate. Each step involved low-temperature drying (60-80°C). This process results in a continuous, monolithic membrane with a programmed, three-layer gradient in LATP content, as confirmed by cross-sectional analysis.

4. In this electrolyte membrane, how does lithium-ion transport occur in regions containing only 10 wt% LATP? Even if specific layers exhibit high LATP content and thus good ionic conductivity, layers with low LATP content are likely to determine the membrane's overall ionic conductivity. Therefore, additional clarification and discussion on this point are required.

Author Reply: We sincerely thank the Reviewer for raising this important point regarding the ionic conductivity of the functional layers within our MIGE architecture. We fully agree that sufficient ion transport across the entire electrolyte is critical for cell performance. Our design philosophy and the resulting electrochemical data demonstrate that the slightly lower conductivity in the electrode-adjacent layers is a deliberate and optimal trade-off, and does not limit overall cell performance for the following reasons:

(1) The Reviewer correctly infers that the bulk ionic conductivity of the isolated PUDA-10 layer (~1.01×10^{-5} S cm^{-1} at 30 °C) is lower than that of the central percolated PUDA-50 layer (~1.09×10^{-4} S cm^{-1}). However, the effective ionic conductivity of the integrated Tri-PUDA-*x*@LATP (MIGE) electrolyte is 4.12×10^{-5} S cm^{-1} at 30°C (Fig. 3d), with a remarkably low activation energy of 0.24 eV (Fig. 3e). This value, only ~2.6 times lower than that of the best-conducting mid-layer, governs the total cell impedance and is highly competitive among dry-processed polymer-ceramic composites. The low activation energy signifies favorable transport kinetics throughout the integrated structure.

(2) The MIGE architecture is fundamentally designed to decouple and prioritize functions at each interface, which we identify as the more critical failure mode in

long-cycling SSLMBs than maximizing bulk conductivity in every region. The primary function of anode side (PUDA-10) is to provide atomically conformal, dynamically adaptive contact with the Li metal (~4853% strain), accommodating volume changes and suppressing void formation. The moderate conductivity here is a calculated trade-off for achieving this essential interfacial compliance. The ion transport in this layer occurs predominantly through the highly conductive PUDA polymer matrix, which is sufficient to support the localized current flux at the interface.

(3) Crucially, as we highlighted, all three layers share an identical, homologous PUDA polymer matrix. During the final hot-pressing stage of our manufacturing process, these layers fuse into a monolithic, single-phase structure. This eliminates the sharp internal interfaces and associated ion-migration barriers that plague heterogeneous multilayer systems. Consequently, Li^+ traverse the electrolyte thickness without encountering sudden energy jumps, making the effective conductivity of the integrated whole the relevant metric, not the conductivity of individual layers in isolation.

The outstanding cell performance presented in the manuscript provides direct evidence that ion transport is not a limiting factor. The Li/LFP maintains excellent capacity retention up to 5C (118.5 mAh g^{-1}) and recovers fully upon returning to 0.1C (Fig. 5c). This would not be possible if the electrode-adjacent layers were severe ionic transport bottlenecks. The Li/LFP cell shows a very low polarization voltage (~0.117 V) at 0.5C, with a minimal fade rate of 0.026% per cycle (Fig. 5a, b) over 1000 cycles. Significant ionic blockage at the interfaces would manifest as a rapidly increasing polarization, which we do not observe. The operation of Li|NCM811 cell (Fig. 5g) further confirms that the ion supply through the cathode-side layer is sufficient for stable cycling.

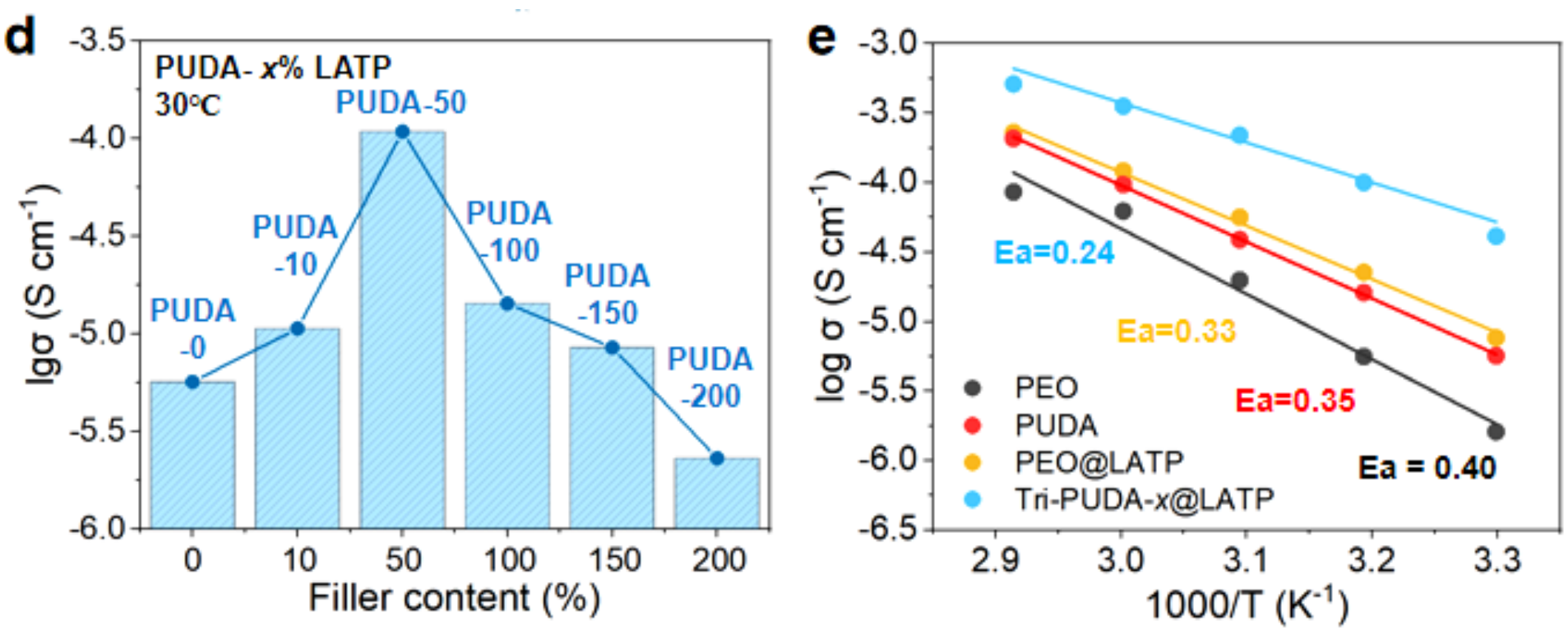

Fig. 3 | d, Ionic conductivity of PUDA-*x* electrolytes as a function of LATP content. **e**, Arrhenius plots of PEO, PUDA, PEO@LATP, and Tri-PUDA-*x*@LATP.

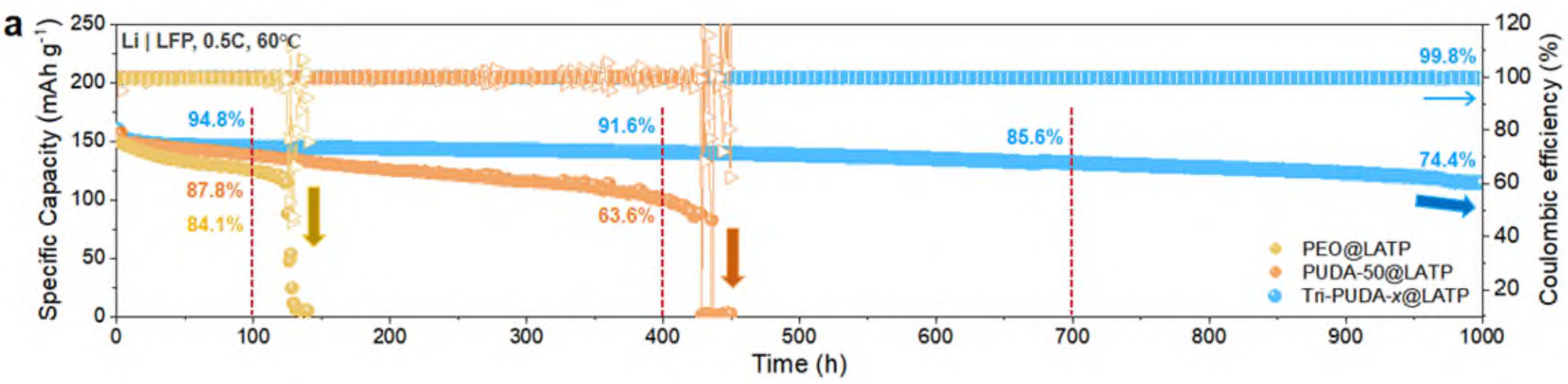

Fig. 5 | a, Long-term cycling performance of solid-state Li|LFP cells with PEO@LATP, PUDA-50@LATP, and Tri-PUDA-*x*@LATP at 0.5C.

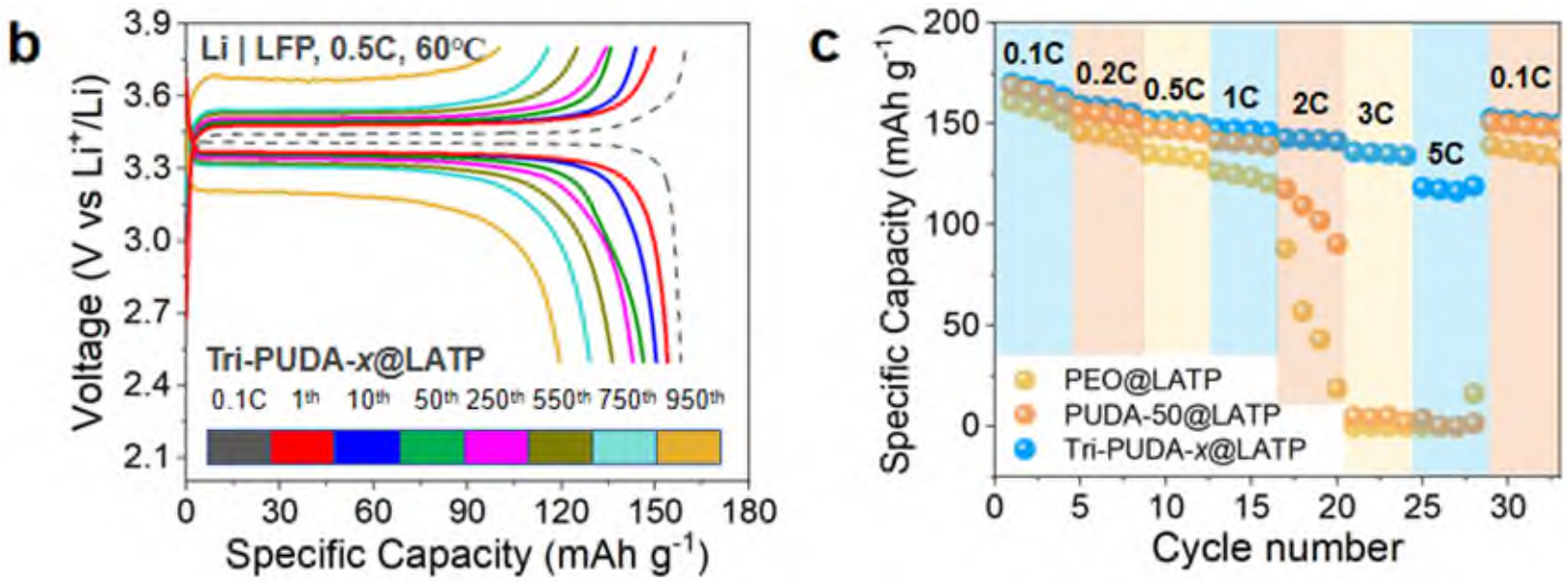

Fig. 5 | b, Galvanostatic charge–discharge profiles of solid-state Li|LFP cells with Tri-PUDA-*x*@LATP at 0.5C. **c**, Rate capability of solid-state Li|LFP cells with different electrolytes.

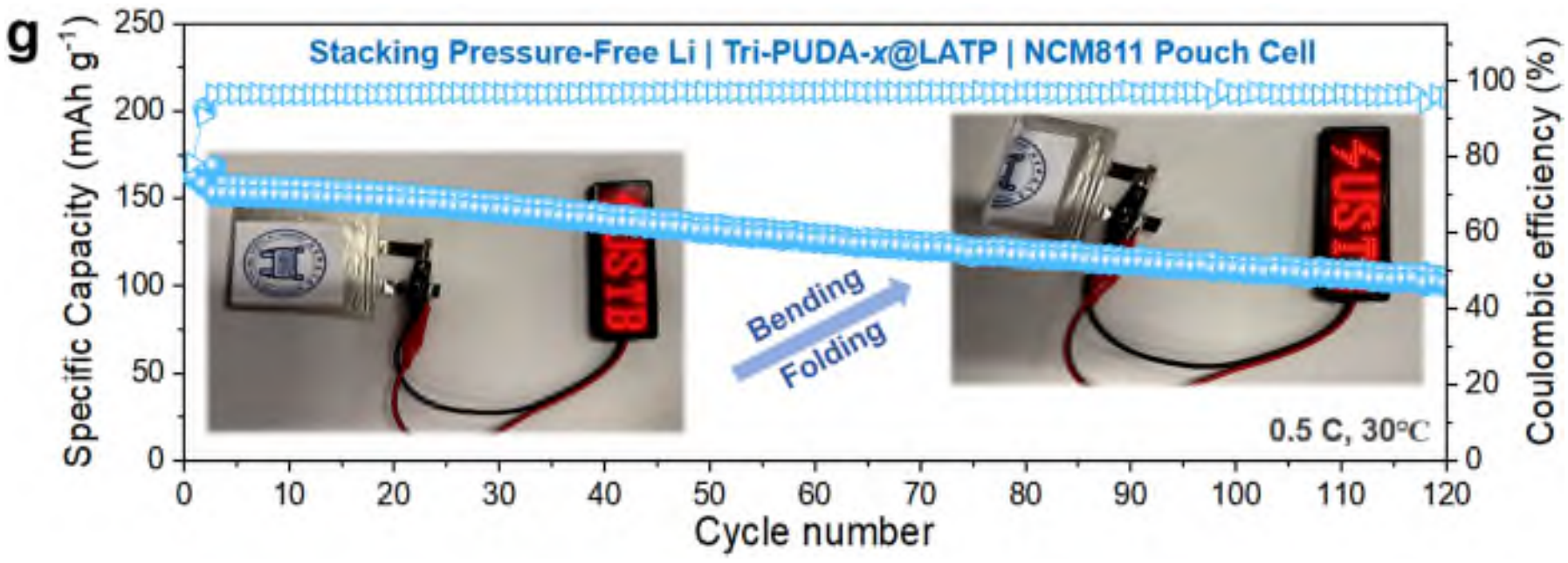

Fig. 5 | g, Cycling stability of solid-state Li|Tri-PUDA-*x*@LATP|NCM811 pouch cell without applied stacking pressure at 0.5C.

To address the Reviewer's concern more explicitly, we will add the following clarifications in the revised manuscript:

In Section 2.3 (**Page 18**):

While the PUDA-10 and PUDA-100 layers exhibit a lower bulk ionic conductivity compared to the percolated PUDA-50 layer, their primary function within the MIGE is to ensure optimal interfacial stability at the anode and cathode, respectively. The integrated Tri-PUDA-*x*@LATP electrolyte exhibits an effective ionic conductivity of 4.12×10^{-5} S cm^{-1} with a low activation energy of 0.24 eV. Crucially, the homologous polymer matrix creates a monolithic structure without internal ion-migration barriers, ensuring sufficient Li^+ flux across the cell. The Li^+ transference number (t_{Li^+}) of the Tri-PUDA-*x*@LATP electrolyte reached 0.605 at 25 °C (**Fig. S12**), significantly higher than that of conventional PEO-based systems (typically <0.3).

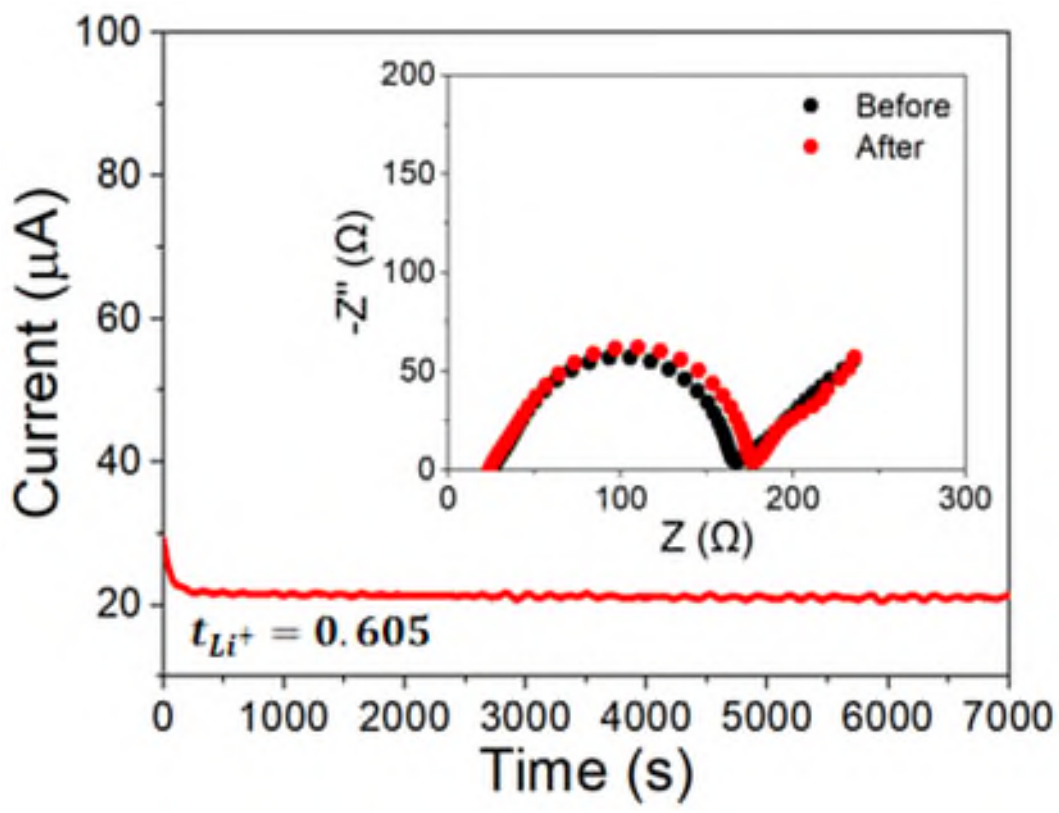

Fig. S12 | Chronoamperometry curve and AC impedance spectra before and after polarization of the Li|Tri-PUDA-*x*@LATP|Li symmetric cell.

5. In Figure 1d, does "Sp" correspond to the wt% mentioned earlier? If so, the terminology should be unified for consistency.

Author Reply: We sincerely thank the Reviewer for raising this important point regarding terminology consistency. The Reviewer is correct to note that the parameter "S_p" used in our phase-field simulations is conceptually related to, but distinct from, the weight percentage (wt%) of LATP filler discussed in the experimental sections. To avoid confusion and improve clarity, we have revised the manuscript and the Supporting Information to explicitly define these terms and their relationship.

In our multiphysics phase-field model, which simulates Li^+ transport efficiency from left to right in a composite electrolyte, we utilize three key dimensionless parameters:

C_p represents the normalized Li^+ concentration distribution (%). A higher C_p value indicates better overall ion diffusion performance. M_p represents the area fraction (%) occupied by the ion-conducting inorganic filler particles and the organic-inorganic interphases. S_p is defined as the area fraction of the filler and interphase relative to the polymer matrix area (%), whereas wt% is proportional to the experimental filler-to-polymer mass ratio. They are not numerically identical because the simulation uses normalized, dimensionless inputs for ion concentration and conductivity.

To eliminate any potential confusion, we have made the following revisions to ensure consistency:

In Section 2.1, we have clarified the relationship immediately following the description of the phase-field simulations (**Page 10**):

In the phase-field simulations, the filler content is represented by the dimensionless parameter S_p, defined as the area fraction of the inorganic filler and interphase relative to the polymer matrix, S_p is qualitatively proportional to, but distinct from, the experimental LATP weight percentage (wt%).

In the caption of Fig. 1d, the description has been updated to (**Page 9**):

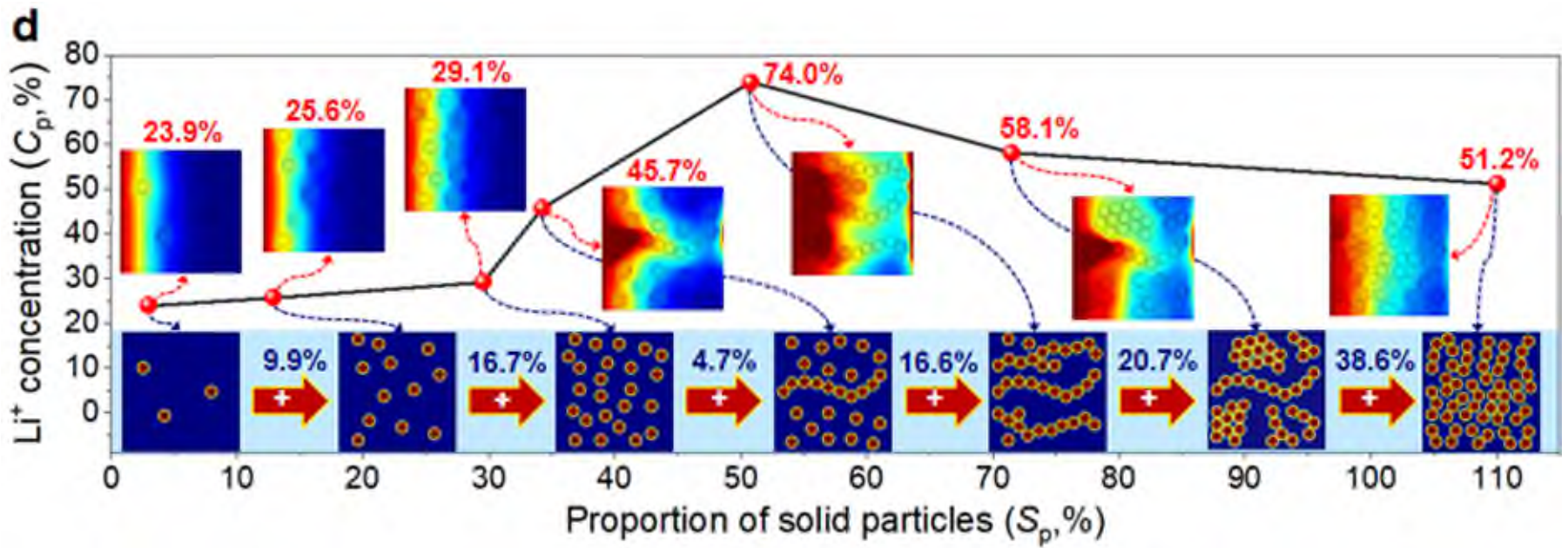

Fig. 1 | d, COMSOL-simulated Li^+ flux profiles as a function of inorganic filler content, expressed via the area fraction of solid particles relative to the polymer matrix (dimensionless parameter S_p).

In the Supporting Information, we have added a concise subsection explicitly defining C_p, M_p, and S_p, and stating their conceptual link to the experimental wt% metric.

The normalized Li^+ concentration distribution, C_p:

$$C_p = \frac{\iint c d\Omega}{\Omega} \times 100\% \tag{6}$$

The area fraction occupied by filler particles and organic-inorganic interphases, M_p:

$$M_p = \frac{\iint m d\Omega}{\Omega} \times 100\% \tag{7}$$

The filler-to-polymer area ratio, S_p:

$$S_p = \frac{M_p}{1 - M_p} \times 100\% \tag{8}$$

where c is the dimensionless Li^+ concentration field, m is the dimensionless field representing the filler and interphase domains, and Ω is the total area of the computational domain. The parameter S_p is qualitatively correlated with the experimental mass loading (wt%) of LATP filler, as both reflect the relative abundance of the inorganic phase within the composite.

6. Figure 2f suggests that high bulk and shear moduli are beneficial. However, the manuscript does not clearly explain why higher mechanical moduli are advantageous in this context. Further justification is needed.

Author Reply: We thank the Reviewer for this insightful comment. We agree that a more precise explanation of the role of mechanical moduli is needed. The improved bulk (~1.56 GPa) and shear (~0.89 GPa) moduli of the PUDA matrix, compared to pristine PEG or conventional PEO, are advantageous not because they represent the highest achievable values, but because they reflect an optimized balance between rigidity and viscoelasticity that enables multiple critical functions unattainable in prior composite electrolytes.

A sufficiently high shear modulus is a well-established descriptor for a material's

ability to resist plastic deformation and physically block the penetration of lithium dendrites. The enhanced modulus of PUDA, provided by the rigid segments (MDI and DBDA) and the dense hydrogen-bonding network, contributes to the dendrite-blocking capability of the composite layers (especially PUDA-50 and PUDA-100), while avoiding the catastrophic brittleness of pure ceramics due to the dynamic, energy-dissipative bonds introduced by ADH.

The balanced modulus profile works synergistically with PUDA's extreme viscoelasticity (>5000% fracture strain). The bulk modulus ensures dimensional stability and prevents excessive creep under processing or stack pressure, while the viscoelastic nature maintains atomic-scale conformal contact with the lithium metal anode during repeated plating/stripping, thereby preventing interfacial delamination. This dual characteristic prevents the interfacial delamination commonly observed in purely soft polymers with low moduli.

For solution-based, high-speed roll-to-roll (R2R) manufacturing, the electrolyte must exhibit strong cohesion and tensile strength (>80 MPa) to withstand coating, drying, and winding stresses. PUDA's optimized modulus, together with its inherent tackiness, allows it to: thoroughly infiltrate and tenaciously bind to the porous polyethylene scaffold, and uniformly coat and bridge adjacent LATP particles even at loadings >200 wt%, forming a continuous three-phase (polymer-filler-interphase) percolation network without cracking, a feat unachievable with low-modulus binders such as PEO.

In summary, the significance of the enhanced moduli lies in enabling PUDA to serve as a multifunctional structural platform that concurrently: (i) resists dendrite penetration, (ii) maintains cycling-stable lithium interfaces, and (iii) provides the cohesive strength required to integrate a porous scaffold and ultra-high filler loading into a single, robust, and scalable membrane. This integrated mechanical design is a cornerstone of the MIGE architecture and its practical viability.

Revised Text to be Added in Manuscript (Section 2.2, Page 14):

The optimized mechanical profile of PUDA, characterized by balanced bulk (1.56 GPa) and shear (0.89 GPa) moduli coupled with extreme viscoelasticity, is pivotal for its multifunctional role. The enhanced modulus, imparted by the rigid segments (MDI and DABA) and the supramolecular hydrogen-bonding network, contributes to the composite's ability to resist lithium dendrite penetration. Crucially, the modulus ensures dimensional stability and prevents excessive creep, while the viscoelasticity guarantees conformal contact with lithium metal during cycling. Furthermore, this unique combination of cohesion and deformability underpins PUDA's exceptional efficacy as a binder, enabling robust adhesion to the porous PE scaffold and homogeneous integration of LATP particles. This facilitates the construction of continuous ion-transport pathways within a freestanding, flexible film, addressing a core limitation in conventional composite electrolytes where high filler content typically compromises mechanical integrity and processability.

7. What is the origin of the difference in crystallinity between PUDA and PUDA-0?

Author Reply: We sincerely thank the Reviewer for raising this important point regarding the distinct crystallinity observed between PUDA and PUDA-0. The distinct crystallinity originates from the salt-induced amorphization of the semi-crystalline polyurethane matrix (PUDA) upon the incorporation of LiTFSI and ionic liquid to form the solid electrolyte (PUDA-0). This is a well-established phenomenon in polymer electrolytes where dissociated ions disrupt chain packing, suppress crystallinity, and enhance segmental mobility, key for achieving high ionic conductivity.

As clearly shown in Fig. 2d, the sharp crystalline diffraction peaks of pure PUDA (associated with ordered hard-segment domains formed by DABA and MDI) are replaced by a broad amorphous halo in PUDA-0. This structural transition is driven by multiple, synergistic mechanisms enabled by the introduced lithium salt and ionic liquid:

Our results show strong coordination between Li^+, $TFSI^-$, and the ether oxygen of PEG, as well as the amide groups of ADH and DBDA, with binding energies of -0.38 eV (ADH–Li^+), -0.55 eV (DBDA–Li^+), -0.22 eV (ADH–$TFSI^-$) and -0.31 eV (DBDA–$TFSI^-$) (Fig. 3b,c). This coordination competes with and weakens the inter-chain dipole-dipole interactions necessary for PEG crystallization, effectively pinning the chains and reducing their ability to form ordered lattices. The bulky $TFSI^-$ anions and the cations/anions of the ionic liquid act as molecular spacers. Their incorporation increases the inter-chain distance and free volume, physically disrupting the close packing of both soft (PEG) and hard (urethane/urea) segments. The carbonyl (C=O) and sulfonyl (S=O) groups of $TFSI^-$ and the ionic liquid can engage in competitive hydrogen bonding with the -NH donors of the urethane/urea linkages. This interferes with the native hydrogen-bonding network that stabilizes the hard-segment microdomains, further promoting amorphization..

The combined effect is a deliberate transition from a semi-crystalline polymer to a predominantly amorphous polymer-salt complex. This amorphization is functionally essential: it enhances segmental chain motion, which is the primary driver for Li^+ transport in polymer electrolytes, thereby directly contributing to the enhanced room-temperature ionic conductivity observed in PUDA-0.

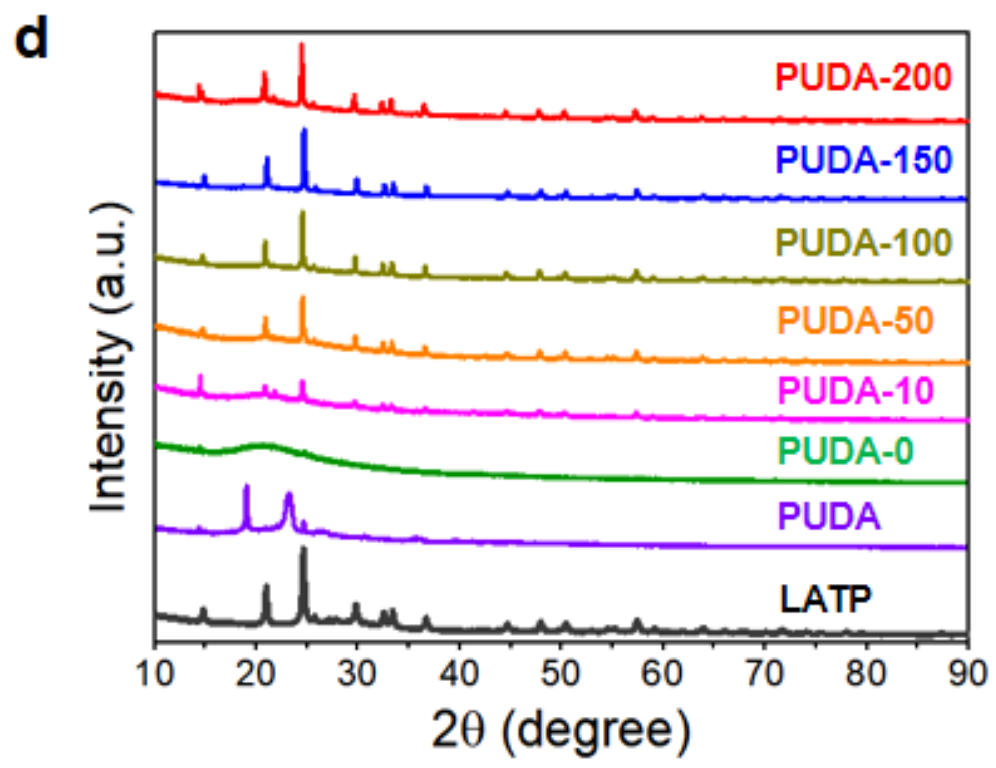

Fig. 2 | d, XRD patterns of LATP, PUDA, and PUDA-*x* composites.

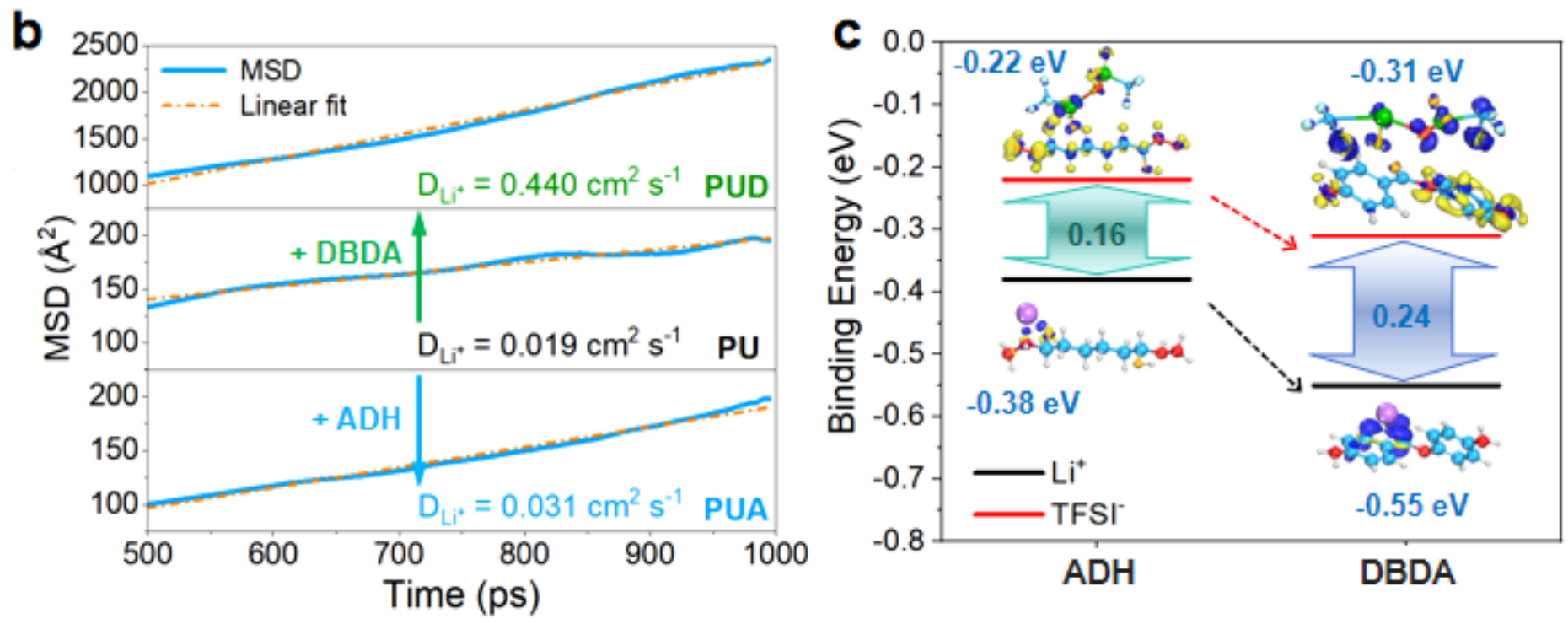

Fig. 3 | b, Mean squared displacement (MSD) from MD simulations, illustrating ion diffusion kinetics influenced by DBDA and ADH. **c**, Intermolecular interaction energies among DBDA, ADH, Li^+, and $TFSI^-$ species.

Revised/Added Text in Main Manuscript:

To explicitly address this point, we have added the following clarification in the revised manuscript (Section 2.2, **Page 13**):

The transition from sharp diffraction peaks in pure PUDA to a broad amorphous halo in PUDA-0 signifies a salt-induced amorphization of the polymer matrix. This common, yet crucial, effect in polymer electrolytes arises from the strong coordination of Li^+ to polar groups (e.g., -C-O-C- in PEG, -NH-CO- in ADH/DABA) and the steric presence of $TFSI^-$, which collectively disrupt polymer chain packing. The resulting amorphous structure enhances segmental mobility and facilitates Li^+ transport.

8. In some parts of the manuscript, the thickness of the fabricated electrolyte membrane is described as 12 μm, whereas in other sections it appears to be 40 μm. This inconsistency should be corrected.

Author Reply: We sincerely thank the Reviewer for this careful observation. The apparent discrepancy in thickness values arises because the two numbers refer to different components within the electrolyte assembly. Both values are correct in their respective contexts: The 12 μm value refers to the thickness of the ultrathin, porous polyethylene (PE) substrate used as a mechanical scaffold and processing carrier in the roll-to-roll fabrication. This substrate provides dimensional stability during coating and drying of the functional layers. The 40 μm value refers to the total thickness of the final freestanding MIGE (Tri-PUDA-*x*@LATP) membrane, which includes the PE substrate and all the coated functional layers (PUDA-10, PUDA-50, and PUDA-100).

To eliminate any potential confusion, we have clarified this distinction in the revised manuscript at the relevant locations:

In Section 2.1, the description of the fabrication process has been updated to explicitly state both thicknesses and their relationship (**Page 7**):

Compatible with roll-to-roll manufacturing, the MIGE is formed by sequential coating the functional layers onto an ultrathin (~12 μm), porous polyethylene (PE) substrate (see Supporting Information for details). The final freestanding MIGE membrane has a total thickness of approximately 40 μm.

9. Why does this polymer electrolyte exhibit superior oxidative stability compared to PEO? Additionally, in the LSV experiments used to evaluate the oxidative stability of the polymer electrolyte, what material was used to provide electronic conductivity in the electrode? Without an electronically conductive component in the electrode, proper redox reactions cannot occur, which may lead to significant overestimation of the electrochemical stability window. This is precisely why solid electrolytes are typically evaluated using electrodes containing a substantial amount of carbon conductive additive when determining electrochemical stability windows.

Author Reply: We thank the Reviewer's insightful question, which touches upon the fundamental chemical nature of our electrolyte and the rigorous methodology required for electrochemical evaluation. We have carefully considered both aspects and provide a detailed, point-by-point response below, supported by additional clarifications and corresponding revisions in the manuscript.

(1) The enhanced oxidative stability of the PUDA-based electrolyte results from a synergistic combination of its intrinsically robust polymer backbone and the extrinsic protection afforded by the high-loading of a wide-voltage-window ceramic filler. The polyurethane backbone, incorporating urea linkages (–NH–CO–NH–) and aromatic units from the DABA chain extender, possesses higher bond dissociation energy and greater structural stability against nucleophilic/oxidative attack compared to the polyether (–C–O–C–) backbone of PEO. This provides a fundamental, albeit modest, improvement to the intrinsic anodic limit (e.g., ***Adv. Mater. 2024, 36, 2409269. Nat. Commun. 2024, 15, 351.***) The most decisive factor is the exceptionally high and spatially graded loading of LATP filler, uniquely enabled by the ultra-viscoelastic PUDA matrix (>5000% strain). LATP is a well-known oxide solid electrolyte with a wide intrinsic electrochemical window (>5.0 V vs. Li^+/Li). Whereas conventional PEO-based composites suffer from film failure and poor adhesion beyond ~50 wt% LATP, our PUDA matrix cohesively binds >200 wt% filler, allowing us to architect a gradient with a dense, ceramic-rich cathode interface layer (PUDA-100). This layer acts as a robust, pinhole-free physical and electrochemical barrier that directly shields the underlying polymer from high-voltage oxidative decomposition.

(2) The Reviewer correctly emphasizes the paramount importance of ensuring proper electronic conduction at the working electrode when determining electrochemical stability windows (ESW). The standard practice for many solid-state systems, particularly brittle ceramics or composites with poor interfacial contact, is indeed to use a composite working electrode containing a significant amount of electronic conductor (e.g., carbon black). The rationale for employing a Li | Stainless Steel (SS) asymmetric cell configuration in our study for PEO-based and PUDA-based electrolytes is based on the following established principles and literature precedents for sof, adhesive polymer-based electrolytes:

When assembled under mild pressure or a brief thermal treatment (80°C for 1 h, as performed in our experiments), these soft, viscous polymers form an intimate, void-free interface with the inert SS current collector. The SS itself provides the necessary electronic conduction pathway. The measured oxidative current directly corresponds to the decomposition of the electrolyte at this SS | electrolyte interface, providing a valid assessment of its intrinsic anodic limit. This method is widely adopted and validated in high-impact studies on polymer-based solid electrolytes (see, e.g., ***Adv. Mater. 2024, 2403078. Energy Storage Mater. 2024, 65, 103091. Energy Environ. Sci., 2014, 7, 627–631. J. Am. Chem. Soc. 2024, 146, 6591–6603***).

Blending a brittle inorganic filler (like LATP) with carbon and a polymer binder to form a composite electrode could introduce additional variables, such as new interfacial impedance, porosity, and possible side reactions between carbon, binder, and the electrolyte, that might obfuscate the intrinsic stability of the material system under investigation. The progression of the oxidative stability window in our data is logically consistent with material composition: PEO (~3.8 V) < PEO-LATP (~4.2 V) < PUDA-50 (~4.4 V) < Tri-PUDA-*x*@LATP (~4.9 V). This trend aligns with the well-known PEO, the increasing LATP content and the formation of a denser ceramic-rich layer at the interface, corroborating the material-based argument rather than an artifact of the measurement technique.

(3) The Reviewer's meticulous reading is greatly appreciated. We apologize for the omission in the original manuscript description. We confirm that in the full cell fabrication for both LFP and NCM811 cathodes, the composite cathode slurry did contain LATP solid electrolyte powder as an ionic conductor to form a composite cathode. The correct weight ratio was active material : Super P carbon : PVDF binder : LATP powder = 7:1:1:1. The text in the Experimental Section has been corrected accordingly, and we thank the Reviewer for noting this oversight.

Textual Revisions in the Manuscript:

In Section 2.3, after presenting the LSV data, we have added (**Page 19**):

The enhanced oxidative stability originates from the synergistic combination of a more robust polyurethane backbone[12] and, dominantly, the formation of a dense, ceramic-rich protection layer at the cathode interface.

In the Supporting Information (Experimental Section):

Linear sweep voltammetry (LSV) was conducted using Li | electrolyte | stainless steel (SS) asymmetric cells at room temperature with a scan rate of 1 mV s^{-1}, ranging from the open-circuit voltage up to 7 V (vs. Li^+/Li). To ensure intimate interfacial contact, the assembled cells were subjected to a mild thermal treatment at 80°C for 1h prior to testing. For soft polymer-based electrolytes like PEO and PUDA, the SS electrode provides sufficient electronic conductivity for a reliable assessment of oxidative decomposition, a method well-established in the field.

The composite cathode slurry was prepared by thoroughly mixing active material ($LiFePO_4$, LFP or $LiNi_{0.8}Mn_{0.1}Co_{0.1}O_2$, NCM811), conductive carbon black (Super

P), poly(vinylidene fluoride) (PVDF) binder, and LATP solid electrolyte powder in N-methyl-2-pyrrolidone (NMP) at a mass ratio of 7:1:1:1.

10. Why were most of the cell performance data obtained using LFP cathodes? And why was NCM811 used only in the pouch-cell configuration? Furthermore, in Figure 5e, the capacity appears to increase for nearly 200 cycles—what is the underlying reason for this behavior?

Author Reply: We thank the Reviewer for raising these critical points, which allow us to clarify the experimental rationale and a key observed electrochemical feature. Our responses are detailed below, and corresponding clarifications and new data have been incorporated into the revised manuscript.

The extensive use of LFP cathodes in this study is intentional and based on the following considerations: LFP operates at a moderate voltage window (~3.4 V vs. Li^+/Li) and exhibits minimal interfacial side reactions (e.g., oxygen release, transition-metal dissolution). This allows us to isolate and highlight the mechanical and interfacial stability contributions of the MIGE, without the confounding effects of high-voltage-induced degradation. Therefore, stable long-term cycling with LFP directly validates the core advance of the MIGE: its ability to maintain conformal electrode contact and suppress Li-dendrite growth through its ultra-viscoelastic, graded architecture. In addition, LFP is a well-established, commercially relevant cathode with highly reproducible electrochemical behavior.

The demonstration of a stack-pressure-free NCM811 pouch cell was designed to highlight a key practical advantage of the MIGE: its ultra-viscoelasticity eliminates the need for external pressure (often >10 MPa for ceramic-based cells) to maintain interfacial integrity, thereby simplifying cell integration. We agree with the Reviewer that complementary coin-cell data would strengthen the narrative. Accordingly, we have performed additional long-term cycling tests of Li|NCM811 coin cells employing the Tri-PUDA-*x*@LATP electrolyte at both 30 °C and 60 °C (0.5 C).

The Reviewer has identified an important feature that directly reflects the dynamic interfacial behavior of our viscoelastic electrolyte. The observed capacity rise during the initial ~200 cycles at 30 °C is attributed to the progressive interfacial optimization enabled by the ultra-elastic PUDA matrix. At this near-ambient temperature and under low-rate cycling, the polymer chains gradually adapt to the electrode surfaces, increasing the electrochemically active contact area and reducing interfacial impedance. This autonomous "self-wetting" process is a characteristic advantage of highly viscoelastic polymers and contrasts sharply with the static, often poor, interface of brittle ceramics. After approximately 200 cycles, the interface reaches an optimal state, leading to the subsequent stable, long-term plateau observed for over 1700 cycles (capacity retention rate 89.3%). We have added a brief citation to prior reports of similar behavior in interface-adaptive solid electrolytes (e.g., ***Nat. Mater. 2025, 24, 907–916. Nat. Commun. 2023 14, 4077***).

Revised/Added Text in Main Manuscript:

We have added a brief justification in the revised Section 2.5 (**Page 23**):

LiFePO$_4$ (LFP) was selected as the primary cathode material for systematic evaluation due to its stable electrochemistry and minimal interfacial side reactions, allowing the mechanical and interfacial stability contributions of the MIGE to be clearly elucidated.

The long-term cycling performance of the Li|NCM811 coin cells at 0.5 C and 30 °C is provided in Supplementary Fig. S16 and briefly discussed in the revised Section 2.5 (**Page 25**).

When paired with a high-voltage NCM811 cathode (1C = 220 mA g^{-1}), the solid-state Li|Tri-PUDA-*x*@LATP|NCM811 cell achieved an initial discharge capacity of 179.4 mAh g^{-1} and maintains an average Coulombic efficiency of 96.5% over 200 cycles at 0.5C, significantly outperforming the cell with the PUDA-50@LATP electrolyte (**Fig. S16**). These results demonstrate the robust cycling stability of the MIGE-based cell even under near-ambient operating conditions.

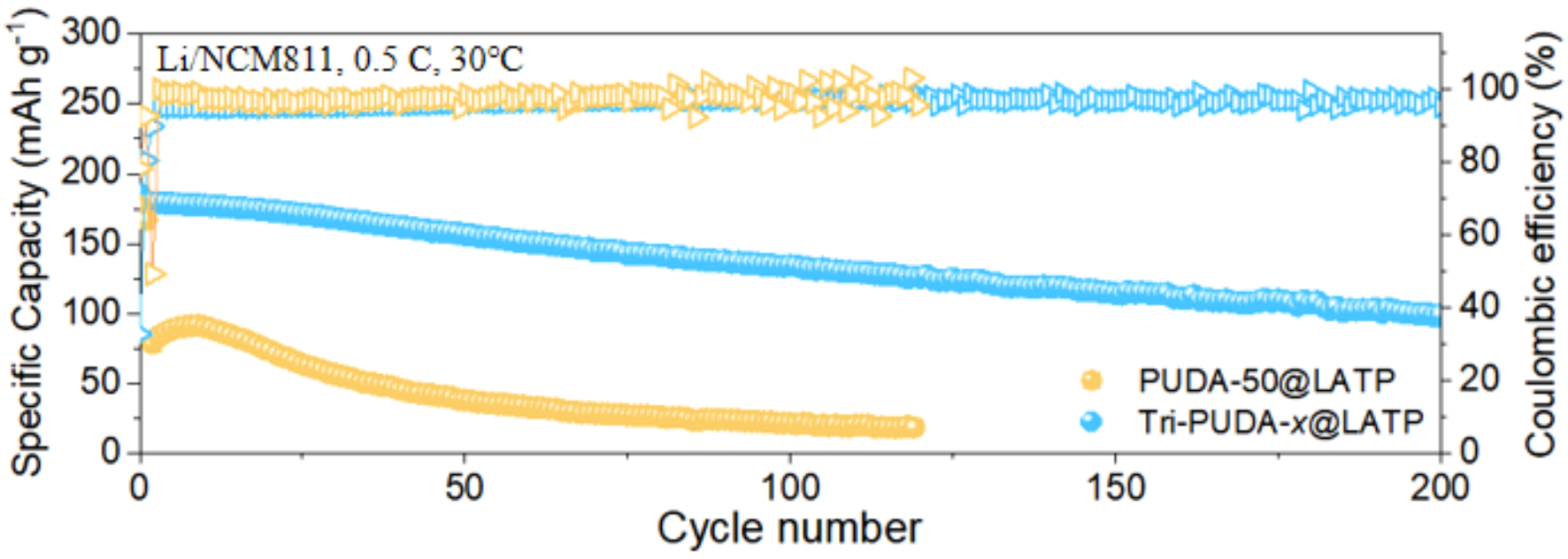

Fig. S16 | Cycling stability of solid-state Li/NCM811 cells with PUDA-50@LATP and Tri-PUDA-*x*@LATP at 0.5C, 30°C.

To provide a clear explanation for readers, we have added the following sentences in Section 2.5 where Fig. 5e is discussed (**Page 24**):

The gradual capacity increase during the initial ~200 cycles at 30 °C reflects progressive interfacial optimization enabled by the viscoelastic PUDA matrix. Under near-ambient conditions, the ultra-elastic electrolyte dynamically improves solid-solid contact, which reduces interfacial resistance and gradually activates additional capacity from the LFP cathode. This self-adapting process stabilizes after ~200 cycles, leading to highly stable long-term cycling. The occasional exceedance of 100 % Coulombic efficiency is attributed to the reversible recovery of previously isolated lithium, facilitated by the continuous interfacial improvement and dynamic self-healing of the PUDA framework, a phenomenon consistent with prior reports on adaptive solid-state interfaces [32, 33].

References:

32. Wang, C. et al. The influence of pressure on lithium dealloying in solid-state and liquid electrolyte batteries. *Nat. Mater.* **24**, 907-916 (2025).
33. Lu, P. et al. Realizing long-cycling all-solid-state Li-In||TiS$_2$ batteries using Li$_{6+x}$M$_x$As$_{1-x}$S$_5$I (M=Si, Sn) sulfide solid electrolytes. *Nat. Commun.* **14**, 4077

(2023).

11. It is highly unusual that none of the cathode electrodes used in this study contains a solid electrolyte. How can the cells operate without a solid electrolyte in the composite cathode? Does the polymer from the polymer electrolyte layer infiltrate into the cathode during cell fabrication? Or is the cathode loading so low that electrochemical cycling is possible simply through contact with the electrolyte membrane, even without electrolyte inside the electrode? If the latter is the case, this configuration is not practical at all. For realistic assessment, the system should be demonstrated at higher practical areal loadings (e.g., ~3–4 mAh cm^{-2}), and therefore, data obtained from electrodes without a solid electrolyte are difficult to accept.

Author Reply: We sincerely thank the Reviewer for their careful reading and constructive comment regarding the composition of the cathode slurry. The Reviewer's observation is correct, and we apologize for the omission in the original manuscript. All composite cathodes did contain LATP solid electrolyte powder. The precise mass ratio used was: active material (LFP or NCM811) : Super P : PVDF : LATP = 7:1:1:1. This formulation ensures continuous Li^+ pathways within the cathode composite while maintaining adequate electronic percolation and mechanical intergrity. We have now explicitly stated this composition in the Experimental Section of the Supporting Information, and we thank the Reviewer for noting this oversight.

The Reviewer raises an important point regarding the practical difficulty of achieving complete electrolyte penetration into thick cathodes (e.g., 3-4 mAh cm^{-2}). We fully agree that optimizing the cathode microstructure, especially ensuring efficient ion/electron transport and maintaining intimate solid–solid contact throughout thick electrodes, remains a critical challenge for solid-state batteries. In the present study, we focused on developing and validating the MIGE as a versatile electrolyte platform, and therefore employed conventional composite cathodes with moderate areal loadings. The excellent cycling performance (>1000 cycles with 74.4 % capacity retention for LFP, stable high-voltage operation with NCM811) under zero-external-stack-pressure conditions validates the MIGE's ability to maintain intimate interfacial contact and efficient ion transport at the electrolyte/electrode interface.

We acknowledge that further dedicated cathode engineering, such as the use of in-situ polymerization, gradient electrode architectures, or higher-fluorine-content binders, will be essential to fully exploit the potential of the MIGE in high-energy-density cells. These approaches, however, represent a separate research direction that extends beyond the scope of this manuscript, which is centered on the material innovation, gradient electrolyte design, and interfacial decoupling enabled by the ultra-viscoelastic PUDA matrix. To provide a balanced perspective, we have added a brief discussion on this point in the Conclusion section.

To avoid any potential misunderstanding, we have further emphasized in the Introduction and Conclusion that the primary contribution of this study is: The molecular design of an ultra-viscoelastic polyurethane (PUDA) via dual hydrogen-bonding extenders (DBDA/ADH). The construction of a mechanically

integrated gradient electrolyte (MIGE) that spatially decouples anode and cathode interfacial requirements. Demonstration of exceptional cyclability (>7500 h in Li/Li symmetric cells) under low-pressure assembly conditions. A scalable, air-relatively stable, solution-processable fabrication route compatible with roll-to-roll manufacturing

Revised/Added Text in Main Manuscript:

In the Supporting Information (Experimental Section):

The composite cathode slurry was prepared by thoroughly mixing active material ($LiFePO_4$, LFP or $LiNi_{0.8}Mn_{0.1}Co_{0.1}O_2$, NCM811), conductive carbon black (Super P), poly(vinylidene fluoride) (PVDF) binder, and LATP solid electrolyte powder in N-methyl-2-pyrrolidone (NMP) at a mass ratio of 7:1:1:1.

In Conclusion (Section 3, **Page 31**):

While this work demonstrates a high-performance electrolyte platform that operates without external stack pressure, we note that the realization of practical high-energy-density SSLMBs will also require advances in composite-electrode design, particularly for thick electrodes. Achieving uniform electrolyte distribution and maintaining durable ionic/electronic contact throughout such electrodes remain important challenges. Future work will focus on integrating the MIGE with advanced electrode architectures (e.g., using in-situ polymerization or gradient electrode composites) to simultaneously address electrolyte and electrode microstructural requirements.

In Introduction (Section 1, **Page 5**):

This work establishes a new design paradigm for solid electrolytes that directly addresses the core mechano-electrochemical coupling issue. The resulting MIGE platform offers a scalable, solution-processable route to high-performance, pressure-free SSLMBs.

12. There are several capitalization errors in units (e.g., MPa), which appear both in the figures and the main text, and should be corrected.

Author Reply: We thank the Reviewer for the careful reading and the constructive comment. We have now systematically corrected all unit symbols throughout the manuscript and figures to adhere strictly to the International System of Units (SI) conventions

13. Superscripts for numbers and units appear to have been inserted by direct copy-and-paste (e.g., from ChatGPT or similar tools) rather than using proper word-processing formatting. As a result, their appearance is inconsistent and should be revised.

Author Reply: We sincerely thank the Reviewer for this meticulous observation regarding the formatting consistency of superscripts throughout the manuscript. The

comment is well taken, and we acknowledge that during the final editing and integration of text, certain numerical values and units may have been transferred in a format that appears inconsistent with the rest of the typesetting. Thank you again for the constructive feedback.

14. Figure 6 appears to be partially cut off.

Author Reply: We sincerely thank the Reviewer for the careful inspection and valuable comment. The Reviewer is absolutely correct that a roller of the coater in the original schematic was partially truncated. We have now updated Fig. 6 to present the complete manufacturing line (**Page 29**). Thank you again for pointing out this important presentational issue.

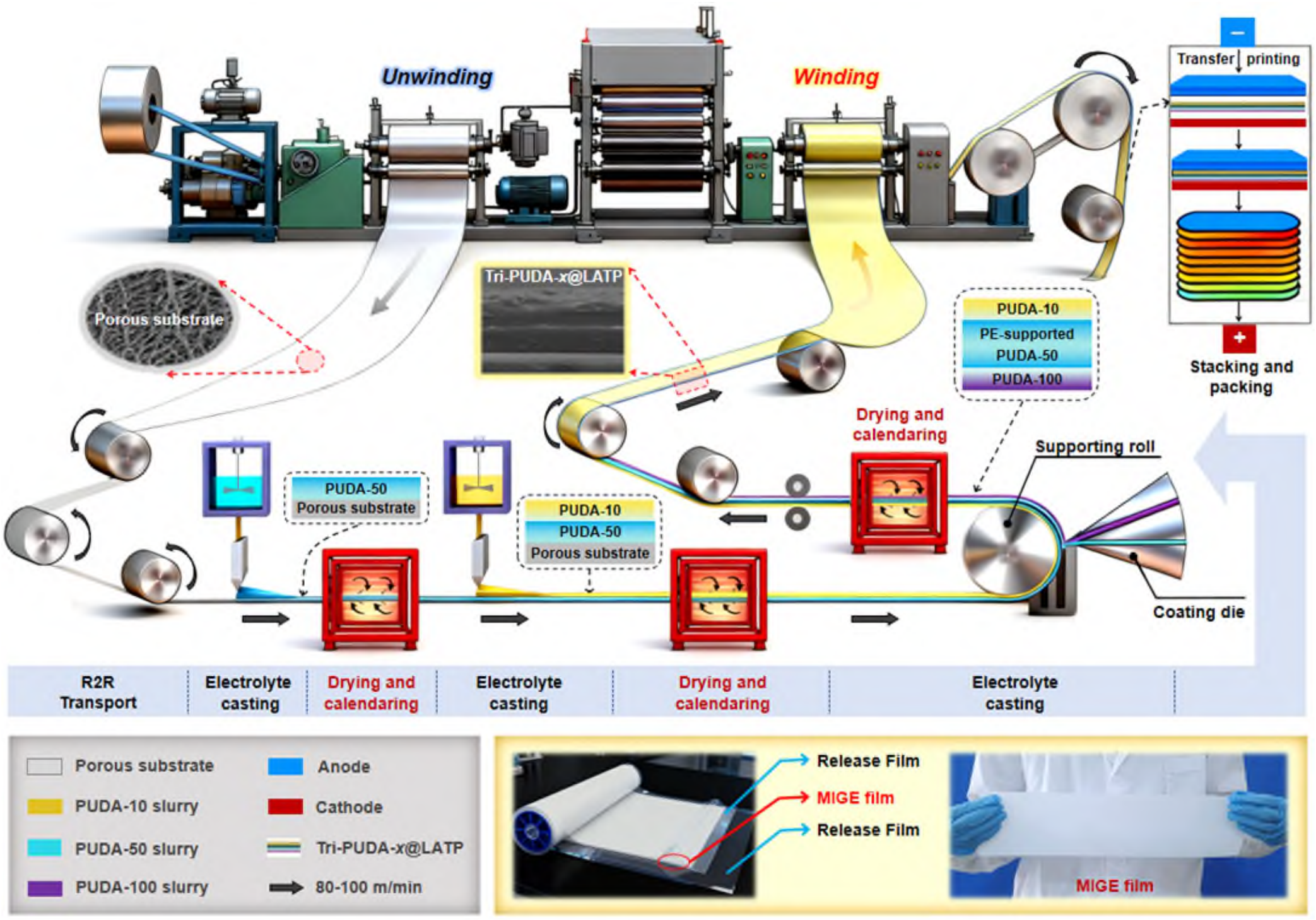

Fig. 6 | Schematic illustration of the roll-to-roll (R2R) manufacturing process for multilayer coating and transfer printing of the MIGE for solid-state lithium batteries.

Reviewer #2: *We thank the authors for their detailed responses. The manuscript has been significantly improved and most of the previous questions have been addressed. To further strengthen the clarity and robustness of the study, we would like to suggest clarification on the following point:*

As mentioned in the response to Comment 3, conducting a SS||SS symmetric cell measurement could further support the evaluation of the interfacial resistance at the organic/inorganic interface and make the argument more convincing.

Author Reply: We sincerely thank the Reviewer for the constructive suggestion to further support our claim regarding the low interfacial resistance of the organic–inorganic interphase. Following the method described in *ACS Omega 2021, 6, 16187–16193*, we have performed additional experiments to evaluate the interfacial resistance between the PUDA matrix and LATP. While the referenced work focuses on a gel polymer system with limited elongation (<250%) where filler effects on polymer crystallinity are minimal, our approach extends this methodology to a highly viscoelastic, hydrogen-bonded polymer matrix that simultaneously influences interfacial contact, polymer crystallinity, and filler dispersion. To address the Reviewer's comment, we conducted SS||SS symmetric cell measurements to isolate and quantify the PUDA/LATP interfacial resistance.

Experimental Details:

To measure the interfacial resistance between PUDA and LATP, we fabricated a three-layer laminate consisting of two PUDA films (polymer without filler) sandwiching a dense LATP ceramic plate ($Li_{1.3}Al_{0.3}Ti_{1.7}(PO_4)_3$). The LATP plate was prepared by pressing LATP powder into a pellet (diameter: 16 mm, thickness: 2 mm), sintering at 900 °C for 4 h to enhance density, polishing both surfaces (thickness: 0.95 mm), and coating with Au (dried at 80 °C for 1 h) to ensure good electrical contact. The PUDA films were prepared with a thickness of ≈150 μm and an area of 2 cm^2.

The PUDA/LATP/PUDA stack was assembled in a symmetric cell with two stainless steel (SS) blocking electrodes. AC impedance spectroscopy was performed over a frequency range of 1000 kHz to 1 Hz with a 10 mV amplitude at various temperatures (30, 40, 50, 60, and 70 °C). The total resistance of the three-layer stack (R_{tot}) was obtained from the high-frequency intercept of the Nyquist plot. The resistance of PUDA film (R_{PUDA}) and the LATP plate (R_{LATP}) were measured separately under identical conditions. The total interfacial resistance ($R_{int,tot}$) was then calculated as: $R_{int,tot} = R_{tot} - R_{PUDA} - R_{LATP}$. The resistance of a single PUDA/LATP interface (R_{int}) is half of this value.

The measured resistances of the individual components and the three-layer stack are shown in **Fig. S12.** The interfacial resistance (R_{int}) decreases with increasing temperature, indicating thermally activated Li^+ transfer across the PUDA–LATP interface. The activation energy for ion transport across the PUDA–LATP interface (0.28 eV) is lower than that of either the PUDA (0.35 eV) or the LATP (0.32 eV). This result demonstrates that the interface engineered in our system facilitates ion hopping between the two phases. The low interfacial activation energy is attributed to the

synergistic effects of the dynamic hydrogen-bonded network in PUDA, which ensures intimate contact and reduces the local energy barrier for ion exchange, combined with the presence of ionic liquid that plasticizes the boundary region. These findings validate our design strategy and support the conclusion that the percolation network formed at the optimal filler loading enables efficient ion conduction via interconnected pathways with minimal interfacial penalty.

Corresponding Revisions in the Manuscript:

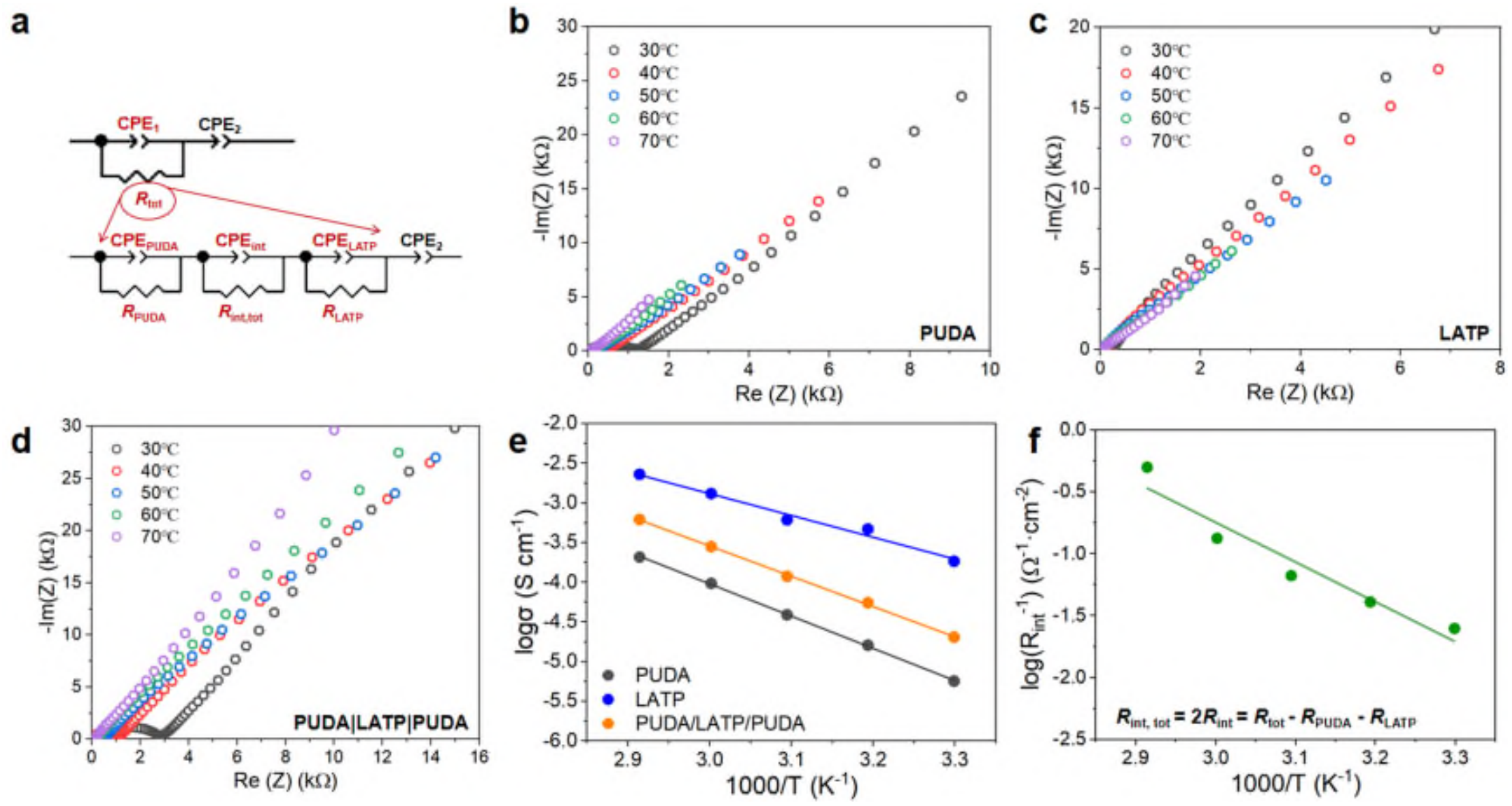

Fig. S12 | Interfacial resistance analysis of the PUDA/LATP interface. a, Equivalent circuit model of the SS|PUDA|LATP|PUDA|SS symmetric cell. Constant phase elements (CPEs) are used instead of capacitances to fit the impedance spectra. **b-d**, Nyquist plots of SS|PUDA|SS, SS|LATP|SS, and SS|PUDA|LATP|PUDA|SS cells measured at various temperatures. **e**, Ionic conductivity of PUDA, LATP, and the PUDA/LATP/PUDA stack as a function of temperature. **f**, Arrhenius plot of $1/R_{int}$, normalized by the contact area of the PUDA/LATP interface.

To directly probe the ion transfer kinetics at the organic-inorganic interface, the interfacial resistance between PUDA and a dense LATP plate was measured using a symmetric SS|PUDA|LATP|PUDA|SS cell (**Fig. S12**). The total resistance of the three-layer stack was deconvoluted by subtracting the separately measured resistances of the two PUDA layers and the LATP plate, yielding the interfacial resistance (R_{int})[32]. The Arrhenius analysis of R_{int}^{-1} reveals an activation energy of only 0.28 eV, which is significantly lower than those of PUDA (0.35 eV) and LATP (0.32 eV). This low activation energy indicates that the PUDA-LATP interface promotes Li^+ exchange between the two phases. Such favorable interfacial kinetics, combined with the continuous percolation network, explains the enhanced ionic conductivity observed at the optimal filler loading of 50 wt%.

References

32. Ock, J. et al. Transport properties of flexible composite electrolytes composed of $Li_{1.5}Al_{0.5}Ti_{1.5}(PO_4)_3$ and a poly(vinylidene fluoride-co-hexafluoropropylene) gel containing a highly concentrated $Li[N(SO_2CF_3)_2]$/sulfolane electrolyte. *ACS Omega* **6**, 16187-16193 (2021).
